# Immunogenicity and Safety of the 13-Valent Pneumococcal Conjugate Vaccine *versus* the 23-Valent Polysaccharide Vaccine in Unvaccinated HIV-Infected Adults: A Pilot, Prospective Controlled Study

**DOI:** 10.1371/journal.pone.0156523

**Published:** 2016-06-03

**Authors:** Francesca Lombardi, Simone Belmonti, Massimiliano Fabbiani, Matteo Morandi, Barbara Rossetti, Giacinta Tordini, Roberto Cauda, Andrea De Luca, Simona Di Giambenedetto, Francesca Montagnani

**Affiliations:** 1 Institute of Clinical Infectious Diseases, Catholic University of the Sacred Heart, Largo Agostino Gemelli 8, Rome, Italy; 2 Department of Medical Biotechnologies, University of Siena, Viale Bracci 16, Siena, Italy; 3 University Division of Infectious Diseases, Hospital Department of Specialized and Internal Medicine, Viale Bracci 16, Siena, Italy; 4 Division of Infectious Diseases, Department of Internal Medicine, San Gerardo Hospital, University of Milano-Bicocca, Monza, Italy; Azienda ospedaliero-universitaria di Perugia, ITALY

## Abstract

**Objectives:**

Definition of the optimal pneumococcal vaccine strategy in HIV-infected adults is still under evaluation. We aimed to compare immunogenicity and safety of the 13-valent pneumococcal conjugate vaccine (PCV13) *versus* the 23-valent polysaccharide vaccine (PPSV23) in HIV-infected adults.

**Methods:**

We performed a pilot, prospective controlled study enrolling HIV-infected pneumococcal vaccine-naïve outpatients, aged 18–65 years with CD4 counts ≥200 cells/μL. Eligible subjects were recruited into two parallel groups: group 1 (n = 50) received two doses of PCV13 eight weeks apart, and group 2 (n = 50) received one dose of PPSV23, as part of their standard of care. Anti-pneumococcal capsular polysaccharide immunoglobulin G concentrations were quantified by ELISA at baseline, 8, 24 and 48 weeks. Clinical and viro-immunological follow-up was performed at the same time points. Unvaccinated, age-matched HIV-negative adults (n = 100) were also enrolled as baseline controls.

**Results:**

Pre-vaccination specific IgG titers for each pneumococcal antigen did not differ between study groups but they were constantly lower than those from the HIV-negative controls. After immunization, significant increases in IgG titers were observed in both study groups at each time point compared to baseline, but response to serotype 3 was blunted in group 1. Antibody titers for each antigen did not differ between study groups at week 48. Overall, the proportion of subjects achieving seroprotection and seroconversion to all serotypes was comparable between groups. A marked decrease in IgG levels over time was observed with both vaccines. No relevant adverse reactions were reported in either group.

**Conclusions:**

In this population with favorable immune profile, no relevant differences were observed in immunogenicity between PCV13 and PPSV23. Both vaccines were safe and well tolerated.

**Trial Registration:**

ClinicalTrials.gov NCT02123433

## Introduction

HIV-infected subjects have an increased risk for invasive pneumococcal diseases (IPD), frequently leading to hospitalization and death [1–4]. Therefore, efforts to reduce the incidence of IPD in this population constitute a clinically relevant issue even in the era of the highly active antiretroviral therapy (HAART) [5, 6]. Since the 80’s, the US Centers for Disease Control have recommended using the 23-valent pneumococcal polysaccharide vaccine (PPSV23) for immunocompromised subjects [7]. As a plain polysaccharide vaccine, PPSV23 does not elicit T cell-dependent immune responses: the absence of memory B cells reduces the duration of its protection and re-vaccinations are required also in the immunocompetent host [8]. However, PPSV23 booster doses have been reported to cause “hyporesponsiveness” [8]. This limitation makes PPSV23 less suitable for HIV-infected individuals, mainly for those in advanced stages of immunodeficiency.

Several studies have shown that humoral response to PPSV23 is weaker in HIV-infected adults compared to an age-matched immunocompetent population [9–11] and that patients with AIDS fail to be protected [12].

In 2000, protein-conjugated pneumococcal vaccines (PCVs) were licensed and have been widely adopted for pediatric use: the 7-valent conjugate vaccine formulation (PCV7) has been demonstrated to greatly decrease the incidence of pneumococcal infections due to vaccine serotypes [13]. In 2010 the FDA licensed a 13-valent conjugate vaccine formulation (PCV13) that covers 6 additional serotypes. PCVs induce a T-cell-dependent response that stimulates long-lived memory cells [14], prevents B-cell depletion and produces high affinity antibodies. PCVs may also prime the immune system for more rapid and improved responses to booster doses [15].

Thus, vaccination with PCV could be an optimal primary prophylaxis strategy for HIV-infected patients. Indeed, in randomized trials of HIV-infected adults PCV7 was shown to have high efficacy by itself [16] and to be more immunogenic than PPSV23, eliciting a higher and persistent ≥4-fold increase in antibodies against 6B and 9V serotypes in a larger proportion of HIV patients [17]; moreover, revaccination with PCV7 was reported to elicit a better serologic response than PPSV23 in HIV-infected adults who had received PPSV23 five or more years earlier [18].

To date, international guidelines recommend that HIV+ patients aged ≥19 years, who are PCV13 naïve should receive a single dose of PCV13. PPSV23 should be given ≥8 weeks after PCV13; a second dose of PPSV23 should be given 5 years later. For those who previously received PPSV23, PCV13 should be administered ≥1year after the last PPSV23 dose [19].

To the best of our knowledge a direct comparison of the immunogenicity of PCV13 versus PPSV23 in HIV-infected adults is still lacking.

The present study aimed to investigate the immunogenicity and safety of administering two doses of PCV13 to unvaccinated HIV-infected adults and to compare the immunological response over time with that elicited by PPSV23.

## Patients and Methods

### Study design

From November 2011 to April 2012, 100 HIV-infected adult outpatients who had never been vaccinated with any pneumococcal vaccine were prospectively enrolled in two parallel studies, each performed at two Infectious Diseases Clinical Centers in Central Italy (University Division of Infectious Diseases in Siena and Institute of Clinical Infectious Diseases, Catholic University of the Sacred Heart in Rome). The first study was interventional and prospectively enrolled patients who were assigned to receive the 13-valent pneumococcal vaccine (PCV13 Prevenar13®, Wyeth Vaccines), while the second study prospectively enrolled patients who were receiving their first routine vaccination with PPSV23 (Pneumovax®, Sanofi Pasteur Msd Spa), which was considered the standard of care based on guidelines viable during the study period.

For both studies, inclusion criteria were: age ≥18 years and CD4 cell counts ≥200cells/μL. Exclusion criteria were: age >65 years, pregnancy, non-HIV related immunosuppression, current immunomodulatory therapy, any type of previous pneumococcal vaccination, antibiotic treatment in the previous 7 days and presence of acute infectious diseases.

Three hundred and fifty HIV-infected patients were assessed for eligibility by medical researchers. A total of 245 patients fully met criteria and were offered to participate by the medical researchers during one of the routine follow up visits, 100 accepted to participate to one of the two parallel studies. The first group of patients (n = 50) received two doses of PCV13 eight weeks apart and the second group (n = 50) one dose of PPSV23 as part of their routine care.

A third age-matched group of HIV-negative outpatients (n = 100) was also recruited as control comparator of pre-immunization IgG antibody levels; control subjects did not receive any vaccination.

The study was approved by the local Institutional Ethics Committees, considering the PCV13 vaccination procedure as interventional and experimental. It should be noted that at the time of the study only PPSV23, not PCV13, was recommended as part of the routine care vaccinations for HIV-infected adults [20]. All subjects provided written informed consent before they were enrolled in each study group. Follow-up was completed in May 2013; while the study ended in November 2013 when outcomes collection was completed.

### Patients follow-up

At baseline (day 0, time of first vaccine dose administration,), demographic, clinical and laboratory characteristics were collected by patients interview and using clinical records.

Follow-up visits were scheduled at 8 (time of second PCV13 dose in group 1), 24 and 48 weeks. At each visit peripheral venous blood samples were collected to assess humoral immune response; CD4+ T cell count and plasma HIV-RNA were also monitored.

After each vaccine administration, short- (30 minutes), medium- (≤5 days) and long-term adverse reactions were monitored.

At baseline, a single peripheral venous blood sample was collected from HIV-negative controls to assess the natural humoral immune response to pneumococcal polysaccharides. Demographic and clinical variables were also collected.

### Determination of anticapsular IgG antibodies against pneumococcal serotypes

Quantitation of specific IgG levels for 12 serotypes shared by PCV13 and PPSV23 (1, 3, 4, 5, 6B, 7F, 9V, 14, 18C, 19F, 19A and 23F) and for the additional serotype contained only in PCV13 (6A) was performed on patients’ sera, by an ELISA method, according to the World Health Organization (WHO) procedure [21]; the protocol was modified by using antigens from the Staten Serum Institute (SSI Diagnostica, Hillerød, Denmark), in place of ATCC (American Type Culture Collection, Manassas, VA, US) antigens, as we previously described [22].

The new human pneumococcal standard reference serum, 007sp, kindly given by Dr. Mustafa Akkoyunlu (US, FDA) [23] and a panel of 12 quality controls (NIBSC cod:12/278; Potters Bar, Hertfordshire, UK) were employed.

Antigen-specific IgG levels were expressed as geometric mean concentrations (GMCs μg/mL) for each pneumococcal serotype. The generally used threshold of 0.35μg/mL (accepted by the WHO for assessment of vaccine efficacy against IPD) [24] and a more conservative threshold of 1 μg/mL (potentially more relevant for long-term protection and previously used in studies with HIV-infected children)[25, 26] were chosen as cut-offs for defining seroprotection levels. Moreover, in a recent study individual serotype-specific correlates of protection were established, except for serotype 5 [27]. We carried out further data analyses also using these novel serotype-specific seroprotection values.

Seroconversion was defined as a ≥ 4-fold post-vaccination increase of the antigen-specific IgGs over the pre-vaccination levels.

### Statistical analysis

At the time of the study design no data were available regarding PCV13 immunogenicity in HIV positive children or adults. As a consequence, to detect differences in immunological response to PCV13 versus PPSV23 in HIV-positive adults, sample size estimation was based on data obtained by vaccination of HIV-infected children with PCV7 and vaccination of HIV-infected adults with PPSV23. A study by Thanee et al. [28] reported that 85–98% of HIV-infected children reached an appropriate serotype-specific IgG antibody concentration after receiving a dose of PCV7. Conversely, a study by Hung at al. [10] estimated that about 40% of HIV-positive adults had a significant antibody response to PPSV23. On the basis of these data, we expected to observe a serological response in at least 70% of PCV13-vaccinated HIV-infected adults. Therefore, based in an expected antibody response in 70% of PCV13-vaccinated and 40% of PPSV23-vaccinated HIV-infected patients, we estimated that 50 patients had to be included in each vaccination group to detect a significant difference between groups at a probability level of 95% with a power of 81.5%. For baseline characteristics, categorical variables were compared using a chi-square test or Fisher's exact test, as appropriate. Comparisons of continuous variables were based on Student’s t-test. The antibody concentrations were log-transformed to approximate normal distributions. The GMCs were obtained by taking the antilogarithm of the means of the log-transformed values. Corresponding two-sided 95% confidence intervals (CIs) for the GMCs were constructed by back-transforming the 95% CI for the mean of logarithmically transformed assay results computed using the Student t distribution. Student’s t-test was used for testing significance on the log-transformed antibody concentrations. General Linear Modeling Repeated Measures ANOVA (GLM-ANOVA) was used to compare the log-transformed antibody concentrations over the study period within and between the two vaccinated groups; the Greenhouse-Geisser correction was applied because the sphericity assumption was violated [29, 30]. Bonferroni method was used for the multiple comparisons of the mean involved. In the GLM-ANOVA we did not add any covariate. To compare the proportions of subjects reaching seroprotective levels and subjects who seroconverted between the two vaccine groups, Pearson’s chi-square test was used; the Wilson method was employed to compute the confidence intervals of the proportions. Differences between groups were considered significant at the conventional p level <0.05. Analyses were performed using the SPSS software package (version 18.0 Chicago, IL).

### Ethics Statement

This study was reviewed and approved by institutional review boards at both of participating sites (Ethic committee Area Vasta Sud Est, Azienda Ospedaliera Universitaria Senese, Siena, Italy and Ethic committee of Catholic University of Sacred Heart, Rome, Italy). The project was approved as two distinct parts: an interventional and experimental study for PCV13 vaccinated patients (Eudract number 2011-004518-40 –Protocol code PCV13-HIV2011; ClinicalTrials.gov n.NCT02123433) and an observational study in PPSV23 vaccinated patients and HIV negative control (Protocol Code PNEUMO-HIV 2011).

## Results

### Study population

**Fig 1:**
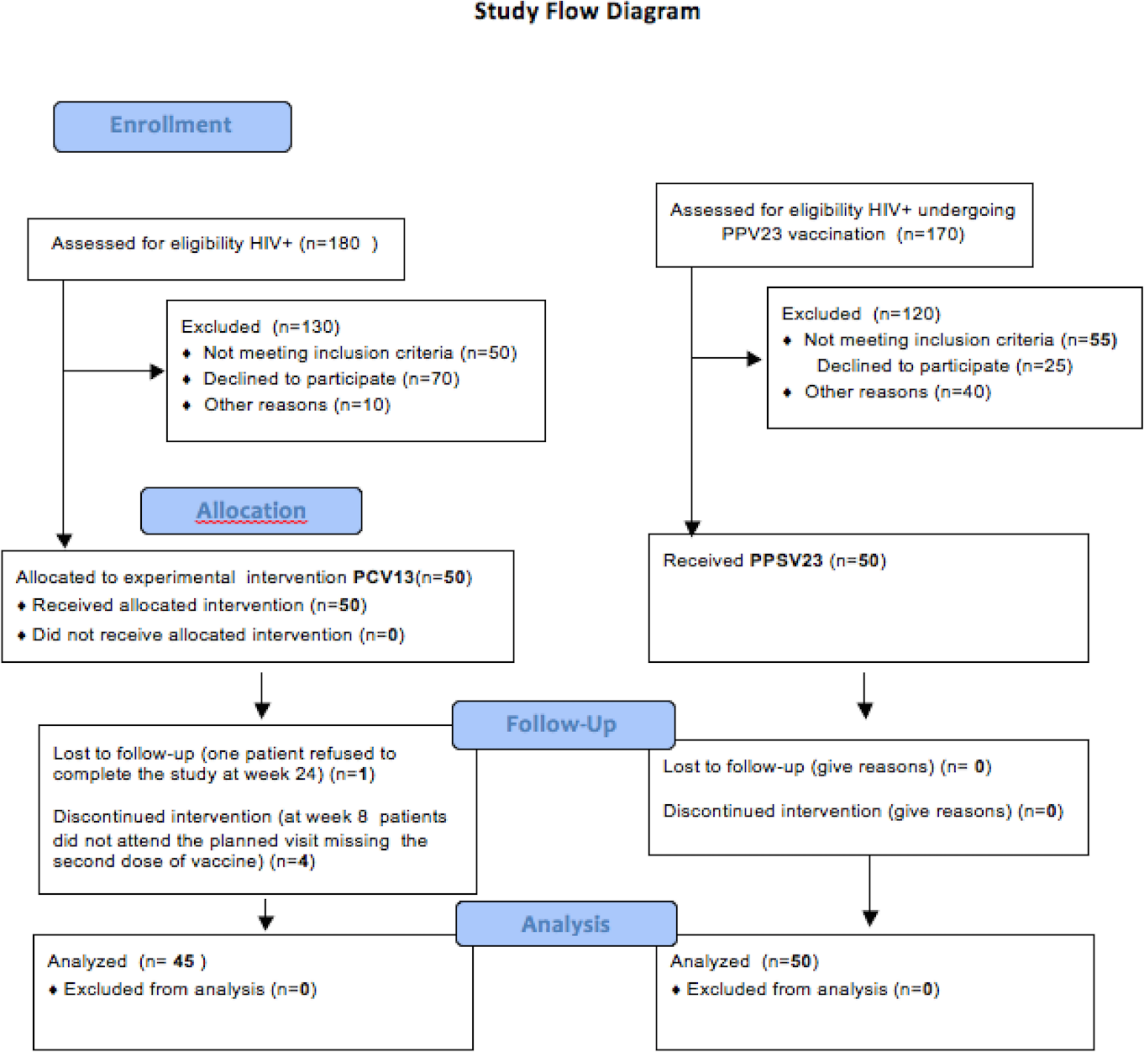
Flow chart illustrating the entire study population. CONSORT flow diagram.

A total of 100 HIV-positive subjects were enrolled: 50 in group 1 to receive PCV13 and 50 in group 2 to receive PPSV23 (Fig 1).

Seventy-two percent were males, 92% were Caucasian, 99% received stable antiretroviral therapy, 88% had HIV-RNA ≤50 copies/mL None had acute infections.

Baseline main characteristics were homogeneous between groups, except for the proportion of non Caucasian subjects, which was slightly higher in group 1 (Table 1A).

**Table 1:** Characteristics of the study population at enrollment: (A) comparison between the two groups of HIV+ patients and (B) between HIV+ enrolled patients and HIV- baseline controls.

A			
	**Group 1 (PCV13) n = 50**	**Group 2 (PPSV23) n = 50**	***p***
**Male, n (%)** ^**a**^	34 (68)	38 (76)	*0*.*373*
**Age, mean (SD)** ^**b**^	43.9 (9,05)	45.6 (10,5)	*0*.*394*
**Non Caucasian, n (%)** ^**a**^	7 (14)	1 (2)	***0*.*027***
**Body mass index (Kg/m**^**2**^**), mean (SD)** ^**b**^	23.9 (4.2)	23.2 (3.6)	*0*.*352*
**Years from HIV diagnosis, mean (SD)** ^**b**^	11.0 (7.6)	12.4 (10.2)	*0*.*434*
**Years from HAART initiation, mean (SD)** ^**b**^	7.8 (6.11)	10.8 (15.2)	*0*.*197*
**Risk factor, n (%)** ^**a**^			*0*.*44*
**Heterosexual**	16 (32)	21 (42)	* *
**Homo/bisexual**	21 (42)	20 (40)	* *
**Injecting drug use**	7 (14)	7 (14)	* *
**Other /Unknown**	6 (12)	2 (4)	* *
**Nadir CD4 count cell/μL, mean (SD)** ^**b**^	197.9 (132.4)	213.7 (194.3)	*0*.*635*
**CD4 count, mean (SD) cell/μL** ^**b**^	591.2 (226.5)	639.4 (302.5)	*0*.*371*
**CD4 cell count category, n (%)** ^**a**^			*0*.*788*
**200–350**	6 (12)	7 (14)	* *
**351–500**	15 (30)	16 (32)	* *
**>500**	29 (58)	27 (54)	* *
**Viral load ≤50 copies/μL, n (%)** ^**a**^	42 (84)	46 (92)	*0*.*218*
**Receipt of HAART, n (%)** ^**a**^	49 (98)^§^	50 (100)	*0*.*315*
**Antiretroviral regimen, n (%)**			*0*.*776*
**PI-based** ^**a**^	32 (64)	34 (68)	* *
**NNRTI-based** ^**a**^	11 (22)	12 (24)	* *
**Other** ^**a**^	7 (14)	4 (8)	* *
**Co-morbidities, n (%)**			* *
**Diabetes** ^**a**^	2(4)	1(2)	*0*.*558*
**Cardiovascular disease** ^**a**^	3 (6)	5 (10)	*0*.*461*
**Neoplasm** ^**a**^	0 (0)	1(2)	*0*.*315*
**Hepatitis co-infection (HCV or HBV)** ^**a**^	10 (20)	6 (12)	*0*.*275*
**Recent hospitalization, n (%) (last 12 months)** ^**a**^	2 (4)	4 (8)	*0*.*4*
**Cohabitation with children, n (%)** ^**a**^	7 (14)	9 (18)	*0*.*585*
**Age of cohabiting children, n (%)** ^**a**^			* 0*.*848*
**<3 years**	1 (14.3)	1 (11.1)	* *
**4–16 years**	6 (85.7)	8 (88.9)	* *
**Past AIDS-defining events, n (%)** ^**a**^	11 (22)	18 (36)	*0*.*123*
Smokers*, n (%) ^**a**^	30 (60)	33 (66)	*0*.*534*
Alcohol consumers**, n (%) ^**a**^	6 (12)	4 (8)	*0*.*505*
**B**			
** **	**HIV+ (Group 1+2) n = 100**	**HIV- n = 100**	***p***
**Male, n (%)** ^**a**^	72 (72)	52 (52)	***0*.*04***
**Age, mean (SD)** ^**b**^	44.7 (9.7)	42.4 (13.2)	*0*.*147*
**Non Caucasian, n (%)** ^**a**^	8 (8)	7 (7)	*0*.*788*
**Body mass index (Kg/m**^**2**^**), mean (SD)** ^**b**^	23.6 (3.9)	25.1 (5.4)	***0*.*037***
**Co-morbidities, n (%)**			* *
**Diabetes** ^**a**^	3 (3)	1 (1)	*0*.*312*
**Cardiovascular disease**^**a**^	8 (8)	13 (13)	*0*.*249*
**Neoplasm** ^**a**^	1(1)	5 (5)	*0*.*097*
**Hepatitis co-infection (HCV or HBV)** ^**a**^	16 (16)	39 (39)	***<0*.*001***
**Recent Hospitalization, n (%) (last 12 months)** ^**a**^	6 (6)	8 (8)	*0*.*579*
**Cohabitation with children, n (%)** ^**a**^	16 (16)	33 (33)	***0*.*005***
**Age of cohabiting children, n (%)** ^**a**^			*0*.*121*
**<3 years**	2 (12.5)	11 (33.3)	*** ***
**4–16 years**	14 (87.5)	22 (66.7)	* *
**Smokers** *, n (%) ^**a**^	63 (63)	30 (30)	***<0*.*001***
Alcohol consumers**, n (%) ^**a**^	10 (10)	21 (21)	***0*.*032***

**Abbreviation** SD, Standard Deviation; HBV Hepatitis B Virus; HCV, Hepatitis C Virus; HAART, Highly Active Antiretroviral Therapy; PI, Protease Inhibitors; NNRTI, Non-Nucleoside Reverse Transcriptase Inhibitors.

^a^ Chi–squared test/ Fisher’s exact test

^b^ Student’s t test

^§^ One patient started HAART at week 8

* active smoker (≥100 cigarettes in the last year)

** alcohol consumption ≥ 10 Alcholic Unit/week

Several pre-immunization characteristics of the HIV+ patients were also comparable with those of the HIV-negative subjects although the HIV+ subjects were more frequently male and active smokers and had a lower mean body mass index; they were less frequently alcohol consumers, infected with hepatitis viruses and cohabiting with children (Table 1B).

In group 1, five (10%) patients were lost at follow-up: 4 patients dropped out from the study for not attending the planned visit at week 8 (the time of PCV13 booster dose administration) and one more patient refused to complete the study at week 24; one serum sample from a patient was unavailable at week 24, but it was available at 48 week. Therefore, 45 subjects in group 1 (90%) completed the follow up, and they were taken into account for the serological response analysis. All 50 (100%) patients in group 2 completed the planned follow-up, but one serum sample at week 8 and one serum sample at week 24 were unavailable.

### Antibody concentration at baseline

**Fig 2:**
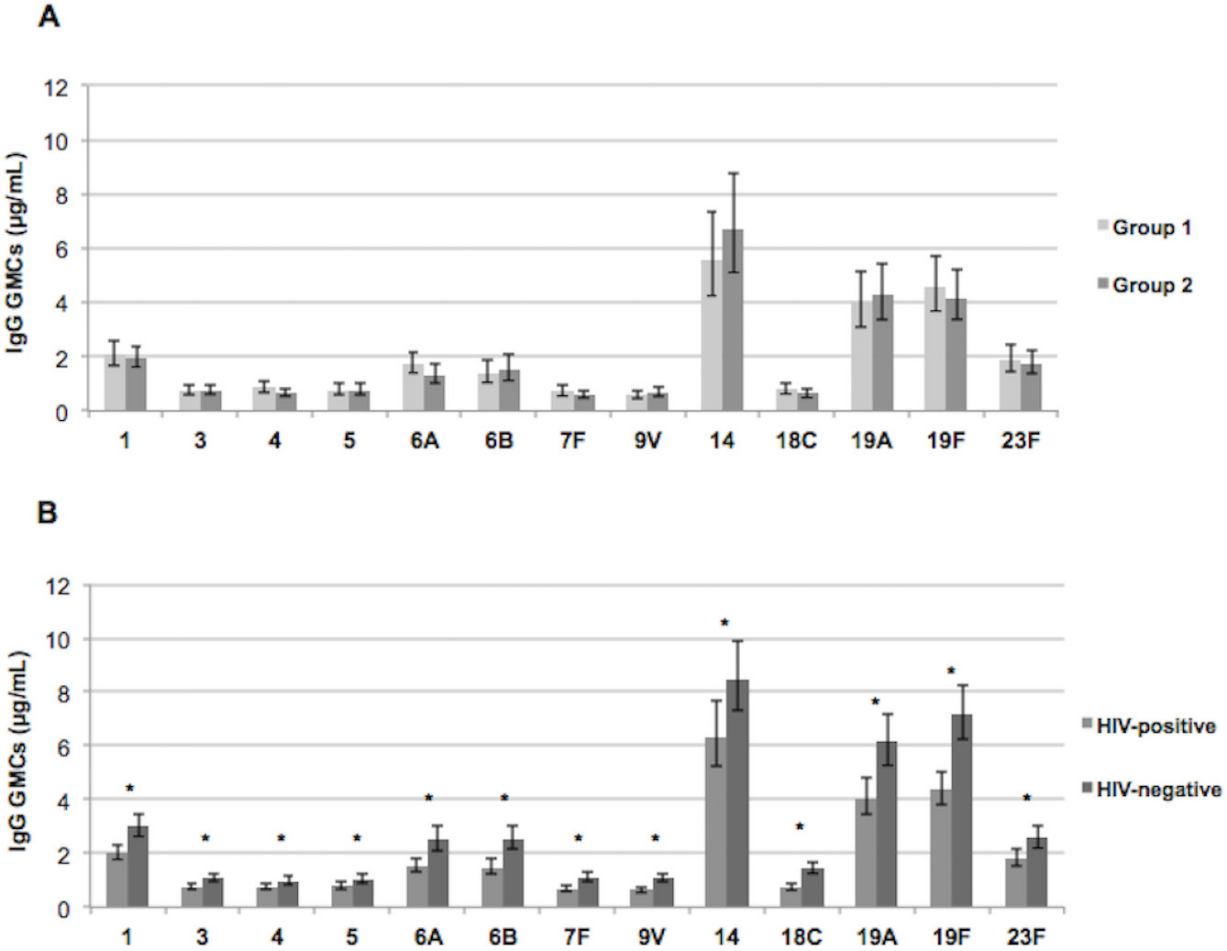
Baseline geometric mean concentrations (GMCs) of IgGs against the different PCV13 serotypes. (A) Comparison between the 2 groups of HIV-infected patients (n = 50 each group) and (B) comparison between the total population of HIV-positive subjects (n = 100) and HIV-negative controls (n = 100). Stars indicate statistically significant differences. Error bars represent the 95% confidence intervals (95% CIs).

At baseline no significant differences were observed for any PCV13 serotype-specific IgG GMC between patients who received PCV13 or PPSV23 (Fig 2A). On the other hand, the IgG GMCs against all serotypes was significantly lower in the HIV-infected patients (the two groups combined) as compared to the HIV-negative control group (Fig 2B).

By comparing the two vaccinated groups, no significant difference was observed in seroprotection rates at baseline using three different thresholds. When seroprotection levels of HIV-positive patients were compared with those of HIV-negative subjects using the ≥0.35μg/mL threshold, significantly fewer HIV-infected patients showed seroprotection levels for serotypes 5, 6B, 9V and 18C. Using the ≥1μg/mL threshold, significantly fewer HIV-infected individuals compared to HIV-negative subjects showed seroprotective levels for 9 of 13 antigens. Finally, for clinically validated serotype-specific thresholds of protection, significantly fewer HIV-infected patients compared to HIV-negative subjects showed seroprotection levels for serotypes 7F and 9V (S1 Table).

### Serological responses within groups

Both vaccines elicited an immune response after the first dose, leading to markedly higher IgG GMCs to each of the antigens compared to baseline values (with p-values of 0.007 or less). A significant fluctuation over time (“time effect”) was observed in both groups (with p-values of 0.001 or less). The only exception was represented by the low response to serotype 3 in the PCV13 group, which was modest at week 8 (vs baseline p = 1.000), and remained constant at different time points (p = 0.171) (Table 2).

**Table 2:** Geometric mean concentration (GMC) and 95% confidence intervals (CIs) of IgGs against pneumococcal serotypes included in PCV13 before (baseline) and after immunization (8, 24 and 48 weeks) with PCV13 or PPSV23 in HIV+ patients. GMC of the HIV-negative control group is also reported for comparison.

Serotype	Time point	IgG GMC, μg/mL (95% CI)						
			p	p		p	p	
		PCV13	Between first and second dose	Within-group trend over time	PPSV23	Within-group trend over time	Between PCV13 and PPSV23 after one dose	HIV-negative
**1**	**Baseline**	2.09 (1.67–2.62)			1.95 (1.62–2.34)			3.01 (2.63–3.45)
	**8 weeks**	3.30 (2.55–4.26)			4.66 (3.66–5.93)		***0*.*02***	
	**24 weeks**	2.94 (2.36–3.69)	*0*.*067*	***<0*.*001***	4.00 (3.18–5.03)	***<0*.*001***		
	**48 weeks**	2.65 (2.10–3.36)			3.29 (2.67–4.06)			
**3**	**Baseline**	0.73 (0.57–0.94)			0.76 (0.61–0.93)			1.09 (0.96–1.25)
	**8 weeks**	0.83 (0.64–1.07)			1.15 (0.94–1.41)		***0*.*033***	
	**24 weeks**	0.94 (0.75–1.18)	*0*.*625*	*0*.*171*	0.93 (0.76–1.15)	***<0*.*001***		
	**48 weeks**	0.81 (0.63–1.04)			0.91 (0.74–1.11)			
**4**	**Baseline**	0.87 (0.68–1.11)			0.68 (0.54–0.84)			0.97 (0.83–1.13)
	**8 weeks**	1.81 (1.34–2.45)			1.92 (1.49–2.47)		*0*.*725*	
	**24 weeks**	1.71(1.32–2.19)	*1*.*000*	***<0*.*001***	1,63(1.31–2.05)	***<0*.*001***		
	**48 weeks**	1.43 (1.10–1.84)			1.36 (1.11–1.67)			
**5**	**Baseline**	0.78 (0.59–1.03)			0.77 (0.57–1.03)			1.04 (0.89–1.21)
	**8 weeks**	2.51 (1.71–3.70)			2.61 (1.85–3.68)		*0*.*664*	
	**24 weeks**	2.01 (1.45–2.77)	*0*.*072*	***<0*.*001***	2.15 (1.56–2.97)	***<0*.*001***		
	**48 weeks**	1.82 (1.34–2.45)			1.65 (1.22–2.24)			
**6A**	**Baseline**	1.75 (1.40–2.18)			1.33 (1.03–1.71)			2.52 (2.09–3.04)
	**8 weeks**	4.01 (2.84–5.65)			2.18 (1.69–2.81)		***0*.*011***	
	**24 weeks**	3.24 (2.43–4.33)	***0*.*011***	***<0*.*001***	1.96 (1.53–2.52)	*0*.*095*		
	**48 weeks**	2.61 (1.99–3.42)			1.72 (1.35–2.20)			
**6B**	**Baseline**	1.41 (1.05–1.91)			1.53 (1.12–2.08)			2.54 (2.14–3.00)
	**8 weeks**	4.05 (3.00–5.48)			3.36 (2.56–4.40)		*0*.*545*	
	**24 weeks**	3.71 (2.87–4.77)	*1*.*000*	***<0*.*001***	2.69 (2.06–3.51)	***<0*.*001***		
	**48 weeks**	3.11 (2.41–3.99)			2.55 (2.00–3.25)			
**7F**	**Baseline**	0.72 (0.55–0.94)			0.60 (0.48–0.77)			1.09 (0.92–1.29)
	**8 weeks**	2.52 (1.82–3.48)			3.25 (2.35–4.50)		*0*.*222*	
	**24 weeks**	1.84 (1.39–2.44)	***0*.*024***	***<0*.*001***	2.45 (1.78–3.37)	***<0*.*001***		
	**48 weeks**	1.50 (1.13–2.00)			1.87 (1.38–2.54)			
**9V**	**Baseline**	0.57 (0.46–0.71)			0.70 (0.54–0.92)			1.09 (0.94–1.27)
	**8 weeks**	2.03 (1.46–2.81)			2.64 (1.90–3.66)		*0*.*123*	
	**24 weeks**	1.68 (1.27–2.22)	***0*.*033***	***<0*.*001***	2.28 (1.67–3.10)	***<0*.*001***		
	**48 weeks**	1.38 (1.08–1.75)			1.80 (1.33–2.44)			
**14**	**Baseline**	5.59 (4.25–7.38)			6.69 (5.11–8.77)			8.49 (7.29–9.90)
	**8 weeks**	17.25 (12.16–24.43)			25.37 (18.10–35.57)		*0*.*113*	
	**24 weeks**	15.11 (11.12–20.56)	*0*.*655*	***<0*.*001***	18.69 (13.33–26.19)	***<0*.*001***		
	**48 weeks**	12.43 (9.27–16.67)			16.33 (11.91–22.39)			
**18C**	**Baseline**	0.80 (0.63–1.02)			0.64 (0.49–0.84)			1.45 (1.25–1.67)
	**8 weeks**	4.37 (3.16–6.05)			4.04 (2.84–5.73)		*0*.*802*	
	**24 weeks**	3.35 (2.43–4.61)	***0*.*002***	***<0*.*001***	3.10 (2.22–4.33)	***<0*.*001***		
	**48 weeks**	2.50 (1.88–3.33)			2.57 (1.89–3.50)			
**19A**	**Baseline**	3.99 (3.10–5.15)			4.27 (3.36–5.43)			6.14(5.27–7.16)
	**8 weeks**	7.95 (5.97–10.57)			8.35 (6.05–11,51)		*0*.*523*	
	**24 weeks**	7.48 (5.81–9.65)	*1*.*000*	***<0*.*001***	7.22 (5.27–9.90)	***<0*.*001***		
	**48 weeks**	6.13 (4.70–7.99)			6.65 (4.87–9.08)			
**19F**	**Baseline**	4.60 (3.68–5.75)			4.18 (3.38–5.18)			7.18 (6.24–8.26)
	**8 weeks**	8.43 (6.68–10.65)			6.94 (5.39–8.94)		*0*.*355*	
	**24 weeks**	8.40 (6.78–10.41)	*1*.*000*	***<0*.*001***	6.23 (4.91–7.91)	***<0*.*001***		
	**48 weeks**	6.60 (5.23–8.34)			5.65 (4.43–7.21)			
**23F**	**Baseline**	1.86 (1.42–2.44)			1.76 (1.38–2.25)			2.58 (2.20–3.03)
	**8 weeks**	4.49 (3.24–6.24)			3.67 (2.69–5.00)		*0*.*609*	
	**24 weeks**	4.33 (3.26–5.74)	*1*.*000*	***<0*.*001***	3.19 (2.40–4.25)	***<0*.*001***		
	**48 weeks**	3.25 (2.47–4.30)			2.85 (2.18–3.72)			

Although antigen 6A was not included in the PPSV23 vaccine, it was assessed in group 2 as well; as expected no significant variation in antibody levels was observed after immunization.

Overall, in the subsequent months a common descending trend of IgG concentration was observed in both groups. In group 1, however, the immune responses against serotypes 1, 4, 5, 6B, 14, 19A, 19F and 23F did not change significantly, with IgG levels at week 24 comparable to those observed at week 8. In group 2, a similar non-significant variation in IgG levels against serotypes 1, 19A, 19F and 23F was observed. The IgG values against the remaining antigens showed a significant reduction in both groups at week 24.

At week 48, subjects vaccinated with PPSV23 showed no significant changes in IgG levels against antigens 3, 6B, 14, 19A, 19F and 23F compared to week 24; a similar finding was observed only for serotypes 3 and 5 with PCV13 in group 1, with a significant decline of IgGs against all other antigens.

**Fig 3:**
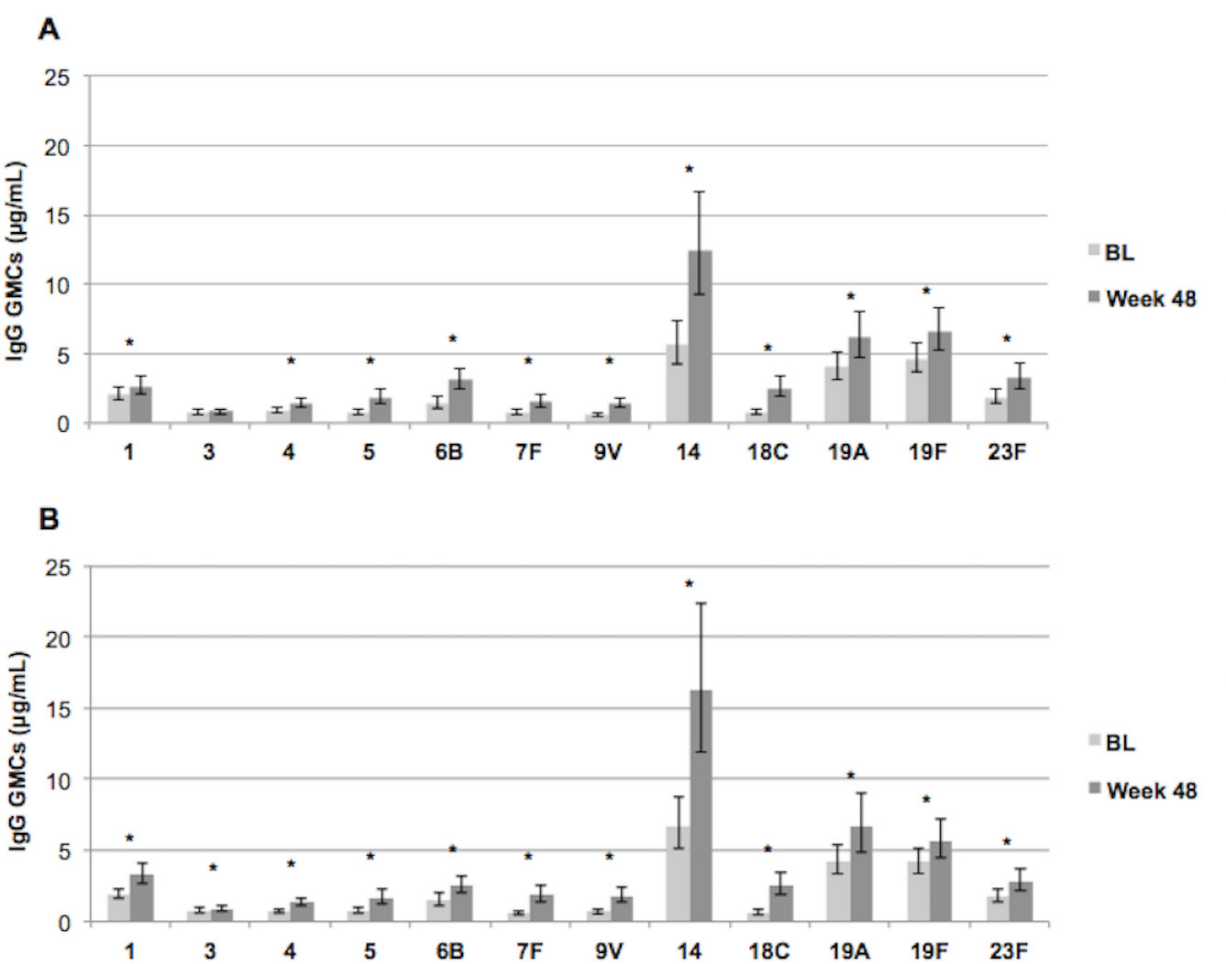
GMCs of IgGs against *S*.*pneumoniae* serotypes at baseline (BL) vs 48 weeks. (A) comparison within the PCV13 group and (B) within the PPSV23 group. Stars indicate statistically significant differences. Error bar represents the confidence intervals (CIs 95%).

Overall, at week 48 IgGs remained at significantly higher levels compared to the baseline, except for those against antigen 3 in the PCV13 group (p = 0.346) (Fig 3).

### Comparison of serological responses between groups

**Fig 4:**
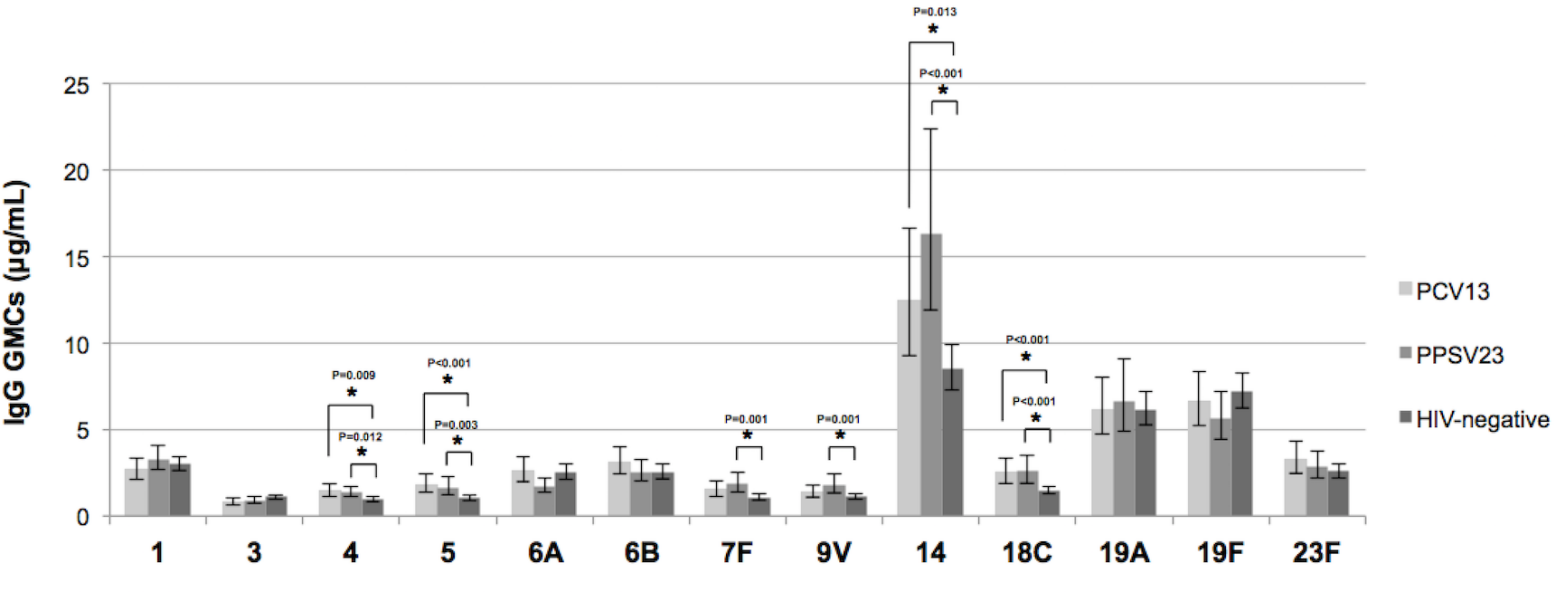
Geometric mean concentration of vaccinated groups, PCV13 and PPSV23, at 48 weeks compared to level of unvaccinated HIV-negative control group. Stars indicate significant differences between each HIV vaccinated group (PCV13 and PPSV23) and the HIV-negative unvaccinated control group. Error bar represents the confidence intervals (CIs 95%).

The overall between group analysis revealed no statistically significant difference of the IgG response to any antigen (S2 Table). The IgG titers changed over time in both groups; specifically, the IgGs against most antigens varied in a similar manner in both groups at different time points, resulting in parallel trends with no significant interaction except for IgG titers against serotypes 1 and 6B, which showed significantly divergent behavior (p = 0.007 and p = 0.038 respectively). This was because of the higher IgG titers against antigen 1 in the PPSV23 group at week 8 and the higher IgG titers against antigen 6B in the PCV13 group at week 24. At week 48, the GMCs of the IgGs against common pneumococcal antigens in the two vaccine groups were comparable. Note that for most of the antigens, GMC values in both vaccine groups did not drop below the levels observed in HIV-negative unvaccinated controls; specifically, the IgGs against antigens 4, 5, 14 and 18C were significantly higher than those observed in the HIV- negative unvaccinated group, whereas the GMCs against 9V and 7F were significantly higher than those of the non immunized subjects only in the PPSV23 group (Fig 4).

No relevant correlation emerged between the CD4 count and the immune response to different pneumococcal serotypes within and between groups. When the population was stratified by the CD4 cell count (200–350 cell/μL, 351–500 cell/μL, or >500 cell/μL) and by the plasma viral load (HIV-1 RNA >50 and ≤50 copies /mL) no relevant difference emerged.

### Seroprotection and seroconversion of IgG levels during the follow-up

**Fig 5:**
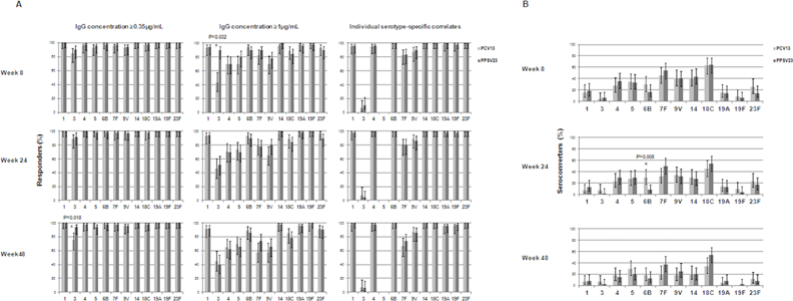
Seroprotection and seroconversion rates. (A) Proportion of responders who achieved IgG levels ≥ 0.35μg/mL and ≥1μg/mL or serotype-specific clinically validated thresholds (correlates of protection) at the different follow-up time points (a clinical correlate of protection for serotype 5 is unavailable). (B) Percentage of patients who reached IgG seroconversion defined as a ≥ 4-fold rise from baseline antibody levels at the different follow-up time points. Stars indicate a significant difference. Error bar represents the confidence intervals (CIs 95%).

After vaccination, the proportion of responders using different cut-off thresholds was similar in both groups, albeit responders to serotype 3 at weeks 8 and 48, were significantly fewer in the PCV13 group when using both thresholds of ≥1 μg/mL and of ≥ 0.35 μg/mL, respectively (Fig 5A). Percentages of seroconverters varied for each antigen in both groups; <20% of seroconverters was observed for antigens 1, 3, 19A and 19F at each time point. However, overall seroconversion rates were similar in both vaccine groups: a significant difference in seroconvertion rates was observed only for antigen 6B at week 24 when subjects immunized with PCV13 showed a significantly higher percentage of seroconvertion than those immunized with PPSV23 (Fig 5B).

### Safety

Both vaccinations were well tolerated by all subjects: no severe short-, medium- or long-term local or systemic reactions were reported.

Two patients receiving PCV13 reported mild fatigue, one immediately after injection and the other the day after, both lasting a few hours; another patient reported transient itching at the injection site. However, there was no statistical difference between the groups in terms of adverse reactions.

After the second PCV13 dose at week 8 no additional adverse events were reported.

### Clinical follow-up

No infection due to *S*. *pneumoniae* occurred during the follow-up in any of the 100 HIV-infected patients.

At 8 weeks, the proportion of individuals in the PCV13 group who had an HIV load ≤50 copies/μL was significantly lower than that of the PPSV23 patients (84.8% vs 98.0% p = 0.027). Overall, the CD4 range remained constant and comparable at the different time points between groups and no statistically significant frequency in viral blips (single measures of HIV RNA 51–500 copies/mL followed by HIV-RNA <50 copies/mL at the subsequent measure) was observed (15.6% in PCV13 vs 8.3% in PPSV23, p = 0.281).

## Discussion

While PPSV23 has been for many years a recommended pneumococcal vaccine for HIV-infected patients, PCV13 has only recently been approved for use in immunocompromised subjects [19] and data regarding its effectiveness in HIV+ adults are still scanty. Only two recent studies evaluated PCV13 in HIV+ adults and neither directly compared it with PPSV23. Glesby et al. evaluated three administrations of PCV13 in subjects previously vaccinated with PPSV23 [31] and Bhorat et al. evaluated three administrations of PCV13 followed by a single dose of PPSV23 in pediatric and adult subjects who were naïve to the pneumococcal vaccination [32].

Overall, in our population, which mainly consisted of virologically suppressed and immunologically stable HIV+ patients, both vaccines showed comparable immunogenicity at the end of the follow-up (48 weeks) and the two doses of PCV13 were as safe and well tolerated as the single dose of PPSV23. According to international guidelines, a single dose of PCV13 is now recommended in HIV+ patients aged ≥19 years who are PCV13 naïve [19]; the European Medicine Agency recommends at least 1 dose of PCV13 for immunocompromised patients and up to 4 doses for subjects with severe immunodepression (e.g. subjects with haematopoietic stem cell transplants) [33]. The administration of multiple PCV13 doses in the two aforementioned trials in HIV+ adults [31, 32] revealed modest increases in IgG GMCs after the second and third dose in contrast with the relevant increase observed after the first dose.

Similar to our study, both previous studies analyzed an HIV+ population with an overall favorable viro-immunological profile (CD4 count ≥200 cells/μL and viral load <50 copies/mL). Also, in our study no significant increase in IgGs was observed after the second dose of PCV13 (as compared to after the first dose). These current findings suggest that a single dose of PCV13 should be given in HIV-infected adults with a stable CD4 count ≥200 cells/μL and a viral load <50 copies/mL. Nonetheless, future studies should analyze patients with lower CD4 counts and should include a longer follow up in order to investigate the maintenance of IgG levels over time with single or multiple doses of PCV13.

Another relevant issue is the possible different response to different pneumococcal serotypes in relation to the single serotype itself or to the type of vaccine.

When PCV13 was compared with PPSV23 in HIV negative adults [34], most of the PCV13 serotypes elicited a statistically significantly higher response, with the exception of serotypes 3, 7F and 14. In particular, serotype 3 was reported to be less immunogenic, with the potential consequence of a lower long-term efficacy of PCV13 against pneumococcal disease caused by this serotype [35].

In our study, although no differences were observed in response to serotypes 7F and 14, PCV13 showed a lower immunogenicity for serotype 3, giving rise to a modest response at week 8; consistently, week 48 IgG levels did not differ from those at pre-vaccination. However, similar low percentages of seroconverters (<20%) were observed for serotype 3 in both study groups, similarly as for serotypes 1, 19A and 19F. Thus, substantially lower immunogenicity of these antigens can be presumed, regardless of vaccine type, in this population.

In terms of seroprotection, we found that the percentage of responders to serotype 3 was significantly lower in the PCV13 group at week 8 and 48 using the threshold of ≥1 μg/mL and of ≥0.35 μg/mL, respectively; conversely, when the serotype 3-specific clinically validated threshold was analyzed, the proportion of responders was markedly below 20% in both groups, with no significant difference. A randomized trial that compared the immunogenicity of PPSV23 with a pentavalent conjugate pneumococcal vaccine composed of serotypes 6B, 14, 18C, 19F and 23F linked to CRM197 carrier protein in HIV positive patients showed that a similar antibody response was elicited by the two vaccines [36].

In a different study, which focused on HIV-infected subjects with CD4 ≥ 200 cells/μL, the antibody concentrations between PCV7 and PPSV23 were not dramatically different for serotypes 4, 6B, 9V, 14 and 23F [37].

Our data consistently show no differences between the two vaccinated groups regarding the IgG titers of any of these antigens. A better response in terms of seroconversion against serotype 6B and 9V was previously reported in a randomized trial of HIV-infected adults vaccinated with PCV7 versus PPSV23 [17]. In our patients, the proportion of seroconverters in the PCV13 group significantly exceeded that of the PPSV23 group only for serotype 6B at week 24. On the contrary, significantly higher IgG titers against serotype 1 were observed in the PPSV23 as compared to the PCV13 group at week 8 but not at subsequent time points when the PCV13 group had received the second dose. These observations may suggest that a variably combined PCV13 and PPSV23 vaccine strategy could help overcome the low immunogenicity of some antigens in the respective vaccines.

Our study has some limitations. First of all, the small sample size. Indeed, the study was sufficiently powered only to demonstrate major differences in antibody responses between the two vaccine schedules: it was not designed as a non inferiority trial and further larger non inferiority studies will be needed. Second, the study was not randomized, although the two vaccine groups showed very similar general, immunological and exposure risk characteristics at baseline. Third, the study population showed a good and stable viro-immunological status, therefore our findings cannot be extrapolated to HIV-infected patients with more severe immune deficiency, where a different scenario is expected. Finally, a longer follow-up is required to analyze the long-term serological response, the immunological memory and their correlation with clinical events.

Nonetheless, this study represents the first direct comparison of the PCV13 and PPSV23 vaccine response in HIV-infected adults. With a one-year longitudinal follow-up study, we showed that results of PCV13 and PPSV23 comparisons obtained in HIV-negative adults [34] cannot be fully applied to the HIV-positive population.

In conclusion, we did not observe any relevant differences in immunological responses to PCV13 and PPSV23 vaccination after 1 year in HIV-infected adults. Some divergence was noted at some time points, with specific serotypes and a marked “time effect” was noted, with a progressive reduction of IgG levels over time in both vaccination groups. The property of PCV13 to elicit immunological memory could hypothetically reveal an advantage in the longer term, but this still requires demonstration in the HIV-infected population; on the other hand, the response to PPSV23 might be slightly better for certain serotypes and it obviously offers a broader serotype coverage.

Further studies are needed to evaluate long term efficacy in both groups and to establish the optimal pneumococcal vaccine strategy in HIV positive adults with different viro-immunological profiles.

## Supporting Information

S1 TREND Checklist TREND Checklist. (PDF)

S1 Table Pre-immunization seroprotective rates of IgGs against 13 *S*. *pneumoniae* antigens. (DOC)

S2 Table Comparison of the overall IgG response of PCV13 *versus* PPSV23. (DOC)

S1 Protocol Italian version. (PDF)

S2 Protocol English version. (DOC)
