# Supplementary material for: Immunogenicity and Safety of the 13-Valent Pneumococcal Conjugate Vaccine versus the 23-Valent Polysaccharide Vaccine in Unvaccinated HIV-Infected Adults: A Pilot, Prospective Controlled Study
Source: PLoS One. 2016 Jun 3;11(6):e0156523. doi: 10.1371/journal.pone.0156523 (PMC4892598; doi:10.1371/journal.pone.0156523)
Supplement: S1 Table — (DOC) [file pone.0156523.s002.doc]

**S1 TABLE**

Pre-immunization seroprotective rates of IgGs against 13 *S. pneumoniae* antigens according to two commonly used thresholds or individual serotype-specific correlates of protection: comparison between percentages of subjects from the two randomization arms who showed seroprotection values ≥0.35μg/mL (A) and ≥1μg/mL (B) and individual serotype-specific seroprotection thresholds (C); comparison between all HIV positive subjects and healthy controls who showed seroprotection levels ≥ 0.35μg/mL (D) and ≥1μg/mL (E) and individual serotype-specific seroprotection thresholds (F).

| **A** |  |  |  |  | **B** |  |  |  |  | **C** |  |  |  |  |  |  |
| --- | --- | --- | --- | --- | --- | --- | --- | --- | --- | --- | --- | --- | --- | --- | --- | --- |
|  | **Serotype** | **IgG concentration ≥0.35μg/mL (%)** | | |  | **Serotype** | **IgG concentration ≥1μg/mL (%)** | | |  | **Serotype** | **Correlate of protection μg/mL** | **PCV13 % (n=50)** | **PPSV23 % (n=50)** | ***p*** |  |
|  |  | **PCV13 n=50** | **PPSV23 n=50** | ***p*** |  |  | **PCV13 n=50** | **PPSV23 n=50** | ***p*** |  |  |  |  |  |  |  |
|  | **1** | 100 | 100 | *1.000* |  | **1** | 84 | 88 | *0.564* |  | **1** | **0.78** | 94 | 94 | *1.000* |  |
|  | **3** | 82 | 90 | *0.249* |  | **3** | 34 | 30 | *0.668* |  | **3** | **2.83** | 8 | 4 | *0.399* |  |
|  | **4** | 90 | 78 | *0.101* |  | **4** | 38 | 28 | *0.287* |  | **4** | **0.35** | 88 | 78 | *0.183* |  |
|  | **5** | 82 | 82 | *1.000* |  | **5** | 38 | 40 | *0.837* |  | **5** | **NA** |  |  | */* |  |
|  | **6A** | 98 | 98 | *1.000* |  | **6A** | 82 | 60 | *0.105* |  | **6A** | **0.16** | 100 | 100 | *1.000* |  |
|  | **6B** | 88 | 92 | *0.504* |  | **6B** | 64 | 72 | *0.391* |  | **6B** | **0.16** | 100 | 98 | *0.314* |  |
|  | **7F** | 78 | 74 | *0.639* |  | **7F** | 32 | 32 | *1.000* |  | **7F** | **0.87** | 44 | 32 | *0.216* |  |
|  | **9V** | 74 | 78 | *0.639* |  | **9V** | 24 | 34 | *0.27* |  | **9V** | **0.62** | 44 | 54 | *0.317* |  |
|  | **14** | 100 | 100 | *1.000* |  | **14** | 100 | 96 | *0.153* |  | **14** | **0.46** | 100 | 100 | *1.000* |  |
|  | **18C** | 84 | 78 | *0.444* |  | **18C** | 36 | 30 | *0.523* |  | **18C** | **0.14** | 100 | 94 | *0.078* |  |
|  | **19A** | 100 | 100 | *1.000* |  | **19A** | 92 | 98 | *0.168* |  | **19A** | **1** | 92 | 98 | *0.168* |  |
|  | **19F** | 100 | 100 | *1.000* |  | **19F** | 100 | 96 | *0.153* |  | **19F** | **1.17** | 98 | 96 | *0.557* |  |
|  | **23F** | 98 | 98 | *1.000* |  | **23F** | 72 | 74 | *0.821* |  | **23F** | **0.2** | 100 | 100 | *1.000* |  |
|  |  |  |  |  |  |  |  |  |  |  |  |  |  |  |  |  |
| **D** |  |  |  |  | **E** |  |  |  |  | **F** |  |  |  |  |  |  |
|  | **Serotype** | **IgG concentration ≥0.35μg/mL (%)** | | |  | **Serotype** | **IgG concentration ≥1μg/mL (%)** | | |  | **Serotype** | **Correlate of protection μg/mL** | **HIV-positive % (n=100)** | **HIV-negative % (n=100)** | ***p*** |  |
|  |  | **HIV-positive n=100** | **HIV-negative n=100** | ***p*** |  |  | **HIV-positive n=100** | **HIV-negative n=100** | ***p*** |  |  |  |  |  |  |  |
|  | **1** | 100 | 99 | *0.316* |  | **1** | 86 | 95 | *0.029* |  | **1** | **0.78** | 94 | 96 | *0.516* |  |
|  | **3** | 86 | 94 | *0.059* |  | **3** | 32 | 55 | *0.001* |  | **3** | **2.83** | 6 | 6 | *1.000* |  |
|  | **4** | 84 | 90 | *0.207* |  | **4** | 33 | 52 | *0.006* |  | **4** | **0.35** | 83 | 90 | *0.147* |  |
|  | **5** | 82 | 92 | *0.035* |  | **5** | 39 | 47 | *0.253* |  | **5** | **NA** | / | / | */* |  |
|  | **6A** | 98 | 99 | *0.561* |  | **6A** | 71 | 86 | *0.009* |  | **6A** | **0.16** | 100 | 100 | *1.000* |  |
|  | **6B** | 90 | 98 | *0.017* |  | **6B** | 68 | 88 | *<0.001* |  | **6B** | **0.16** | 99 | 100 | *0.316* |  |
|  | **7F** | 76 | 86 | *0.071* |  | **7F** | 32 | 54 | *0.001* |  | **7F** | **0.87** | 38 | 58 | *0.005* |  |
|  | **9V** | 76 | 93 | *<0.001* |  | **9V** | 29 | 54 | *<0.001* |  | **9V** | **0.62** | 49 | 78 | *<0.001* |  |
|  | **14** | 100 | 100 | *1.000* |  | **14** | 98 | 100 | *0.155* |  | **14** | **0.46** | 100 | 100 | *1.000* |  |
|  | **18C** | 81 | 97 | *<0.001* |  | **18C** | 33 | 70 | *<0.001* |  | **18C** | **0.14** | 97 | 100 | *0.081* |  |
|  | **19A** | 100 | 100 | *1.000* |  | **19A** | 95 | 99 | *0.097* |  | **19A** | **1** | 95 | 99 | *0.097* |  |
|  | **19F** | 100 | 100 | *1.000* |  | **19F** | 98 | 99 | *0.561* |  | **19F** | **1.17** | 97 | 99 | *0.312* |  |
|  | **23F** | 98 | 99 | *0.561* |  | **23F** | 73 | 86 | *0.022* |  | **23F** | **0.2** | 100 | 100 | *1.000* |  |
|  |  |  |  |  |  |  |  |  |  |  |  |  |  |  |  |  |
