# Supplementary material for: Immunogenicity and Safety of the 13-Valent Pneumococcal Conjugate Vaccine versus the 23-Valent Polysaccharide Vaccine in Unvaccinated HIV-Infected Adults: A Pilot, Prospective Controlled Study
Source: PLoS One. 2016 Jun 3;11(6):e0156523. doi: 10.1371/journal.pone.0156523 (PMC4892598; doi:10.1371/journal.pone.0156523)
Supplement: S2 Table — (DOC) [file pone.0156523.s003.doc]

**S2 TABLE**

Comparison of the overall IgG response of PCV13 *versus* PPSV23: *p* values determined by within-subjects and between-subjects analysis by using a “GLM- Repeated Measures ANOVA”.

| **Serotype** | **Between-arm Effect** | **Within-Arm Effect** | |
| --- | --- | --- | --- |
|  | **Vaccine** | **Time** | **Time/vaccine** |
| **1** | *0.164* | ***<0.0001*** | ***0.007**** |
| **3** | *0.400* | ***<0.0001*** | *0.050* |
| **4** | *0.637* | ***<0.0001*** | *0.202* |
| **5** | *0.999* | ***<0.0001*** | *0.577* |
| **6B** | *0.378* | ***<0.0001*** | ***0.038**** |
| **7F** | *0.414* | ***<0.0001*** | *0.052* |
| **9V** | *0.162* | ***<0.0001*** | *0.783* |
| **14** | *0.189* | ***<0.0001*** | *0.503* |
| **18C** | *0.652* | ***<0.0001*** | *0.375* |
| **19A** | *0.829* | ***<0.0001*** | *0.673* |
| **19F** | *0.227* | ***<0.0001*** | *0.197* |
| **23F** | *0.346* | ***<0.0001*** | *0.270* |

* Significant divergence was due to higher IgG titers against antigen 1 in the PPSV23 Group at week 8 and, conversely, higher IgG titers against antigen 6B in the PCV13 Group at week 24.
