# Supplementary material for: Immunogenicity and Safety of the 13-Valent Pneumococcal Conjugate Vaccine versus the 23-Valent Polysaccharide Vaccine in Unvaccinated HIV-Infected Adults: A Pilot, Prospective Controlled Study
Source: PLoS One. 2016 Jun 3;11(6):e0156523. doi: 10.1371/journal.pone.0156523 (PMC4892598; doi:10.1371/journal.pone.0156523)
Supplement: S1 Protocol — (PDF) [file pone.0156523.s004.pdf]

TITOLO DELLO STUDIO

“Risposta sierologica alla vaccinazione antipneumococcica e relativo effetto sulla colonizzazione nasale da *Streptococcus pneumoniae* in adulti HIV positivi: studio prospettico sull'efficacia del vaccino coniugato 13-valente”

EudraCT number 2011-004518-40

Codice protocollo: PCV13-HIV2011

VERSIONE DEL 14/11/11

Protocollo di studio

EudraCT number 2011-004518-40

CODICE IDENTIFICATIVO PCV13-HIV2011

Versione del 14/11/11

CONFIDENZIALE

Sperimentatore-Proponente

Dott. Francesca Montagnani
Università degli Studi di Siena
Dipartimento di Biotecnologie
Sezione di Malattie Infettive
Azienda Ospedaliera Universitaria Senese
U. O. C. Malattie Infettive Universitarie

RIASSUNTO DELLA RICERCA

Titolo dello studio: **"Risposta sierologica alla vaccinazione antipneumococcica e relativo effetto sulla colonizzazione nasale da *Streptococcus pneumoniae* in adulti HIV positivi: studio prospettico sull'efficacia del vaccino coniugato 13-valente"**

EudraCT number 2011-004518-40

Codice protocollo: PCV13-HIV2011

Prodotto in studio: Prevenar 13 sospensione iniettabile ATC: J07AL02

Vaccino pneumococcico polisaccaridico coniugato, (13-valente adsorbito); coniugato alla proteina vettrice CRM197 ed adsorbito su fosfato di alluminio (0,125 mg di alluminio)
Forma farmaceutica: sospensione iniettabile
Dosaggio: 0,5 ml, contenente 2,2 µg di polisaccaride per i sierotipi 1, 3, 4, 5, 6A, 7F, 9V, 14, 18C, 19A, 19F, 23F e 4,4 µg per il sierotipo 6B.

Prodotto di controllo: non applicabile
Forma farmaceutica prodotto di controllo: non applicabile
Dosaggio prodotto di controllo: non applicabile

Fase dello studio: III
Disegno dello studio: studio pilota, multicentrico, nazionale, di tipo non commerciale di fase III, in aperto

Razionale:
S. pneumoniae è responsabile di polmoniti, otiti medie acute, sinusiti e riacutizzazioni di bronchite cronica; un'eziologia pneumococcica è inoltre riscontrata nel 30% delle meningiti batteriche. I pazienti HIV+ risultano 10-100 volte più suscettibili alle malattie pneumococciche e soggetti ad episodi ricorrenti: *S. pneumoniae* è uno dei principali agenti di infezioni respiratorie in questa popolazione, dove rappresenta una significativa causa di morbidità e letalità.
Come intervento profilattico nei soggetti HIV+, è prevista la vaccinazione antipneumococcica con vaccino polisaccaridico 23 valente (PPV23), la cui efficacia è ben documentata nei soggetti immunocompetenti. Sebbene alcuni studi ne abbiano dimostrato l'efficacia nei pazienti HIV positivi, alcune caratteristiche della formulazione vaccinale ne mettono in discussione la validità. È da tempo dimostrato che soggetti HIV positivi con conta di CD4<500/µl sviluppano una minore risposta anticorpale post-PPV23 rispetto a soggetti sieronegativi o a pazienti HIV con minor compromissione immunologica. Inoltre nella popolazione HIV positiva, PPV23 non è in grado di indurre una risposta duratura, che non essendo legata ad una stimolazione antigenica T-dipendente, non risente neppure di effetto booster dopo rivaccinazione.
Poiché i dati sull'efficacia clinica del PPV23 non sono ancora chiari, particolarmente nella popolazione HIV positiva, appaiono necessarie ulteriori valutazioni sulle strategie vaccinali. Rispetto ai vaccini polisaccaridici, i vaccini coniugati (PCV) con carrier proteico incrementano la risposta anticorpale per induzione di risposta immune T-dipendente e la formazione di cellule B e T di memoria.
Un vaccino antipneumococcico eptavalente coniugato con il tossoide difterico CRM197 (PCV-7) è approvato in Europa dal 2001 e si è dimostrato efficace nel ridurre l'incidenza di malattie invasive da sierotipi vaccinali (4, 6B, 9V, 14, 18C, 19F, 23F), sia nei bambini che negli adulti, per effetto dell'immunità di gregge.
L'emergenza di sierotipi non vaccinali come colonizzanti e come causa di patologie invasive ha portato allo sviluppo di un'ulteriore formulazione 13-valente (PCV-13), che utilizza la stessa proteina carrier CRM197 e che copre i sette sierotipi del PCV-7 più i sierotipi 1, 3, 5, 6A, 7F e 19A. L'efficacia e la sicurezza del PCV-13 sono state dimostrate per la popolazione pediatrica, per cui la formulazione ha specifica indicazione. La commercializzazione del PCV-13, sulla base del parere favorevole dell'EMEA del settembre 2009, è stata autorizzata in tutti i Paesi Europei. Tale formulazione è stata approvata dall'AIFA per impiego in età pediatrica, in sostituzione di PCV7. Nella nuova proposta di calendario vaccinale italiano è inserito il vaccino PCV-13 e, in attesa di poter disporre di vaccini pneumococcici coniugati registrati anche per l'utilizzo nella popolazione adulta, la Società Italiana di Igiene, Medicina Preventiva e Sanità Pubblica raccomanda, previo ottenimento di consenso informato, l'utilizzo di una dose iniziale di vaccino pneumococcico coniugato 13-valente seguito a distanza di almeno 2 mesi dalla somministrazione di vaccino polisaccaridico 23-valente.

La capacità dei vaccini coniugati di indurre una risposta immunitaria di memoria crea i presupposti per una possibile maggiore efficacia nei soggetti immunodepressi. Studi sulla popolazione adulta HIV positiva hanno riportato risultati contrastanti sulla superiorità in termini di efficacia del PCV-7 rispetto al PPV23. In alcuni casi è stata evidenziata una buona protezione nei confronti di malattie invasive in adulti HIV positivi dopo somministrazione PCV-7 ed una migliore risposta anticorpale quantitativa e qualitativa nell'impiego di PCV-7 da solo o in combinazione con PPV23. Risultati contrari sono emersi da differenti analisi ed in particolare la possibile induzione di tolleranza immunitaria da parte di PPV23, potrebbe inficiare ulteriormente l'efficacia profilattica nella popolazione HIV positiva già poco responsiva alla stimolazione polisaccaridica.
L'utilizzo del PCV-13 in soggetti adulti nei primi trial in fase 1 ha dimostrato un profilo di efficacia uguale o superiore in termini di risposta anticorpale rispetto a PPV23, con un profilo di tollerabilità sovrapponibile a PCV-7. Con tali presupposti, il maggior numero di sierotipi contenuti nel PCV-13 rispetto a PCV-7 rendono molto promettente l'impiego della formulazione 13-valente nella popolazione adulta HIV positiva, in termini di efficacia nella prevenzione delle malattie pneumococciche invasive. Nella popolazione HIV, inoltre, un successivo booster di PCV-13 potrebbe aumentare la risposta anticorpale e quindi l'efficacia profilattica.
Lo studio della risposta sierologica a tale schema vaccinale, associato all'analisi della colonizzazione nasofaringea e del seguente sviluppo di patologia, può fornire un basilare supporto per la valutazione di tale strategia profilattica.

Il progetto si propone di valutare, in modo prospettico, la risposta anticorpale a 2 dosi di PCV-13 e la prevalenza della colonizzazione nasofaringea da *S. pneumoniae* in una popolazione HIV+ non ospedalizzata, correlando i dati anamnestici, clinici, sierologici e microbiologici.
In assenza di controindicazioni, 50 soggetti HIV positivi saranno sottoposti al T0 a vaccinazione con PCV13, monitorizzando gli effetti avversi a breve (30 minuti), medio (<= 5 giorni) e lungo termine, con valutazione clinica nell'immediato post-vaccinale, intervista telefonica al 5° giorno post-vaccinale e raccordo clinico-anamnestico durante il successivo follow up (4, 8, 24 e 48 settimane).
Uno screening microbiologico (tampone nasofaringeo per colonizzazione da *S. pneumoniae*), sierologico (prelievo ematico per studio anticorpale) e clinico (anamnesi ed esame obiettivo) sarà effettuato al T0 e a 4, 8, 24 e 48 settimane.
Ad 8 settimane verrà somministrata una dose booster di PCV-13, monitorizzando gli effetti avversi a breve (30 minuti), medio (<= 5 giorni) e lungo termine come precedentemente descritto per la prima dose.

End-point primario: determinare la risposta anticorpale indotta da vaccinazione primaria con vaccino coniugato 13-valente e successiva dose booster ad 8 settimane in adulti HIV+;

End-point secondario: Valutare l'entità della colonizzazione da differenti sierotipi di *S. pneumoniae* in soggetti HIV positivi, in relazione al livello anticorpale al T0; definire l'effetto delle due dosi di PCV13 sulla colonizzazione nasofaringeo e sull'insorgenza di infezioni pneumococciche invasive in adulti HIV+; valutare la chemiosensibilità degli isolati ai differenti antibiotici e stabilire la percentuale di isolati multiresistenti; valutare l'epidemiologia molecolare degli pneumococchi isolati.

Numero pazienti da arruolare: Nello studio saranno inclusi 50 pazienti adulti (età > 18 anni) con infezione da HIV

Numero Centri Sperimentali: 2

Numero pazienti da arruolare per ogni centro: max 10 pazienti (Siena) e 40 pazienti (Roma)

Durata presumibile dello studio: 24 mesi

PRIMA FASE: 12 mesi (1 Novembre 2011 – 1 Novembre 2012) arruolamento + vaccinazione, screening microbiologico e sierologico;

SECONDA FASE: 12 mesi (1 Novembre 2012 – 31 Ottobre 2013) completamento studio sierologico, microbiologico ed analisi dei dati.

Criteri di inclusione
- età > 18 anni
- disponibilità dal parte del paziente o del tutore legale a fornire il proprio consenso libero ed informato
- accesso alle strutture in regime ambulatoriale o di Day Hospital
- CD4 ≥ 200 cell/µl in due determinazioni consecutive precœdenti al T0

Criteri di esclusione
- età >65 anni
- patologia infettiva acuta in atto
- antibiosi in atto o pregressa <= 7 giorni
- pregressa vaccinazione con PPV23 o con PCV7
- gravidanza
- terapia immunomodulante in atto
- immunodepressione non HIV relata

INDICE

1. INFORMAZIONI DI CARATTERE GENERALE pagina ..

2. INTRODUZIONE ..
   2.1 Informazioni di base e razionale ..
   2.2 Descrizione del farmaco ..

3. OBIETTIVI E FINALITA' DELLO STUDIO ..

4. DISEGNO SPERIMENTALE ..
   4.1 Endpoints dello Studio ..
   4.2 Piano Sperimentale ..
   4.3 Diagramma dello studio ..

5. POPOLAZIONE IN STUDIO ..
   5.1 Dimensione del campione ..
   5.2 Parametri d'identificazione della popolazione in studio ..
   5.3 Selezione dei pazienti ..
      5.3.1 Criteri di inclusione ..
      5.3.2 Criteri di esclusione ..
   5.4 Criteri di conclusione del periodo di osservazione ..

6. TRATTAMENTO DEI SOGGETTI ..
   6.1 Trattamento in studio ..
      6.1.1 Dosaggio, Posologia e via di somministrazione ..
      6.1.2 Speciali avvertenze e precauzioni d'uso ..
      6.1.3 Confezionamento ed etichettatura del farmaco ..
      6.1.4 Drug Accountability ..
   6.2 Randomizzazione ..
   6.3 Terapie Farmacologiche Concomitanti ..
   6.4 Compliance ..
   6.5 Sospensione/Interruzione del trattamento ..

7. VALUTAZIONE DELL'EFFICACIA ..
8. VALUTAZIONE DELLA SICUREZZA E TOLLERABILITA' ..
   8.1 Eventi avversi ..
      8.1.1 Definizioni ..

9. ACCESSO DIRETTO AI DOCUMENTI ORIGINALI ..

10. PROCEDURE DI CONTROLLO E DI ASSICURAZIONE DELLA QUALITA' … ..
    10.1 Scheda Raccolta Dati (SRD) ..
    10.2 Audits ..
    10.3 Ispezioni ..

11. GESTIONE DEI DATI CLINICI ..

12. ANALISI STATISTICA ..

13. ASPETTI ETICI ..
    13.1 Autorizzazioni Etiche ..
    13.2 Consenso Informato ..

14. PROCEDURE AMMINISTRATIVE ..
    14.1 Cambiamenti nella condotta dello studio o analisi pianificate ..
    14.2 Sospensione/Interruzione dello studio ..
    14.3 Archiviazione ..
    14.4 Confidenzialità e pubblicazione delle informazioni ..
    14.5 Copertura assicurativa di responsabilità civile ..
    14.6 Finanziamento della sperimentazione ..
15. RESPONSABILITA' DELLO SPERIMENTATORE ..

16. RAPPORTO FINALE DELLO STUDIO ..

17. FASI E TEMPISTICA ..

18. BIBLIOGRAFIA ..

APPENDICE 1 - Dichiarazione di Helsinki ..

ABBREVIAZIONI CONTENUTE NEL TESTO

PCV7	Vaccino antipneumococcico coniugato eptavalente
PCV13	Vaccino antipneumococcico coniugato 13-valente
PPV23	Vaccino antipneumococcico polisaccaridico 23-valente
CDC	Center for Diseases Control
PNSSP	*Streptococcus pneumoniae* con ridotta sensibilità a penicillina
PRSP	*Streptococcus pneumoniae* resistente a penicillina
HAART	Highly Active Antiretroviral Therapy
IPD	Invasive pneumococcical disease
OPA	Titoli anticorpali funzionali
GMT	Medie geometriche dei titoli funzionali
OMA	Otite media acuta
IDUs	Illicit drug users

Firma di Approvazione del protocollo

Dott. Francesca Montagnani ……………………………………..……………………
…………..……………………

Sperimentatore-Proponente
Università degli Studi di Siena
Dipartimento di Biotecnologie
Sezione di Malattie Infettive
Azienda Ospedaliera Universitaria Senese
U. O. C. Malattie Infettive Universitarie

1. INFORMAZIONI DI CARATTERE GENERALE

Titolo dello studio: **"Risposta sierologica alla vaccinazione antipneumococcica e relativo effetto sulla colonizzazione nasale da *Streptococcus pneumoniae* in adulti HIV positivi: studio prospettico sull'efficacia del vaccino coniugato 13-valente" - EudraCT number 2011-004518-40 - Codice protocollo: PCV13-HIV2011**

"Serological response to antipneumococcal vaccination and consequent impact on *Streptococcus pneumoniae* nasal carriage in HIV positive adults: a prospective study using 13-valent conjugate vaccine."

Prodotto in studio: Prevenar 13 sospensione iniettabile ATC: J07AL02

Vaccino pneumococcico polisaccaridico coniugato, (13-valente adsorbito); coniugato alla proteina vettrice CRM197 ed adsorbito su fosfato di alluminio (0,125 mg di alluminio).

Forma farmaceutica: sospensione iniettabile

Dosaggio: 0,5 ml, contenente 2,2 µg di polisaccaride per i sierotipi 1, 3, 4, 5, 6A, 7F, 9V, 14, 18C, 19A, 19F, 23F e 4,4 µg per il sierotipo 6B.

Prodotto di controllo: non applicabile
Forma farmaceutica prodotto di controllo: non applicabile
Dosaggio prodotto di controllo: non applicabile

Fase dello studio: III

Versione e data: Versione 1.0 01-09-11

Prodotto: Prevenar 13 sospensione iniettabile ATC: J07AL02

Sperimentatore-Proponente: Dott. Francesca Montagnani

Indirizzo: UOC Malattie Infettive Universitarie, IV lotto piano 0, Policlinico Le Scotte, viale Bracci, 16, 53100 Siena

Telefono: 0577-586533/62

Fax: 0577233462

2. INTRODUZIONE

2.1 Informazioni di base e razionale

S. pneumoniae è responsabile di polmoniti, otiti medie acute, sinusiti e riacutizzazioni di bronchite cronica; un'eziologia pneumococcica è inoltre riscontrata nel 30% delle meningiti batteriche. I pazienti HIV+ risultano 10-100 volte più suscettibili alle malattie pneumococciche e soggetti ad episodi ricorrenti: *S. pneumoniae* è uno dei principali agenti di infezioni respiratorie in questa popolazione, dove rappresenta una significativa causa di morbidità e letalità.
Come intervento profilattico nei soggetti HIV+, è prevista la vaccinazione antipneumococcica con vaccino polisaccaridico 23 valente (PPV23), la cui efficacia è ben documentata nei soggetti immunocompetenti. Sebbene alcuni studi ne abbiano dimostrato l'efficacia nei pazienti HIV positivi, alcune caratteristiche della formulazione vaccinale ne mettono in discussione la validità. È da tempo dimostrato che soggetti HIV positivi con conta di CD4<500/µl sviluppano una minore risposta anticorpale post-PPV23 rispetto a soggetti sieronegativi o a pazienti HIV con minor compromissione immunologica. Inoltre nella popolazione HIV positiva, PPV23 non è in grado di indurre una risposta duratura, che non essendo legata ad una stimolazione antigenica T-dipendente, non risente neppure di effetto booster dopo rivaccinazione.
Poiché i dati sull'efficacia clinica del PPV23 non sono ancora chiari, particolarmente nella popolazione HIV positiva, appaiono necessarie ulteriori valutazioni sulle strategie vaccinali. Rispetto ai vaccini polisaccaridici, i vaccini coniugati (PCV) con carrier proteico incrementano la risposta anticorpale per induzione di risposta immune T-dipendente e la formazione di cellule B e T di memoria.
Un vaccino antipneumococcico eptavalente coniugato con il tossoide difterico CRM197 (PCV-7) è approvato in Europa dal 2001 e si è dimostrato efficace nel ridurre l'incidenza di malattie invasive da sierotipi vaccinali (4, 6B, 9V, 14, 18C, 19F, 23F), sia nei bambini che negli adulti, per effetto dell'immunità di gregge.
L'emergenza di sierotipi non vaccinali come colonizzanti e come causa di patologie invasive ha portato allo sviluppo di un'ulteriore formulazione 13-valente (PCV-13), che utilizza la stessa proteina carrier CRM197 e che copre i sette sierotipi del PCV-7 più i sierotipi 1, 3, 5, 6A, 7F e 19A. L'efficacia e la sicurezza del PCV-13 sono state dimostrate per la popolazione pediatrica, per cui la formulazione ha specifica indicazione. La commercializzazione del PCV-13, sulla base del parere favorevole dell'EMEA del settembre 2009, è stata autorizzata in tutti i Paesi Europei. Tale formulazione è stata approvata dall'AIFA per impiego in età pediatrica, in sostituzione di PCV7. Nella nuova proposta di calendario vaccinale italiano è inserito il vaccino PCV-13 e, in attesa di poter disporre di vaccini pneumococcici coniugati registrati anche per l'utilizzo nella popolazione adulta, la Società Italiana di Igiene, Medicina Preventiva e Sanità Pubblica raccomanda l'utilizzo di una dose iniziale di vaccino pneumococcico coniugato 13-valente seguito a distanza di almeno 2 mesi dalla somministrazione di vaccino polisaccaridico 23-valente.
La capacità dei vaccini coniugati di indurre una risposta immunitaria di memoria crea i presupposti per una possibile maggiore efficacia nei soggetti immunodepressi. Studi sulla popolazione adulta HIV positiva hanno riportato risultati contrastanti sulla superiorità in termini di efficacia del PCV-7 rispetto al PPV23. In alcuni casi è stata evidenziata una buona protezione nei confronti di malattie invasive in adulti HIV positivi dopo somministrazione PCV-7 ed una migliore risposta anticorpale quantitativa e qualitativa nell'impiego di PCV-7 da solo o in combinazione con PPV23. Risultati contrari sono emersi da differenti analisi ed in particolare la possibile induzione di tolleranza immunitaria da parte di PPV23, potrebbe inficiare ulteriormente l'efficacia profilattica nella popolazione HIV positiva già poco responsiva alla stimolazione polisaccaridica.
L'utilizzo del PCV-13 in soggetti adulti nei primi trial in fase 1 ha dimostrato un profilo di efficacia uguale o superiore in termini di risposta anticorpale rispetto a PPV23, con un profilo di tollerabilità sovrapponibile a PCV-7. Con tali presupposti, il maggior numero di sierotipi contenuti nel PCV-13 rispetto a PCV-7 rendono molto promettente l'impiego della formulazione 13-valente nella popolazione adulta HIV positiva, in termini di efficacia nella prevenzione delle malattie pneumococciche invasive. Nella popolazione HIV, inoltre, un successivo booster di PCV-13 potrebbe aumentare la risposta anticorpale e quindi l'efficacia profilattica.
Lo studio della risposta sierologica a tale schema vaccinale, associato all'analisi della colonizzazione nasofaringea e del seguente sviluppo di patologia, può fornire un basilare supporto per la valutazione di tale strategia profilattica.

2.2 Descrizione del farmaco

Proprietà farmacodinamiche

Categoria farmacoterapeutica: vaccini pneumococcici; codice ATC: J07AL02.
Prevenar 13 contiene i 7 polisaccaridi capsulari pneumococccici presenti in Prevenar (4, 6B, 9V, 14, 18C, 19F, 23F) più 6 polisaccaridi supplementari (1, 3, 5, 6A, 7F, 19A), tutti coniugati alla proteina vettrice CRM197 .
In base alla sorveglianza dei sierotipi in Europa, condotta prima dell'introduzione di Prevenar, è stato stimato che Prevenar 13 copra il 73-100% (a seconda del paese) dei sierotipi che causano la patologia invasiva da pneumococco (IPD) in bambini di età inferiore a 5 anni. In questa fascia di età, i sierotipi 1, 3, 5, 6A, 7F e 19A sono responsabili del 15,6%-59,7% della malattia invasiva, a seconda del paese, del periodo studiato e dell'utilizzo di Prevenar.
L'otite media acuta (OMA) è una malattia infantile comune con differenti eziologie. I batteri possono essere responsabili del 60-70% degli episodi clinici di OMA. Lo *S. pneumoniae* è in tutto il mondo una delle cause più comuni di OMA batterica.

Si stima che Prevenar 13 copra oltre il 90% dei sierotipi che causano la patologia pneumococcica invasiva antibiotico-resistente.

Studi Clinici di Immunogenicità con Prevenar 13.

L'efficacia protettiva di Prevenar 13 contro le IPD non è stata analizzata. Come raccomandato dall'Organizzazione Mondiale della Sanità (OMS), la valutazione dell'efficacia potenziale contro le IPD è stata basata su un confronto delle risposte immunitarie ai sette sierotipi comuni a Prevenar 13 e Prevenar, la cui efficacia protettiva è stata dimostrata. Sono state valutate anche le risposte immunitarie ai 6 sierotipi aggiuntivi.

Risposta immunitaria a seguito della serie primaria infantile a tre dosi

Gli studi clinici sono stati condotti in diversi paesi europei e negli Stati Uniti con una serie di programmi di vaccinazione, comprendenti due studi randomizzati di non inferiorità (in Germania con una serie primaria a 2, 3, 4 mesi [006] e negli Stati Uniti con una serie primaria a 2, 4, 6 mesi [004]). In questi due studi sono state confrontate le risposte immunitarie pneumococciche utilizzando una serie di criteri di non inferiorità, tra cui la percentuale di soggetti che un mese dopo la serie primaria presentava IgG sieriche anti-polisaccaridiche sierotipo-specifiche ≥ 0,35 µg/ml ed il confronto delle concentrazioni medie geometriche delle IgG (ELISA GMC's); inoltre, sono stati confrontati i titoli anticorpali funzionali (OPA) tra i soggetti che ricevevano Prevenar 13 e Prevenar. Per i sei sierotipi aggiuntivi, questi valori sono stati confrontati con la risposta più bassa tra tutti i 7 sierotipi comuni in coloro che avevano ricevuto Prevenar. I confronti sulla non-inferiorità di risposta immunitaria nello studio 006, basati sulla proporzione di neonati che raggiungono una concentrazione di anti-polisaccaride IgG ≥ 0,35 µg/ml, sono mostrati nella Tabella 1. I risultati dello studio 004 sono stati simili. La non inferiorità di Prevenar 13 (limite inferiore del 95% IC per la differenza in percentuale, tra i soggetti dei due gruppi che rispondono a 0,35 µg/ml, superiore a -10%) è stata dimostrata per tutti i 7 sierotipi comuni, ad eccezione del sierotipo 6B nello studio 006 e dei sierotipi 6B e 9V nello studio 004, i quali hanno mancato di poco il margine. Tutti i 7 sierotipi comuni hanno rispettato i criteri pre-stabiliti di non inferiorità per le IgG ELISA GMC's. Prevenar 13 ha elicitato per i 7 sierotipi comuni livelli di anticorpi paragonabili, benché leggermente più bassi, a quelli di Prevenar. La rilevanza clinica di queste differenze non è nota. La non inferiorità è stata dimostrata nello studio 006 per i 6 sierotipi aggiuntivi, sulla base del numero di neonati che raggiungono una concentrazione di anticorpi ≥ 0,35 µg/ml e nel confronto delle IgG ELISA GMCs, ed è stata dimostrata per 5 dei 6 sierotipi, con l'eccezione del sierotipo 3 nello studio 004. Per il sierotipo 3, la percentuale di Prevenar 13 con IgG sieriche ≥ 0,35 µg/ml era pari al 98,2 % (studio 006) ed al 63,5% (studio 004).

Tabella 1: Confronto della proporzione dei soggetti che raggiungono una concentrazione di anticorpi antipolisaccaridici pneumococcici IgG ≥ 0.35 µg/ml dopo la terza dose della serie infantile – Studio 006

Sierotipi	Prevenar 13 % (N=282-285)	Prevenar 7valente % (N=277-279)	Differenza (95 % CI)
**Sierotipi di Prevenar 7valente**			
4	98.2	98.2	0.0 (-2.5, 2.6)
6B	77.5	87.1	-9.6 (-16.0, -3.3)
9V	98.6	96.4	2.2 (-0.4, 5.2)
14	98.9	97.5	1.5 (-0.9, 4.1)
18C	97.2	98.6	-1.4 (-4.2, 1.2)
19F	95.8	96.0	-0.3 (-3.8, 3.3)
23F	88.7	89.5	-0.8 (-6.0, 4.5)
**Sierotipi aggiuntivi di Prevenar 13**			
1	96.1	87.1*	9.1 (4.5, 13.9)
3	98.2	87.1	11.2 (7.0, 15.8)
5	93.0	87.1	5.9 (0.8, 11.1)
6A	91.9	87.1	4.8 (-0.3, 10.1)
7F	98.6	87.1	11.5 (7.4, 16.1)
19A	99.3	87.1	12.2 (8.3, 16.8)

*In Prevenar il sierotipo 6B ha mostrato il più basso grado di risposta percentuale nello studio 006 (87.1 %)

Prevenar 13 negli studi 004 e 006 ha elicitato anticorpi funzionali per tutti i 13 sierotipi contenuti nel vaccino. Per i 7 sierotipi comuni non vi erano differenze nelle percentuali tra i soggetti con titoli OPA ≥ 1:8. Per ciascuno dei sette sierotipi comuni, più del 96% e più del 90% di coloro che avevano ricevuto Prevenar 13, un mese dopo la serie primaria,

hanno raggiunto un titolo OPA ≥ 1:8, negli studi 006 e 004 rispettivamente. Per ciascuno dei 6 sierotipi aggiuntivi, Prevenar 13 ha elicitato titoli OPA ≥ 1:8, dal 91,4% al 100% dei vaccinati, un mese dopo la serie primaria, negli studi 004/006. I titoli medi geometrici di anticorpi funzionali OPA per i sierotipi 1,3 e 5 erano più bassi dei titoli per ciascuno degli altri sierotipi addizionali; la rilevanza clinica di questa osservazione ai fini dell'efficacia protettiva non è nota.

Risposta immunitaria a seguito della serie primaria a due dosi.

L'immunogenicità dopo due dosi, nei neonati, è stata dimostrata in quattro studi. La percentuale di bambini che ha raggiunto una concentrazione di polisaccaride pneumococcico anticapsulare IgG ≥ 0,35 mg/ml, dopo un mese dalla seconda dose, oscillava tra il 79,6% ed il 98,5% in 11 dei 13 sierotipi presenti nel vaccino. Percentuali più basse di bambini hanno raggiunto la soglia di questa concentrazione anticorpale per i sierotipi 6B (tra il 27,9% ed il 57,3%) e 23F (tra il 55,8% ed il 68,1%) in tutti gli studi con somministrazione a 2 e 4 mesi, paragonate al 58,4% per il sierotipo 6B ed al 68,6% per il sierotipo 23F in uno studio con somministrazione a 3 e 5 mesi. Dopo la dose di richiamo tutti i sierotipi del vaccino, inclusi il 6B ed il 23F, hanno avuto una risposta immunitaria compatibile con una stimolazione adeguata con una serie primaria a due dosi. In uno studio in UK le risposte funzionali degli anticorpi (OPA) sono risultate paragonabili per tutti i sierotipi, inclusi il 6B ed il 23F nei gruppi di Prevenar e Prevenar 13 dopo la serie primaria a due e quattro mesi d'età e dopo la dose di richiamo a 12
mesi di età. Per coloro che hanno ricevuto Prevenar 13 la percentuale di coloro che hanno risposto con titolo OPA ≥ 1:8 è stata di almeno l'87% dopo la serie primaria e di almeno il 97% dopo la dose di richiamo. I titoli medi geometrici OPA per i sierotipi 1,3 e 5 erano più bassi di quelli di ognuno degli altri sierotipi addizionali; la rilevanza clinica di tale osservazione è sconosciuta.

Risposte dopo la dose di richiamo a seguito delle serie primarie a due dosi ed a tre dosi.

Per tutti i 13 sierotipi, dopo la dose di richiamo, la concentrazione anticorpale è aumentata rispetto al livello precedente il richiamo stesso. Per 12 sierotipi le concentrazioni anticorpali che dopo la dose di richiamo sono state più alte rispetto a quelle raggiunte dopo la serie primaria infantile. Queste osservazioni sono coerenti con una stimolazione adeguata (induzione di una memoria immunologia). La risposta immunitaria dopo la dose di richiamo per il sierotipo 3, non è aumentata oltre i livelli osservati dopo la serie di vaccinazione infantile; la rilevanza clinica di questa osservazione riguardo l'induzione della memoria immunitaria per il sierotipo 3 non è nota. Le risposte anticorpali in seguito alla dose di richiamo dopo la serie primaria infantile, sia di due che di tre dosi, sono state paragonabili a quelle raggiunte per tutti i 13 sierotipi del vaccino. Per i bambini di età compresa tra i 7 mesi ed i 5 anni un'appropriata schedula vaccinale di recupero porta a livelli di risposta IgG anti-capsulare polisaccaridico per ognuno dei 13 sierotipi, almeno paragonabili a quelli della serie primaria a tre dosi nei bambini. La persistenza a lungo termine degli anticorpi non è stata studiata dopo somministrazione di Prevenar 13, né per la serie primaria nei neonati con dose di richiamo né dopo la somministrazione di una dose singola d'immunizzazione nei bambini più grandi. Dall'introduzione di Prevenar 7valente, nel 2000, i dati sulla sorveglianza della malattia pneumococcica non hanno mostrato che l'immunità elicitata da Prevenar nei bambini sia diminuita nel tempo.

Risposta immunitaria a seguito della somministrazione sottocutanea

La somministrazione sottocutanea di Prevenar 13 è stata valutata in uno studio non comparativo in 185 neonati e bambini giapponesi sani che hanno ricevuto quattro dosi a 2, 4, 6 e 12-15 mesi di età. Lo studio ha dimostrato che sicurezza e immunogenicità erano generalmente paragonabili alle osservazioni fatte negli studi di somministrazione intramuscolare.

Efficacia protettiva di Prevenar (vaccino 7valente)

L'efficacia di Prevenar 7valente è stata valutata in due studi maggiori – lo studio Northern California Kaiser Permanente (NCKP) e lo studio Finnish Otitis Media (FinOM). Entrambi gli studi erano randomizzati, in doppio cieco, con controllo attivo, nei quali i bambini erano randomizzati per ricevere Prevenar o il vaccino di controllo (NCKP, vaccino meningococcico sierogruppo C CRM-coniugato [MnCC]; FinOM, vaccino epatite B) in una serie di quattro dosi a 2, 4, 6 e 12-15 mesi d'età. I risultati di efficacia ottenuti da tali studi (per la malattia pneumococcica invasiva, la polmonite e l'otite media acuta) sono elencati qui di seguito (tabella 2).

Table 2: Riassunto dell'efficacia di Prevenar 7valente[1]

Test	N	VE[2]	95% CI
NCKP: IPD da sierotipo del vaccino [3]	30,258	97%	85, 100
NCKP: Polmonite clinica con radiografia toracica anormale	23,746	35%	4,56
NCKP: Otite Media Acuta (OMA)[4]	23,746		
Episodi Totali		7%	4,10
OMA Recidività (3 episodi in 6 mesi, o 4 episodi in 1 anno)		9%	3,15
OMA Recidività (5 episodi in 6 mesi, o 6 episodi in 1 anno)		23%	7,36
Posizionamento di tubo timpanostomico		20. %	2,35
FinOM: AOM	1,662		
Episodi totali		6%	-4, 16
Tutte le OMA pneumococciche		34%	21, 45
OMA da sierotipo del vaccino		57%	44, 67

[1]Per protocollo
[2]Efficacia del vaccino
[3]Ottobre 1995-20 Aprile 1999
[4]Ottobre 1995-30 Aprile 1998

L'efficacia protettiva di Prevenar (vaccino 7valente)

L'efficacia (effetto diretti e indiretto) di Prevenar 7valente contro le malattie da pneumococco è stata valutata durante i programmi di immunizzazione nella serie primaria infantile sia a tre-dosi che a due-dosi, ciascuna con dose di richiamo (Tabella 3). In seguito all'uso diffuso di Prevenar, l'incidenza di IPD è stata coerentemente e sostanzialmente ridotta. Un aumento nell'incidenza dei casi di IPD causata da sierotipi non contenuti in Prevenar, come 1, 7F e 19A, è stato riportato in alcuni paesi. La sorveglianza continuerà con Prevenar 13, e poiché i paesi aggiornano i propri dati di sorveglianza, le informazioni in questa tabella possono cambiare.
Usando il metodo di screening, l'efficacia specifica per sierotipo valutata per 2 dosi somministrate al di sotto dell'anno di età nel Regno Unito era pari al 66% (-29, 91 %) e al 100% (25, 100%) per i sierotipi 6B e 23F, rispettivamente.

Tabella 3: Riassunto di efficacia di Prevenar 7valente per la patologia invasiva pneumococcica

Paese (anno di introduzione)	Proramma d'immunizzazione raccomandato	Riduzione della patologia, %	95% CI
Regno Unito (Inghilterra & Galles)[1] (2006)	2, 4, + 13 mesi	Sierotipi del vaccino:<br>Due dosi al di sotto di un anno di età: 85%	49, 95%
USA (2000)	2, 4, 6, +12 - 15 mesi		
Bambini < 5[2]		Sierotipi del vaccino: 98%<br>Tutti i sierotipi: 77%	97, 99%<br>73, 79%
Adulti ≥ 65[3]		Sierotipi del vaccino: 76%<br>Tutti i sierotipi: 38%	NA<br>NA
Canada (Quebec)[4] (2004)	2, 4, + 12 mesi	Tutti i sierotipi: 73%<br>Sierotipi del vaccino:<br>Serie infantile a due dosi: 99%<br>Schedula completa:100%	NA<br><br>92, 100%<br>82, 100%

[1]Bambini < 2 anni di età. Efficacia del vaccino calcolata da giugno 2008 (metodo Broome).
[2]Dati del 2005.
[3]Dati del 2004.
[4]Bambini < 5 anni di età. Da gennaio 2005 a dicembre 2007. Non è ancora disponibile l'efficacia totale per il programma di immunizzazione di routine 2+1

L'efficacia di Prevenar nella serie di immunizzazione 3+1 è stata anche osservata contro otite media acuta e polmonite a partire dalla sua introduzione nel programma di immunizzazione nazionale. In una valutazione retrospettiva di un database di un'importante assicurazione statunitense, risulta che, per i bambini di età inferiore ai 2 anni le visite per OMA sono state ridotte del 42,7% (95 % CI, 42,4-43,1 %) e le prescrizioni per OMA ridotte del 41,9%, rispetto al valore basale precedente all'introduzione di Prevenar (2004 vs 1997-99). In un'analisi simile, l'ospedalizzazione e le visite ambulatoriali per le polmoniti di qualunque origine furono ridotte del 52,4% e del 41,1%, rispettivamente. Per gli eventi specificatamente identificati come polmoniti pneumococciche, la riduzione osservata nell'ospedalizzazione e nella frequenza delle visite ambulatoriali furono del 57,6% e del 46,9% rispettivamente, nei bambini di età inferiore ai 2 anni, rispetto al valore basale precedente all'introduzione di Prevenar (2004 vs 1997-99). Mentre il rapporto diretto causa-effetto non può essere estrapolato dalle analisi osservazionali di questo tipo, questi risultati suggeriscono che Prevenar svolge un ruolo importante nel ridurre la patologia mucosale (OMA e polmonite) nella popolazione definita.

Ulteriori dati sulla immunogenicità di Prevenar 7valente: bambini con anemia a cellule falciformi

L'immunogenicità di Prevenar è stata analizzata con uno studio multicentrico in aperto effettuato su 49 neonati affetti da anemia a cellule falciformi. I bambini furono vaccinati con Prevenar (a partire dai due mesi di età, 3 dosi con un intervallo di un mese tra una dose e l'altra) e 46 di questi bambini furono vaccinati anche con un vaccino pneumococcico polisaccaridico 23valente all'età di 15-18 mesi. Dopo l'immunizzazione primaria, il 95,6% dei soggetti aveva livelli anticorpali di almeno 0,35 µg/ml per tutti sette sierotipi presenti in Prevenar. Dopo la vaccinazione polisaccaridica, un aumento significativo fu osservato nelle concentrazioni anticorpali contro i sette sierotipi, suggerendo che la memoria immunologica era ben radicata.

Proprietà farmacocinetiche

Per i vaccini non è richiesta la valutazione delle proprietà farmacocinetiche

Dati pre-clinici di sicurezza

Studi con una formulazione rappresentativa di Prevenar 13 non hanno rilevato alcun rischio particolare per la specie umana, secondo studi convenzionali di sicurezza farmacologica, tossicità per dosi ripetute, tossicità giovanile e tolleranza locale.

2. **OBIETTIVI E FINALITA' DELLO STUDIO**

Il progetto si propone di valutare, in modo prospettico, la risposta anticorpale a 2 dosi di PCV-13 e la prevalenza della colonizzazione nasofaringeo da *S. pneumoniae* in una popolazione HIV+ non ospedalizzata, correlando i dati anamnestici, clinici, sierologici e microbiologici.
La ricerca permetterà di valutare l'entità della colonizzazione da *S. pneumoniae* nei pazienti HIV positivi, la relativa percentuale di PNSSP, PRSP e/o multiresistenti, ed i sierotipi di pneumococco circolanti in questo tipo di popolazione, in relazione al titolo anticorpale al T0.
Con l'analisi dei dati clinici anamnestici ed i successivi follow up, sarà possibile distinguere i soggetti portatori a breve e a lungo termine, stabilirne i fattori di rischio e la frequenza di infezione.
Le analisi molecolari permetteranno di definire l'epidemiologia dell'acquisizione, valutando l'eventuale diffusione clonale di alcuni sierotipi di *S. pneumoniae* e la correlazione genetica fra isolati da portatore e da malattia.
Sarà valutato il profilo di sicurezza e l'efficacia in termini di risposta anticorpale (immunogenicità) della vaccinazione con vaccino coniugato 13-valente in due dosi a distanza di 8 settimane, in soggetti HIV positivi.
Il follow up microbiologico e sierologico, permetterà di valutare le dinamiche della colonizzazione nei soggetti sottoposti alla vaccinazione con due dosi di PCV13 analizzando in particolare se lo schema vaccinale proposto dal protocollo possa essere sicuro e se sia possibile, nei soggetti in studio:
- osservare una riduzione dello stato di portatore e/o dell'insorgenza di patologia invasiva da sierotipi vaccinali
- osservare colonizzazione e/o malattia dei sierotipi non vaccinali.

Sarà quindi possibile acquisire dati pilota rilevanti per successiva definizione di linee di condotta profilattiche e terapeutiche, per una corretta gestione clinica delle infezioni pneumococciche in soggetti HIV positivi.

The study outcomes are:
a) to determine the rate of nasal colonization by different pneumococcal serotypes in HIV-positive adults, in relation to baseline antibody titers at T0;
b) to define serological response after 2 doses of PCV13 vaccine (booster dose after 8 weeks) in HIV+ adults;
c) to evaluate the effect of 2 doses PCV13 vaccine in terms of nasal pneumococcal carriage and occurrence of invasive pneumococcal diseases;
d) to determine chemosusceptibility to different antibiotic and to evaluate the prevalence of multiresistent strains
e) to evaluate molecular epidemiology of pneumococcal isolates.

3. **DISEGNO SPERIMENTALE**

ARRUOLAMENTO: T0
Sarà richiesto consenso informato alla partecipazione allo studio e sarà somministrato questionario clinico-anamnestico.
Verranno quindi effettuati:
- tampone nasofaringeo per la ricerca di *S. pneumoniae*
- prelievo di campione sierico, crioconservazione a - 20°C per successive analisi sierologiche (dosaggio delle IgG verso i singoli antigeni polisaccaridici vaccinali ed eventuale valutazione della loro capacità opsonizzante).
- in assenza di controindicazioni, vaccinazione con PCV13, monitorizzando gli effetti avversi a breve (30 minuti), medio (<= 5 giorni) e lungo termine, con valutazione clinica nell'immediato post-vaccinale, intervista telefonica al 5° giorno post-vaccinale e raccordo clinico-anamnestico durante il successivo follow up (4, 8, 24 e 48 settimane); screening microbiologico, sierologico e clinico, secondo quanto previsto successivamente dal protocollo.

T4 (4 settimane):
- valutazione clinico-anamnestica
- tampone nasofaringeo per la ricerca di *S. pneumoniae*
- prelievo di campione sierico, crioconservazione a - 20°C per successive analisi sierologiche (dosaggio delle IgG verso i singoli antigeni polisaccaridici vaccinali ed eventuale valutazione della capacità opsonizzante)

T8 (8 settimane):
- valutazione clinico-anamnestica
- tampone nasofaringeo per la ricerca di *S. pneumoniae*
- prelievo di campione sierico, crioconservazione a - 20°C per successive analisi sierologiche (dosaggio delle IgG verso i singoli antigeni polisaccaridici vaccinali ed eventuale valutazione della capacità opsonizzante)
- somministrazione di booster con PCV-13, monitorizzando gli effetti avversi a breve (30 minuti), medio (<= 5 giorni) e lungo termine come precedentemente descritto per la prima

T24 (24 settimane) e T48 (48 settimane):
- valutazione clinico-anamnestica

- tampone nasofaringeo per la ricerca di *S. pneumoniae*
- prelievo di campione sierico, crioconservazione a - 20°C per successive analisi sierologiche (dosaggio delle IgG verso i singoli antigeni polisaccaridici vaccinali ed eventuale valutazione della capacità opsonizzante)

I tamponi saranno seminati su piastre di agar Columbia addizionato con il 5% di sangue di montone + colistina ed acido nalidixico, incubate a 37°C per 16-18 ore in atmosfera arricchita di CO2 al 5%.
Gli isolati saranno identificati e processati secondo metodiche classiche (caratteristiche morfologiche macro- e microscopiche, test di solubilità alla bile, sensibilità all'optochina ed agglutinazione al latice, con anticorpi diretti contro il polisaccaride capsulare), con successiva conservazione del ceppo a -80° C in brodo Wilkins-Chalgren , con il 20% di glicerolo, fino alle successive analisi.
Per evidenziare il possibile stato di portatore in contemporanea di due o più sierotipi, più colonie con morfologia suggestiva per *S. pneumoniae* saranno prelevate e sottoposte ad analisi.

Follow up clinico.
Un soggetto verrà considerato colonizzato da *S. pneumoniae* in caso di positività di almeno un tampone nasofaringeo. I pazienti che risulteranno colonizzati saranno seguiti per i 12 mesi successivi per rilevare l'insorgenza di eventuali infezioni invasive. Tutti i pazienti che svilupperanno un'infezione pneumococcica invasiva verranno seguiti fino alla risoluzione dell'episodio infettivo.

SECONDA FASE (studio sierologico, microbiologico ed analisi statistiche: 12 mesi)
Studio sierologico:
I campione sierici, raccolti e crioconservati a - 20°C, saranno analizzati presso l'Istituto di Malattie Infettive dell'UCSC del Policlinico Gemelli di Roma, secondo le indicazione dello WHO working group:
(http://www.who.int/biologicals/areas/vaccines/pneumo/Pneumo_final_23APRIL_2010.pdf).
Come cut-off di efficacia della risposta anticorpale si farà riferimento al valore clinicamente validato di 0.35 microgrammi/mL per ciascun antigene polisaccaridico vaccinale.

Analisi degli isolati da tampone nasofaringeo.

Tutti gli isolati di pneumococco ed il loro DNA estratto saranno raccolti presso la Clinica e Laboratorio di Malattie Infettive dell'Università di Siena per l'effettuazione delle ulteriori analisi microbiologiche:
-saggio di sensibilità in vitro dei ceppi crioconservati con metodica Kirby Bauer ed E-test per la determinazione delle CMI a penicillina, ceftriaxone
- saggio della sensibilità in vitro agli antibiotici di recente introduzione (quali quinopristina-dalfopristina, linezolid, tigeciclina)
- sierotipizzazione utilizzando antisieri dello Staten Serum Institute
- tipizzazione sierotipica tramite multiplex PCR, secondo le indicazioni riportate dal CDC (http://www.cdc.gov/ncidod/biotech/strep/pcr.htm)
- valutazione della clonalità tramite Multilocus Locus Sequenze Typing (MLST) amplificando e sequenziando sette geni "housekeeping" (*aroE*, *gdh*, *gki*, *recP*, *spi*, *xpt* e *ddl*), secondo protocolli già precedentemente descritti. Le sequenze ottenute saranno confrontate con quelle presenti nel database internazionale MLST (http://spneumoniae.mlst.net/), identificando i differenti sequence type (ST) ed effettuando inoltre una analisi filogenetica degli STs con identificazione dei complessi clonali (CCs) con l'ausilio del programma eBURST (http://eburst.mlst.net).
--analisi delle similitudini fenotipiche e valutazione della correlazione molecolare fra isolati da portatori e da successiva malattia invasiva
- selezione dei ceppi PNSSP di un campione significativo di isolati PSSP come comparazione ed esecuzione di:
-- amplificazione tramite PCR dei geni *pbp2b*, *pbp2x* (e *pbp1*a per i ceppi PRSP)
-- analisi dei polimorfismi di restrizione ("Restriction Fragment Lenght Polymorphism", RFLP) con l'ausilio del software" Diversity DatabaseTM, Bio-Rad, version
2.2.0
Isolati da infezione invasiva.
Gli isolati da malattia saranno analizzati dal punto di vista microbiologico secondo quanto precedentemente riportato per gli isolati da colonizzazione.

Analisi statistica.
I dati clinico-anamnestici raccolti all'arruolamento ed ai successivi follow up, saranno integrati con i risultati microbiologici e sierologici e verranno applicate le successive analisi statistiche per l'elaborazione delle correlazioni, elaborando i dati dei 50 pazienti del presente studio principale con i dati del sottostudio microbiologico e sierologico (Codice protocollo PNEUMO-HIV 2011).
Per la valutazione della risposta immunologica, saranno calcolate le medie geometriche dei titoli anticorpali (GMT) al basale e ai tempi 4,8,24 e 48 settimane; i GMT saranno comparati mediante il test t di Student per campioni appaiati.
Per l'analisi statistica dei risultati, le variabili quantitative saranno testate per la distribuzione e comparate con il test di Mann-Whitney o Kruskal-Wallis. Le differenze tra proporzioni saranno determinate utilizzando il test del chi-quadro

od il test di Fisher. I fattori associati a colonizzazione, infezione, antibioticoresistenza, risposta anticorpale efficiente (es. raggiungimento della soglia di IgG di >= 0.35 microgrammi/ml) saranno valutati mediante analisi di regressione logistica univariata e multivariata. L'analisi statistica sarà condotta utilizzando il programma SPSS ver. 17.0 (SPSS,Chicago, IL).

4.1 End-points dello studio

End-point primario: determinare la risposta anticorpale indotta da vaccinazione primaria con vaccino coniugato 13-valente e successiva dose booster ad 8 settimane in adulti HIV+;

Primary End-point: to define serological response after 2 doses of PCV13 vaccine (booster dose after 8 weeks) in HIV+ adults;

End-point secondario: Valutare l'entità della colonizzazione da differenti sierotipi di *S. pneumoniae* in soggetti HIV positivi, in relazione al livello anticorpale al T0; definire l'effetto delle due dosi di PCV13 sulla colonizzazione nasale e sull'insorgenza di infezioni pneumococciche invasive in adulti HIV+; valutare la chemiosensibilità degli isolati ai differenti antibiotici e stabilire la percentuale di isolati multiresistenti; valutare l'epidemiologia molecolare degli pneumococchi isolati.

4.2 Piano sperimentale

Procedura	Arruolamento T0	T4	T8	T24	T48
Consenso informato	X				
Questionario clinico-anamnestico	X	X	X	X	X
Valutazione clinica	X	X	X	X	X
Somministrazione vaccino	X		X		
Tampone nasofaringeo	X	X	X	X	X
Prelievo di campione sierico	X	X	X	X	X

5. POPOLAZIONE IN STUDIO

5.1 Dimensione del campione

Il campione di pazienti HIV positivi da arruolare nel presente studio principale (EudraCT number 2011-004518-40 Codice protocollo: PCV13-HIV2011) è previsto in numero di 50; dal sottostudio (codice protocollo PNEUMO-HIV2011) saranno arruolati i soggetti di controllo (50 pazienti HIV positivi e 100 pazienti HIV negativi).

I 50 soggetti HIV positivi, arruolati nello studio principale e mai vaccinati per pneumococco, saranno sottoposti a vaccinazione con PCV13 ed a follow up clinico, sierologico e microbiologico.

Un gruppo di 50 pazienti HIV positivi, in precedenza vaccinati con PPV23 da ≤ 5 anni, sarà sottoposto al medesimo follow up clinico, sierologico e microbiologico, senza interventi vaccinali e fungerà da gruppo di controllo.

In base ai dati di letteratura, si può stimare che - nei soggetti adulti HIV positivi - l'immunogenicità del PPV23 sia del 40% (Hung CC et al.2010); la risposta al vaccino coniugato eptavalente in bambini HIV positivi è viceversa riportata fra l'85 ed il 98% (Thanee C. 2011). La risposta attesa al vaccino coniugato 13-valente nella popolazione in studio è stimata pari al 70%.

Su tali basi, la numerosità del campione in studio e del gruppo di controllo (50+50) permette di ottenere una potenza statistica nel rilevare differenze pari all'81,5% con $p < 0{,}05$.

Un gruppo di soggetti HIV negativi di pari numerosità (100 pazienti) servirà come controllo al T0 per valutare la percentuale di colonizzazione nasofaringea in relazione ai livelli anticorpali in una popolazione non HIV.

I dati clinico-anamnestici raccolti all'arruolamento ed ai successivi follow up, saranno integrati con i risultati microbiologici e sierologici e verranno applicate le successive analisi statistiche per l'elaborazione delle correlazioni.

Per la valutazione della risposta immunologica, saranno calcolate le medie geometriche dei titoli anticorpali (GMT) al basale e ai tempi 4,8,24 e 48 settimane; i GMT saranno comparati mediante il test t di Student per campioni appaiati.

Per l'analisi statistica dei risultati, le variabili quantitative saranno testate per la distribuzione e comparate con il test di Mann-Whitney o Kruskal-Wallis. Le differenze tra proporzioni saranno determinate utilizzando il test del chi-quadro od il test di Fisher. I fattori associati a colonizzazione, infezione, antibioticoresistenza, risposta anticorpale efficiente (es. raggiungimento della soglia di IgG di >= 0.35 microgrammi/ml) saranno valutati mediante analisi di regressione logistica univariata e multivariata. L'analisi statistica sarà condotta utilizzando il programma

SPSS ver. 17.0 (SPSS,Chicago, IL).

5.2 Parametri di identificazione della popolazione in studio

Età > 18 anni
HIV sieropositività
CD4 ≥200 cell/µl in almeno due determinazioni consecutive precedenti al T0

5.3 Selezione dei pazienti

5.3.1 Criteri di inclusione

I Pazienti potranno essere inclusi nello studio solo se rispondono ai criteri di seguito riportati:

- età > 18 anni
- disponibilità dal parte del paziente o del tutore legale a fornire il proprio consenso libero ed informato
- accesso alle strutture in regime ambulatoriale o di Day Hospital
- CD4 ≥200 cell/µl in almeno due determinazioni consecutive precedenti al T0

Inclusion criteria:
- > 18 years old
- obtained informed consent
- outpatient
- CD4 ≥200 cells/µl in the last two evaluations before T0

5.3.2 Criteri di esclusione

I Pazienti non potranno essere inclusi nello studio se sarà presente anche solamente uno solo dei criteri di esclusione di seguito riportati:

- età >65 anni
- patologia infettiva acuta in atto
- antibiosi in atto o pregressa <= 7 giorni
- pregressa vaccinazione con PPV23 o con PCV7
- gravidanza
- terapia immunomodulante in atto
- Immunodepressione non HIV relata
-

Exclusion criteria:

- > 65 years old
- - presence of acute infectious disease
- - antibiotic therapy (ongoing or in the previous <= 7 days)
- - previous PPV23 or PCV7 vaccination
- Pregnancy
- Current immunomodulatory therapy
- Immunosuppression not HIV related

5.4 Criteri di conclusione del periodo di osservazione

Il termine dell'osservazione dei pazienti coinciderà con uno dei seguenti avvenimenti:

Termine del follow up = 48 settimane dopo l'arruolamento e/o 48 settimane dopo positività di tampone nasofaringeo per *S. pneumoniae*

6. TRATTAMENTO DEI SOGGETTI

6.1 Trattamento in studio

Schema di trattamento

50 soggetti HIV positivi saranno sottoposti al T0 a vaccinazione con PCV13, monitorizzando gli effetti avversi a breve (30 minuti), medio (<= 5 giorni) e lungo termine, con valutazione clinica nell'immediato post-vaccinale, intervista telefonica al 5° giorno post-vaccinale e raccordo clinico-anamnestico durante il successivo follow up (4, 8, 24 e 48 settimane).
Uno screening microbiologico (tampone nasofaringeo per colonizzazione da *S. pneumoniae*), sierologico (prelievo ematico per studio anticorpale) e clinico (anamnesi ed esame obiettivo) sarà effettuato al T0 e a 4, 8, 24 e 48 settimane.
Ad 8 settimane verrà somministrata una dose booster di PCV-13, monitorizzando gli effetti avversi a breve (30 minuti), medio (<= 5 giorni) e lungo termine come precedentemente descritto per la prima dose.

Acquisizione del farmaco:
Il vaccino verrà acquistato con i fondi ad hoc del progetto (per maggiori informazioni vedi sezione finanziamento del progetto).

6.1.1 Dosaggio, posologia e via di somministrazione

Due dosi, ciascuna da 0,5 ml, con un intervallo di 2 mesi, per iniezione intramuscolare nel muscolo deltoide del braccio.

6.1.2 Speciali avvertenze e precauzioni d'uso

Prevenar 13 non può essere somministrato per via intravascolare.
Come per tutti i vaccini iniettabili, devono essere sempre prontamente disponibili un appropriato trattamento ed una supervisione medica, nel caso si verificasse un raro evento anafilattico conseguente alla somministrazione del vaccino.
Prevenar 13 proteggerà solo dai sierotipi di *Streptococcus pneumoniae* inclusi nel vaccino, e non proteggerà da altri micro-organismi che causano patologia invasiva, polmonite o otite media.
Come con ogni vaccino, Prevenar 13 non protegge tutti gli individui che ricevono il vaccino contro le malattie causate da pneumococco.

Negli studi clinici, Prevenar 13 ha elicitato una risposta immunitaria verso tutti i tredici sierotipi inclusi nel vaccino. Tuttavia, la risposta immunitaria verso il sierotipo 3, registrata dopo la dose di richiamo non è aumentata al di sopra dei livelli raggiunti dopo la serie di vaccinazione infantile. La rilevanza clinica di questa osservazione, riguardo l'induzione di una memoria immunitaria al sierotipo 3, è sconosciuta.
Le proporzioni degli anticorpi funzionali (titolo OPA $\geq$ 1:8) che proteggono nei confronti dei sierotipi 1, 3 e 5 sono state alte. Comunque i titoli medi geometrici OPA erano più bassi di quelli raggiunti nei confronti di ciascuno degli altri sierotipi aggiuntivi rimanenti; la rilevanza clinica di ciò ai fini dell'efficacia protettiva non è nota.
La vaccinazione in gruppi ad elevato rischio deve essere valutata su base individuale. Dati specifici non sono ancora disponibili per Prevenar 13.
L'intervallo tra il vaccino pneumococcico 13valente coniugato (Prevenar 13) ed il vaccino pneumococcico polisaccaridico 23valente non deve essere inferiore alle 8 settimane.
Per i sierotipi del vaccino, la protezione contro l'otite media è prevista più bassa rispetto alla protezione contro la patologia invasiva. Poiché l'otite media è causata da molti organismi diversi dai sierotipi pneumococcici presenti nel vaccino, si prevede una protezione bassa contro tutte le otiti medie.

6.1.3 Confezionamento ed etichettatura del farmaco

Siringa preriempita, con o senza ago, confezione da 1 o 10:

- Confezione multipla che comprende 5 confezioni ognuna delle quali contiene 10 siringhe preriempite monodose (0,5 ml) con ago a parte
- Confezione multipla che comprende 5 confezioni ognuna delle quali contiene 10 siringhe preriempite monodose (0,5 ml) senza ago.

6.1.4 Drug Accountability

Lo Sperimentatore sarà responsabile della ricezione, conservazione ed utilizzazione del farmaco sperimentale; dovrà mantenere un adeguato inventario dei farmaci in deposito e somministrati ai singoli pazienti.

Conservazione dei materiali

La fornitura di farmaco sperimentale sarà inviata alla Farmacia del Centro Sperimentale e dovrà essere conservata presso la Farmacia del Centro o presso il Dipartimento/Istituto sede della sperimentazione, in luogo chiuso, ad accesso limitato al solo personale coinvolto, a temperatura non superiore a 25 °C e mantenuti nelle apposite scatole fino a loro utilizzo per proteggerli dall'esposizione alla luce.

Distribuzione del farmaco ai pazienti

Il farmaco sperimentale consegnato dovrà essere utilizzato solo ed esclusivamente per la sperimentazione in oggetto, secondo le modalità indicate nel protocollo.
Lo Sperimentatore ha l'obbligo di tenere la contabilità di tutto il farmaco somministrato ai pazienti per tutta la durata dello studio, utilizzando la modulistica predisposta.

Restituzione dei materiali
Alla conclusione dello studio, sarà effettuato un inventario finale del farmaco sperimentale, ed un apposito modulo sarà completato e firmato dallo Sperimentatore. Nel caso in cui mancassero materiali, occorrerà dichiarare e spiegare la discrepanza.

6.2 Randomizzazione
Non applicabile

6.3 Terapie farmacologiche concomitanti
HAART
Altri farmaci legati a patologie di base, non correlabili con infezione da *S. pneumoniae*

6.4 Sospensione/Interruzione del trattamento
I Pazienti devono essere informati della possibilità di interrompere lo studio in qualsiasi momento lo ritengano opportuno. Ogni interruzione deve essere documentata in modo completo nella SRD dallo Sperimentatore.
Sarà cura dello Sperimentatore seguire i Pazienti, per un periodo di tempo appropriato, in relazione all'evento che ha determinato il loro ritiro dallo studio, in maniera da verificare le condizioni cliniche, eventuali controlli di laboratorio, e/o la comparsa di eventi avversi anche a distanza di tempo dalla cessazione della terapia in studio.

7. VALUTAZIONE DELL'EFFICACIA

Nell'arco di un follow up di 12 mesi successivi all'arruolamento, i pazienti saranno seguiti con la tempistica prevista dal protocollo con particolare riferimento a:
- Valutazione della risposta anticorpale
- Valutazione dell'eventuale effetto sulla colonizzazione nasofaringea
- Valutazione dell'eventuale sviluppo di patologia pneumococcica invasiva e non invasiva

8. VALUTAZIONE DELLA SICUREZZA E TOLLERABILITA'

Dopo la somministrazione di ciascuna dose saranno monitorati gli effetti avversi a breve (30 minuti), medio (<= 5 giorni) e lungo termine, con valutazione clinica nell'immediato post-vaccinale, intervista telefonica al 5° giorno post-vaccinale e raccordo clinico-anamnestico durante il successivo follow up (4, 8, 24 e 48 settimane).

8.1 Eventi Avversi

Verrà valutata la comparsa di eventuali eventi e reazioni avverse (che siano o meno già riportate in scheda tecnica) nei tempi previsti dal protocollo ed autonomamente riferiti dal paziente indipendentemente dalla tempistica dello studio.
Il Paziente riceverà, durante e dopo lo studio, una adeguata assistenza medica per eventuali AE. Tutti gli AE saranno seguiti per determinarne l'esito.

8.1.1 Definizioni

<u>Evento avverso (Adverse Event)</u>

Per Evento Avverso viene *definito qualsiasi episodio sfavorevole di natura medica che si verifichi in un Paziente o in un soggetto partecipante a una sperimentazione clinica al quale sia stato somministrato un prodotto farmaceutico e che non deve necessariamente avere una relazione causale con questo trattamento*.
Un Evento Avverso può quindi essere qualsiasi segno (compreso un risultato anomalo di un esame ematochimico di laboratorio) o sintomo sfavorevole e non voluto, oppure una malattia associata all'impiego del prodotto medicinale in sperimentazione, per coincidenza temporale, sia essa correlata o meno al prodotto stesso.
I segni, sintomi e/o anormalità clinicamente significative negli esami di laboratorio già presenti prima dell'ingresso nello studio non sono considerati EA, a meno che non ricompaiano dopo la risoluzione o non presentino un peggioramento, per intensità o frequenza, rispetto alla condizione pre-esistente.
Qualsiasi AE dovrà essere riportato, insieme alla data, all'ora di insorgenza e di scomparsa ed alla durata (se inferiore alle 24 ore), nella Scheda Raccolta dati; dovranno altresì essere riportati intensità, relazione con il farmaco, azioni intraprese e trattamenti somministrati.
Per la valutazione dell'intensità, lo Sperimentatore utilizzerà le seguenti categorie: lieve, moderato, grave. Si tratta di una valutazione soggettiva e lo Sperimentatore si baserà sul proprio giudizio per mettere a confronto l'evento avverso insorto con eventi simili che si verificano nella pratica clinica.

Le linee guida per la valutazione dell'intensità sono le seguenti:

Grado lieve: segni o sintomi poco evidenti per il Paziente o che non costituiscono per lui un disagio. Gli eventi avversi di questo grado non influenzano alcuna attività o funzionalità. La prescrizione di farmaci non è di solito necessaria per alleviarli

Grado moderato: segni o sintomi di gravità sufficiente a causare fastidioso disagio. Le attività quotidiane risultano influenzate da eventi di questo grado. Possono essere necessari trattamenti.

Grado grave: segni o sintomi di gravità sufficiente a causare grave, intollerabile disagio o dolore. La severità può determinare la sospensione del trattamento sperimentale. Possono essere somministrati trattamenti.

Va comunque precisato che la gravità di un evento non è sinonimo di serietà.

E' responsabilità dello Sperimentatore assicurare che il Paziente riceva, durante e dopo lo studio, una adeguata assistenza medica per eventuali AE. Tutti gli AE dovranno essere seguiti per determinarne l'esito.

<u>Evento Avverso Serio (Serious Adverse Event, SAE)</u>

Un Evento Avverso Serio è qualsiasi evento medico sfavorevole che:
- *Determini il decesso del Paziente;*
- *Metta il Paziente in pericolo di vita;*
- *Ne richieda l'ospedalizzazione o determini il prolungamento di una ospedalizzazione preesistente;*
- *Provochi disabilità o incapacità persistente o significativa;*
- *Comporti una anomalia congenita o difetti alla nascita.*

Ciascun SAE deve essere comunicato agli organi competenti nei tempi e nei modi previsti dall'art. 17 del DECRETO LEGISLATIVO 24 giugno 2003, n.211 che viene di seguito riportato:
Art. 17
Notifica delle reazioni avverse serie
1. Il promotore della sperimentazione garantisce che tutte le informazioni pertinenti relative a sospette reazioni avverse serie inattese, che abbiano avuto esito letale per il soggetto della sperimentazione o mettano in pericolo di vita, vengano registrate e notificate al piu' presto al Ministero della salute, nonche' al/i Comitato/i etico/i interessato/i, e comunque entro sette giorni di calendario da quando il promotore della sperimentazione e' venuto a conoscenza del caso, e che successive informazioni pertinenti siano comunicate entro otto giorni dalla prima segnalazione.
2. Tutte le altre sospette reazioni avverse serie inattese sono notificate al Ministero della salute e al/i Comitato/i etico/i interessato/i, al piu' presto e comunque entro quindici giorni dal giorno in cui il promotore della sperimentazione ne e' venuto a conoscenza per la prima volta.
3. Lo sperimentatore comunica immediatamente al promotore della sperimentazione le reazioni di cui al presente articolo.
4. Il promotore della sperimentazione registra tutte le sospette reazioni avverse serie inattese di un medicinale in fase di sperimentazione portate a sua conoscenza.
5. Il promotore della sperimentazione informa anche gli altri sperimentatori.
6. Una volta all'anno per tutta la durata della sperimentazione clinica, come indicato dal decreto di cui all'articolo 18, il promotore della sperimentazione fornisce al Ministero della salute e ai Comitati etici coinvolti un elenco di tutti i sospetti di reazioni avverse serie osservati nel corso dell'intero periodo ed una relazione sulla sicurezza delle persone sottoposte alla sperimentazione clinica.
7. Il Ministero della salute provvede a che tutte le sospette reazioni avverse serie inattese di cui e' venuto a conoscenza vengano immediatamente inserite in una banca dati europea alla quale, a norma dell'articolo 11, comma 1, hanno accesso esclusivamente le Autorita' competenti degli Stati membri, l'Agenzia europea per la valutazione dei medicinali (EMEA) e la Commissione.
Art. 18.
Indicazioni relative alle relazioni
1. Con decreto del Ministro della salute, tenuto conto delle indicazioni dettagliate pubblicate dalla Commissione europea, sono stabilite le modalita' di raccolta, verifica e presentazione delle segnalazioni con dettagliate relazioni sugli eventi avversi o reazioni avverse, nonche' sulle modalita' di decodificazione riguardo alle reazioni avverse serie inattese.

Apposite Schede di segnalazione (SAE REPORT FORM) per la comunicazione del SAE saranno disponibili per lo Sperimentatore all'inizio della ricerca.

Relazioni di follow-up relative al Paziente che ha presentato un SAE dovranno successivamente essere inoltrate dallo Sperimentatore agli organi competenti fino alla risoluzione o stabilizzazione dell'evento. Se necessarie potranno essere richieste delle informazioni aggiuntive.

Reazione Avversa da Farmaco (ADR)

Si definisce Reazione Avversa *una risposta ad un prodotto medicinale che è nociva e non voluta, e che si manifesta al dosaggio normalmente utilizzato nell'uomo per la profilassi, diagnosi o terapia di una patologia o per modificarne funzioni fisiologiche.*
La dizione "risposta ad un prodotto medicinale" vuol significare che la correlazione causale fra il prodotto medicinale e l'Evento Avverso, può essere ragionevolmente possibile, **non può cioè essere esclusa**.
Pertanto una ADR è caratterizzata dal fatto che la correlazione causale tra l'AE ed il prodotto medicinale è giudicata almeno "possibile".
Nell'esprimere il giudizio di correlazione o meno con il farmaco, lo Sperimentatore si baserà sulle informazioni contenute nell'Investigator's Brochure, sulle condizioni cliniche e le terapie concomitanti, e sulla relazione temporale tra l'insorgenza dell'evento e la somministrazione del trattamento sperimentale.

La correlazione tra evento avverso e farmaco sarà espressa in accordo con le seguenti definizioni:

non correlato:	ogni reazione che non si manifesta entro un tempo ragionevole dalla somministrazione del farmaco in studio e che probabilmente è stata prodotta dallo stato clinico del Paziente o da altre terapie somministrate al Paziente;
correlazione improbabile:	ogni reazione che non si manifesta entro un tempo ragionevole dalla somministrazione del farmaco in studio o che probabilmente è stata prodotta dallo stato clinico del Paziente o da altre terapie somministrate al Paziente;
correlazione probabile:	ogni reazione che si manifesta entro un tempo ragionevole dalla somministrazione del farmaco in studio o che segue uno schema di reazioni note verso il farmaco in studio e che potrebbe non essere ragionevolmente spiegata dallo stato clinico del Paziente o da altre terapie somministrate al Paziente;
correlazione certa:	ogni reazione che si manifesta entro un tempo ragionevole dalla somministrazione del farmaco in studio e che segue uno schema di reazioni note verso il farmaco in studio e che

ricompaia alla reintroduzione del farmaco e/o che migliora con la sospensione del farmaco o con la riduzione delle dosi.

Reazione Avverse da Farmaco Inattese

Si definisce Reazione Avversa da Farmaco inattesa *una Reazione Avversa la cui natura o intensità non è contemplata nel materiale informativo sul prodotto (ad esempio: Investigator's Brochure per un prodotto in sviluppo; Riassunto delle Caratteristiche del Prodotto per farmaci in commercio).*

Lo Sperimentatore dovrà immediatamente riportare alla Direzione Sanitaria ed al Comitato Etico Indipendente di riferimento ogni Reazione Avversa da Farmaco che sia contemporaneamente seria ed inattesa.

9. ACCESSO DIRETTO AI DOCUMENTI ORIGINALI

Lo Sperimentatore/Istituzione deve permettere alle Autorità Regolatorie, nazionali, ed al personale designato dal Comitato Etico Indipendente o dallo Sperimentatore/Proponente l'accesso diretto, e relativa verifica, a tutta la documentazione originale dello studio, inclusi i moduli di Consenso Informato firmati dai soggetti inseriti nello studio e le cartelle ospedaliere e/o registri ambulatoriali. Coloro che hanno accesso diretto a tale documentazione devono prendere ogni ragionevole precauzione per mantenere riservata l'identità dei soggetti e le informazioni confidenziali di proprietà dello Sperimentatore/Proponente, nel rispetto delle disposizioni normative applicabili.

10. PROCEDURE DI CONTROLLO E DI ASSICURAZIONE DELLA QUALITA'

10.1 Scheda Raccolta Dati

La Scheda Raccolta Dati (SRD) è il documento cartaceo appositamente predisposto per lo studio che viene fornito allo Sperimentatore per la registrazione dei dati sperimentali richiesti dal protocollo di studio per ciascun soggetto partecipante alla sperimentazione.

Nella SRD dovranno essere raccolti i dati sperimentali relativi a:

- Dati anagrafici del paziente (sesso, età)
- Anamnesi Fisiologica e Patologica
- Fattori di rischio per infezioni pneumocociche ed immunodepressione non HIV-relata
- Valori ematochimici e funzionali
- Terapia farmacologica concomitante
- Pregresse patologie pneumococciche
-

Al termine dell'osservazione del paziente così come definita nel § 5.4, lo Sperimentatore dovrà compilare l'apposita scheda di "Fine studio".

10.2 Audits

Lo Sperimentatore/Istituzione deve permettere alle Istituzioni competenti di effettuare gli audit, come parte integrante del sistema di assicurazione della qualità. L'Audit è un controllo, indipendente e separato dal monitoraggio, delle attività e dei documenti dello studio per verificare se siano state condotte le attività pertinenti allo studio e se i dati siano stati registrati, analizzati e trasmessi in conformità al protocollo, alla GCP, alle SOPs e alle disposizioni normative applicabili.

10.3 Ispezioni

Lo Sperimentatore/Istituzione deve permettere alle Autorità Regolatorie nazionali, di effettuare Ispezioni.
L'Ispezione, da parte di una o più Autorità Regolatorie, consiste nella revisione ufficiale di documenti, strutture, registrazioni e ogni altra risorsa considerata dalle autorità stesse collegata allo studio clinico.

11. GESTIONE DEI DATI CLINICI

La gestione dei dati sperimentali e la loro analisi sarà affidata agli Sperimentatori (Principal Investigators ed ai Co-Sperimentatori e).
Gli eventuali collaboratori per l'analisi e la gestione dei dati, quali persone qualificate designate dagi Sperimentori, riceveranno i dati in forma anonima, per cui non sarà possibile risalire all'identità dei pazienti nella fase di analisi dei dati e nelle successive fasi di elaborazione e pubblicazione degli stessi.

12. ANALISI STATISTICA

L'analisi statistica verrà effettuata integrando i dati del presente studio (Codice protocollo PCV13-HIV2011) con i dati ricavati dal sottostudio microbiologico e sierologico (codice protocollo PNEUMO-HIV 2011).
I dati clinico-anamnestici raccolti all'arruolamento ed ai successivi follow up, saranno integrati con i risultati microbiologici e sierologici e verranno applicate le successive analisi statistiche per l'elaborazione delle correlazioni.
Per la valutazione della risposta immunologica, saranno calcolate le medie geometriche dei titoli anticorpali (GMT) al basale e ai tempi 4,8,24 e 48 settimane; i GMT saranno comparati mediante il test t di Student per campioni appaiati.
Per l'analisi statistica dei risultati, le variabili quantitative saranno testate per la distribuzione e comparate con il test di Mann-Whitney o Kruskal-Wallis. Le differenze tra proporzioni saranno determinate utilizzando il test del chi-quadro od il test di Fisher. I fattori associati a colonizzazione, infezione, antibioticoresistenza, risposta anticorpale efficiente (es. raggiungimento della soglia di IgG di >= 0.35 microgrammi/ml) saranno valutati mediante analisi di regressione logistica univariata e multivariata. L'analisi statistica sarà condotta utilizzando il programma SPSS ver. 17.0 (SPSS,Chicago, IL).

13. ASPETTI ETICI

Tutte le parti coinvolte nello studio concordano e verificheranno che questa sperimentazione sia condotta in conformità ai principi etici, che traggono la loro origine dalla Dichiarazione di Helsinki (Appendice I), alle linee guida della Buona Pratica Clinica (GCP-ICH DM n. 162 del 15/07/97) e alle disposizioni normative applicabili.

13.1 Autorizzazioni Etiche

Lo Sperimentatore/Istituzione ha la responsabilità di sottoporre questo protocollo clinico al Comitato Etico Indipendente (CEI) locale per l'approvazione, prima di arruolare i soggetti nello studio. Lo Sperimentatore dovrà fornire al CEI tutti i documenti necessari per la richiesta dell'approvazione.
Copia dell'approvazione del CEI deve essere trasmessa allo Sperimentatore/Proponente prima dell'inizio dello studio.
Questo studio verrà sottoposto per l'autorizzazione alle Autorità Sanitarie in accordo con le norme e le leggi attualmente in vigore.

13.2 Consenso Informato

Prima dell'inizio dello studio, le "Informazioni scritte per il Paziente" da fornire ai soggetti ed il modulo/i di Consenso Informato devono essere sottoposti all'esame e approvazione da parte del Comitato Etico Indipendente locale, contestualmente al protocollo.
Il Consenso Informato deve essere richiesto, ottenuto e documentato dallo Sperimentatore in ottemperanza alle disposizioni normative applicabili, alla GCP ed ai principi etici che si originano dalla Dichiarazione di Helsinki.
Nelle "Informazioni scritte per il Paziente" saranno fornite le garanzie relative alla protezione e la tutela dei dati personali ai sensi del Decreto legislativo 30.6.03 n. 196 codice privacy.

14. PROCEDURE AMMINISTRATIVE

14.1 Cambiamenti nella condotta dello studio o analisi pianificate

Ogni cambiamento nella condotta dello studio viene definito "Emendamento al Protocollo Clinico": con tale termine si intende qualunque modifica che viene apportata al protocollo sperimentale dopo l'approvazione finale dello Sperimentatore/Proponente e degli altri Sperimentatori.
Gli emendamenti al protocollo sono modifiche di un documento che ha valore legale per cui come tali devono essere approvati e firmati in duplice copia in originale dagli stessi firmatari del protocollo.
Tutti gli emendamenti devono essere sottoposti al giudizio del Comitato Etico che ha approvato il protocollo in studio, prima di poter essere applicati.
E' responsabilità dello Sperimentatore sottoporre l'emendamento al Comitato Etico Indipendente di riferimento ed ottenerne un documento di approvazione.
L'approvazione scritta avrà le stesse modalità di distribuzione e di conservazione di quelle previste per il protocollo in studio.
Nel caso in cui il cambiamento riguardi unicamente modifiche degli aspetti amministrativi o logistici della sperimentazione stessa, l'emendamento che ne consegue dovrà essere semplicemente notificato al Comitato Etico.
Si deve infine tenere presente che ove l'emendamento alteri sostanzialmente il disegno dello studio o i potenziali rischi a cui il paziente è esposto, ciascun paziente dovrà essere informato e dovrà confermare per iscritto la sua volontà di continuare lo studio.
Un apposito modulo di informazione e di consenso sarà preparato dallo Sperimentatore/Proponente ed approvato dal Comitato Etico Locale.

14.2 Sospensione / Interruzione dello studio

Lo studio nella sua gestione globale potrà essere interrotto prematuramente, su richiesta dello Sperimentatore/Proponente o di altri Sperimentatori o da parte del Comitato Etico, qualora:

- nuove informazioni di carattere tossicologico, farmacologico o clinico renderanno inaccettabile il razionale ed il disegno sperimentale dello studio
- il rate di reclutamento dei pazienti si dimostrerà essere inefficace
- il Centro non rispetti le specifiche richieste del protocollo sperimentale, soprattutto in merito alla valutazione dei criteri di inclusione/esclusione dei paziente
- il Centro non è in grado di soddisfare le richieste delle le linee guida di Buona Pratica Clinica (GCP-ICH DM n. 162 del 15/07/97)
- il Centro non è in grado di applicare le disposizioni normative vigenti

Lo Sperimentatore dovrà provvedere ad informare il Comitato Etico di riferimento dell'Istituzione di appartenenza del Centro Sperimentale, giustificando la prematura interruzione dello studio.

14.3 Archiviazione

Lo Sperimentatore/Istituzione deve provvedere alla conservazione dei documenti essenziali dello studio come specificato dalla GCP e in accordo alle disposizioni normative applicabili. Lo Sperimentatore/Istituzione deve adottare le misure necessarie per impedire la distruzione accidentale o prematura.
Lo Sperimentatore/Istituzione deve conservare i documenti essenziali per almeno 7 anni dal termine dello studio in accordo a quanto previsto dalle GCP/ICH ed in conformita' al D.Lvo 200/2007, art. 18, comma 1.
Tuttavia, questi documenti devono essere conservati per periodi più lunghi se richiesto dalle disposizioni normative applicabili.

Confidenzialità e pubblicazione delle informazioni

Ai fini della partecipazione al presente studio, è necessario raccogliere i dati personali dei pazienti, ovvero il sesso, la data di nascita, i dati clinici ed eventuali altri dati in quanto funzionali alla corretta esecuzione dello studio. I dati in oggetto saranno trattati nel pieno rispetto della legge relativa alla Tutela dei dati personali e successive modifiche ed integrazioni. In ogni caso si tratterà sempre di dati da Lei comunicati e non di dati pervenuti da altre fonti.
I dati indicati saranno raccolti dal medico dello studio, in forma anonima, esclusivamente in funzione della realizzazione dello studio e della partecipazione ad esso.
Il medico dello studio identificherà' i pazienti con un codice identificativo alfanumerico: i dati non saranno diffusi - cioè resi pubblici, ad esempio attraverso pubblicazioni scientifiche, statistiche, convegni scientifici - se non in forma rigorosamente anonima. L'accesso diretto alla documentazione clinica originale può` essere richiesto dallo sperimentatore

e dai suoi collaboratori coinvolti nello studio, dal Comitato Etico o dalle Autorità regolatorie di governo, quali ad esempio personale del Ministero della Sanità' italiano, della Food and Drug Administration (Stati Uniti) o della Unione Europea, per verificare che le informazioni riportate sui documenti dello studio siano corrette.
Detta attività di verifica verra` comunque sempre svolta sotto la supervisione del medico dello studio ed eseguita in modo del tutto professionale.

Copertura assicurativa di responsabilità civile
STUDIO principale PCV13-HIV2011: quotazione Assiteca 08/09/2011 per il centro coordinatore (AOUS) e per il centro satellite (USCS).
Durata della copertura: 12 mesi con data di effetto da comunicarsi.
Massimali: Per paziente: Euro 1.000.000 - Per protocollo: Euro 5.000.000
Franchigia per sinistro (non opponibile a terzi): Nessuna
Azioni legali presso tribunali italiani
Garanzia Postuma: 3 anni
Premio lordo: Euro 5.379,00
Tasso di regolazione per eventuali pazienti in eccesso a n° 50
Euro 37 lordo a paziente
Sottostudio microbiologico e sierologico PNEUMO-HIV 2011 per il centro coordinatore (AOUS), lo sperimentatore proponente, responsabile per il centro di Siena, ha richiesto la possibilità di avvalersi del meccanismo della responsabilità diretta in vigore all'interno della struttura di appartenenza.

Finanziamento della sperimentazione
La sperimentazione sarà interamente finanziata da fondo ministeriale *ad hoc* (PRIN Programmi di Ricerca Scientifica di Rilevante Interesse Nazionale, Cofinanziamento D.M. 19 marzo 2010 n°51)

RESPONSABILITA' DELLO SPERIMENTATORE
Lo Sperimentatore è consapevole di essere responsabile di tutte le azioni da lui delegate agli altri membri del Suo staff designati alla conduzione dello studio. Eccetto dove specificatamente richiesto, il termine "Sperimentatore" utilizzato in questo protocollo e sulle Schede Raccolta Dati si riferisce allo Sperimentatore o a persona qualificata da lui designata che quindi potrà eseguire attività relative al trial clinico e firmare in Sua vece i documenti dello studio.
Lo Sperimentatore è tenuto a condurre lo studio in conformità al protocollo di studio e in accordo con le Norme della Buona Pratica Clinica (GCP-ICH-DM n. 162 del 15/07/97) e con i principi della Dichiarazione di Helsinki (1964) e successive revisioni (Appendice I)

RAPPORTO FINALE DELLO STUDIO

Al termine della ricerca, entro 6 mesi dalla fine dello studio, sarà redatto dallo proponente e/o da persona delegata un Rapporto Clinico Finale dello studio contenente i commenti clinici basati sui dati generati dall'elaborazione statistica.

17. FASI E TEMPISTICA

Vengono di seguito riportate le fasi della sperimentazione ed una previsione della relativa tempistica di attuazione:

- Incontro preliminare con i centri arruolati<br><br>- Richiesta di approvazione Comitati Etici	Settembre 2011<br><br>Ottobre 2011
- Arruolamento dei pazienti<br><br>- Primi risultati (descrizione del campione)	Novembre 2011 – Ottobre 2012<br><br>Novembre 2012
- Analisi dei dati	Novembre 2012- ottobre 2013
- Stesura del report finale di ricerca	Ottobre 2013

18. DISCUSSIONE ETICA

Lo studio sarà condotto in accordo con i principi etici sanciti dalla Dichiarazione di Helsinki nella sua ultima revisione. La partecipazione allo studio sarà subordinata all'ottenimento del consenso libero e informato (allegato) e saranno salvaguardati i diritti sanciti dalla legge in materia di protezione dei dati personali (Decreto Legislativo 30/6/2003 n. 196 e

successive modificazioni) delle persone sottoposte a valutazione. Ciascun gruppo collaborante dovrà sottoporre lo studio al parere del proprio Comitato Etico Locale.

18. BIBLIOGRAFIA

1. Musher D. M. 2005. Streptococcus pneumoniae. P. 2342-2411. In Mandell G.L., Bennet J.E., Dolin R. Principles and practice of infectious diseases (6th ed.). Churchill Livingston. N.Y

2. Cartwight K. 2002. Pneumococcal disease in western Europe: burden of disease, antibiotic resistance and management. Europ. J. Pediatr. 161:188-195.

3. Redd SC et al. 1990. The role of human immunodeficiency virus infection in pneumococcal bacteremia in San Francisco residents. J Infect Dis 162: 1012-1017.

4. Klugman KP et al. 2007. HIV and pneumococcal disease. Curr Opin Infect Dis 20:11-15.

5. Frankel et al. 1996. Invasive pneumococcal disease: clinical features, serotypes and antimicrobial resistance patterns in cases involving patient with and without human immunodeficiency virus infection. Cin Infect Dis 23:577-584

6. Rodriguez-Barradas et al. 1997. Colonization by Streptococcus pneumoniae among human immunodeficiency virus-infected adults: prevalence of antibiotic resistance, impact of immunization, and characterization by polymerase chain reaction with BOX primers of isolates from persistent S. pneumoniae carriers. J Infect Dis 175:590-597.

7. Bogaert D et al. 2004. Streptococcus pneumoniae colonisation: the key to pneumococcal disease. Lancet Infect Dis 4:144-154.

8. Penaranda M et al. 2007. Effectiveness of polysaccharide pneumococcal vaccine in HIV-infected patients: a case control study. Clin Infect Dis 45: 82-87.

9. Teshale EH, et al. Effectiveness of 23-valent polysaccharide pneumococcal vaccine on pneumonia in HIV-infected adults in the United States, 1998--2003. Vaccine. 2008;26(46):5830-5834.

10. Rodriguez-Barradas MC, et al. Antibody to capsular polysaccharides of Streptococcus pneumoniae after vaccination of human immunodeficiency virus-infected subjects with 23-valent pneumococcal vaccine. J Infect Dis. 1992. 165:553-6.

11. Kroon FP, et al. Antibodies against pneumococcal polysaccharides after vaccination in HIV-infected individuals: 5-year follow-up of antibody concentrations. Vaccine. 1999;18:524-30.

12. Ahmed F, et al. Effect of human immunodeficiency virus type 1 infection on the antibody response to a glycoprotein conjugate pneumococcal vaccine: results from a randomized trial. J Infect Dis. 1996;173:83-90.

13. Nielsen H, et al.. Rapid loss of specific antibodies after pneumococcal vaccination in patients with human immunodeficiency virus-1 infection. Scand J Infect Dis. 1998;30:597-601.

14. Tasker SA, et al.. Reimmunization with 23-valent pneumococcal vaccine for patients infected with human immunodeficiency virus type 1: clinical, immunologic, and virologic responses. Clin Infect Dis. 2002;34:813-21.

15. Rodriguez-Barradas MC, et al. IgG antibody to pneumococcal capsular polysaccharide in human immunodeficiency virus-infected subjects: persistence of antibody in responders, revaccination in nonresponders, and relationship of immunoglobulin allotype to response. J Infect Dis. 1996;173:1347-53.

16. Mond JJ, et al.. T cell-independent antigens type 2. Annu Rev Immunol. 1995;13:655-92. Review.

17. O'Brien KL, et al. Combined schedules of pneumococcal conjugate and polysaccharide vaccines: is hyporesponsiveness an issue? Lancet Infect Dis. 2007;7:597-606.

18. Onwubiko C. et al 2008. Cross-sectional study of nasopharingeal carriage of Streptococcus pneumoniae in human immunodeficiency virus-infected adults in the conjugate vaccine era. J Clin Microbiol 46:3621-3625.

19. Munoz-Almagro C. et al. Emergence of invasive pneumococcal disease caused by nonvaccine serotypes in the era of 7-valent conjugate vaccine. Clin Infect Dis 2008. 46:174-182.

20. Huang SS, et al. Post-PCV7 changes in colonizing pneumococcal serotypes in 16 Massachusetts communities, 2001 and 2004. Pediatrics. 2005;116:e408-13. Erratum in: Pediatrics. 2006;117:593-4

21. CDC. Invasive pneumococcal disease in children 5 years after conjugate vaccine introduction--eight states, 1998-2005. MMWR Morb Mortal Wkly Rep. 2008;57:144-8.

22. CDC. Direct and indirect effects of routine vaccination of children with 7-valent pneumococcal conjugate vaccine on incidence of invasive pneumococcal disease--United States, 1998-2003. MMWR Morb Mortal Wkly Rep. 2005;54:893-7.
23. Whitney CG, et al. Active Bacterial Core Surveillance of the Emerging Infections Program Network. Decline in invasive pneumococcal disease after the introduction of protein-polysaccharide conjugate vaccine. N Engl J Med. 2003;348:1737-46.
24. Pilishvili T, et al. Active Bacterial Core Surveillance/Emerging Infections Program Network. Sustained reductions in invasive pneumococcal disease in the era of conjugate vaccine. J Infect Dis. 2010;201:32-41.
25. Millar EV, et al. Effect of community-wide conjugate pneumococcal vaccine use in infancy on nasopharyngeal carriage through 3 years of age: a cross-sectional study in a high-risk population. Clin Infect Dis. 2006;43:8-15.
26. O'Brien KL, et al. Effect of pneumococcal conjugate vaccine on nasopharyngeal colonization among immunized and unimmunized children in a community-randomized trial. J Infect Dis. 2007;196:1211-20.
27. CDC. Licensure of a 13-valent pneumococcal conjugate vaccine (PCV13) and recommendations for use among children - Advisory Committee on Immunization Practices (ACIP), 2010. MMWR Morb Mortal Wkly Rep. 2010;59:258-61.
28. Esposito S, et al. Safety and immunogenicity of 13-valent pneumococcal conjugate vaccine compared to 7-valent pneumococcal conjugate vaccine given as a 3-dose series with routine vaccines in healthy infants and toddlers. Clin Vaccine Immunol. 2010 Apr 28.
29. French N, et al. A trial of a 7-valent pneumococcal conjugate vaccine in HIV-infected adults. N Engl J Med. 2010;362:812-22.
30. Kroon FP, et al. Enhanced antibody response to pneumococcal polysaccharide vaccine after prior immunization with conjugate pneumococcal vaccine in HIV-infected adults. Vaccine. 2000;19:886-94.
31. Lesprit P, et al. ANRS 114-Pneumovac Study Group. Immunological efficacy of a prime-boost pneumococcal vaccination in HIV-infected adults. AIDS. 2007;21:2425-34.
32. Peñaranda M, et al. Majorcan Pneumococcal Study Group. Conjugate and polysaccharide pneumococcal vaccines do not improve initial response of the polysaccharide vaccine in HIV-infected adults. AIDS. 2010;24:1226-8.
33. Scott DA, et al. Phase 1 trial of a 13-valent pneumococcal conjugate vaccine in healthy adults. Vaccine. 2007;25:6164-6.
34. Hung CC et al. ,A 5-year longitudinal follow-up study of serological responses to 23-valent pneumococcal polysaccharide vaccination among patients with HIV infection who received highly active antiretroviral therapy. HIV Medicine 2010; 11: 54-63.
35. Thanee C. et al. The immunogenicity and safety of pneumococcal conjugate vaccine in human immunodeficiency virus-infected Thai children. Vaccine. 2011; 29:5886-91

Appendice 1

<u>WORLD MEDICAL ASSOCIATION</u>

<u>DECLARATION OF HELSINKI</u>

Ethical Principles for Medical Research Involving Human Subjects

Adopted by the 18th WMA General Assembly, Helsinki, Finland, June 1964
And amended by the
29th WMA General Assembly, Tokyo, Japan, October 1975
35th WMA General Assembly, Venice, Italy, October 1983
41st WMA General Assembly, Hong Kong, September 1989
48th WMA General Assembly, Somerset West, Republic of South Africa, October 1996
and the
52nd WMA General Assembly, Edinburgh, Scotland, October 2000

A INTRODUCTION

1. The World Medical Association has developed the Declaration of Helsinki as a statement of ethical principles to provide guidance to physicians and other participants in medical research involving human subject. Medical research involving human subject includes research on identifiable human material or identifiable data.
2. It is the duty of physician to promote and safeguard the health of the people. The physician's knowledge and conscience are dedicated to the fulfilment of this duty.
3. The Declaration of Geneva of the World Medical Association binds the physician with the words, "The health of my patient will be my first consideration," and the International Code of Medical Ethics declares that, "A physician shall act only in the patient's interest when providing medical care which might have the effect of weakening the physical and mental condition of the patient."
4. Medical progress is based on research which ultimately must rest in part on experimentation involving human subjects.
5. In medical research on human subjects, considerations related to the wee-being of the human subject should take precedence over the interests of science and society.
6. The primary purpose of medical research involving human subjects is to improve prophylactic, diagnostic and therapeutic procedures and the understanding of the aetiology and pathogenesis of disease. Even the best proven prophylactic, diagnostic, and therapeutic methods must continuously be challenged through research for their effectiveness, efficiency, accessibility and quality.
7. In current medical practice and in medical research, most prophylactic, diagnostic and therapeutic procedures involve risks and burdens.
8. Medical research is subject to ethical standards that promote respect for all human beings and protect their health and rights. Some research populations are vulnerable and need special protection. The particular needs of the economically and medically disadvantaged must be recognized. Special attention is also required for those who cannot dive or refuse consent for themselves, for those who may be subject to giving consent under duress, for those who will not benefit personally from the research and for those whom the research is combined with care.
9. Research Investigators should be aware of the ethical, legal and regulatory requirements for research on human subjects in their own countries as well as applicable international requirements. No national ethical, legal or regulatory requirement should allowed to reduce or eliminate any of the protections for human subjects set forth in this Declaration.

B BASIC PRINCIPLES FOR ALL MEDICAL RESEARCH

10. It is the duty of the physician in medical research to protect the life, health, privacy, and dignity of the human subject.
11. Medical research involving human subject must conform to generally accepted scientific principles, he based on a thorough knowledge of the scientific literature, other relevant sources of information, and on adequate laboratory and, where appropriate, animal experimentation.
12. Appropriate caution must be exercised in the conduct of research which may affect the environment, and the welfare of animals used for research must be respected.
13. The design and performance of each experimental procedure involving human subject should be clearly formulated in an experimental protocol. This protocol should be submitted for consideration, comment, guidance, and where appropriate, approval to a specially appointed ethical review committee, which must be independent of the Investigator, the sponsor of any other kind of undue influence. This independent committee has the right to monitor ongoing trials. The researcher has the obligation to provide monitoring information to the committee, especially any serious adverse events. The researcher should also submit to the committee, for review, information regarding funding, sponsors, institutional affiliations, other potential conflicts of interest and incentives for subjects.

14. The research protocol should always contain a statement of the ethical considerations involved and should indicate that there is compliance with the principles enunciated in this Declaration.
15. Medical research involving human subject should be conducted only by scientifically qualified persons and under the supervision of a clinically competent medical person. The responsibility for the human subject must always rest with a medically qualified person and never rest on the subject of the research, even though the subject has given consent.
16. Every medical research project involving human subjects should be preceded by careful assessment of predictable risks and burdens in comparison with foreseeable benefits to the subject or to others. This does not preclude the participation of healthy volunteers in medical research. The design of all studies should be publicly available.
17. Physicians should abstain form engaging in research projects involving human subjects unless they are confident that the risks involved have been adequately assessed and can be satisfactorily managed. Physicians should cease any investigation if the risks are found to outweigh the potential benefits or if there is conclusive proof of positive and beneficial results.
18. Medical research involving human subjects should only be conducted if the importance of the objective outweighs the inherent risk and burdens to the subject. This is especially important when the human subjects are healthy volunteers.
19. Medical research is only justified if there is a reasonable likelihood that the populations in which the research is carried out stand to benefit from the results of the research.
20. The subjects must be volunteers and informed participants in the research project.
21. The right of research subject to safeguard their integrity must always be respected. Every precaution should be taken to respect the privacy of the subject, the confidentiality of the patient's information and to minimize the impact of the study on the subject's physical and mental integrity and on the personality of the subject.
22. In any research on human beings, each potential subject must be adequately informed of the aims, methods, sources of funding, any possible conflicts of interest, institutional affiliations of the researcher, the anticipated benefits and potential risks of the study and the discomfort it may entail. The subject should be informed of the right to abstain from participation in the study or to withdraw consent to participate at any time without reprisal. After ensuring that the subject has understood the information, the physician should then obtain the subject's freely-given informed consent, preferably in writing. If the consent cannot be obtained in writing, the non-written consent must be formally documented and witnessed.
23. When obtaining informed consent for the research project the physicians should be particularly cautious if the subject is in a dependent relationship with the physician or may consent under duress. In that case the informed consent should be obtained by a well-informed physician who is not engaged in the investigation and who is completely independent of this relationship.
24. For a research subject who is legally incompetent, physically or mentally incapable of giving consent or is a legally incompetent minor, the Investigator must obtain informed consent from the legally authorized representative in accordance with applicable law. These groups should not be included in research unless the research is necessary to promote the health of the population represented and this research cannot instead be performed on legally competent persons.
25. When a subject deemed legally incompetent, such as a minor child, is able to give assent to decisions about participation in research, the Investigator must obtain the assent in addition to the consent of the legally authorized representative.
26. Research on individuals from whom it is not possible to obtain consent, including proxy or advance consent, should be done only if the physical/mental condition that prevents obtaining informed consent is a necessary characteristic of the research population. The specific reasons for involving research subjects with a condition that renders them unable to give informed consent should be stated in the experimental protocol for consideration and approval of the review committee. The protocol should state that consent to remain in the research should be obtained as soon as possible from the individual or a legally authorized surrogate.
27. Both authors and publishers have ethical obligations. In publication of the results of research, the investigators are obliged to preserve the accuracy of the results. Negative as well as positive results should be published or otherwise publicly available. Sources of funding, institutional affiliations and any possible conflicts of interest should be declared in the publication. Reports of experimentation not in accordance with the principles laid down in this Declaration should not be accepted for publication.

C ADDITIONAL PRINCIPLES FOR MEDICAL RESEARCH COMBINED WITH MEDICAL CARE

28. The physician may combine medical research with medical care, only to the extent that research is justified by its potential prophylactic, diagnostic or therapeutic value. When medical research is combined with medical care, additional standards apply to protect the patients who are research subjects.
29. The benefits, risks, burdens and effectiveness of a new method should be tested against those of the best current prophylactic, diagnostic or therapeutic value. When medical research is combined with medical care, additional standards apply to protect the patients who are research subjects.
30. At the conclusion of the study, every patient entered into the study should be assured of access to the best proven prophylactic, diagnostic or therapeutic method exists.

31. The physician should fully inform the patient which aspects of the care are related to the research. The refusal of a patient to participate in a study must never interfere with the patient-physician relationship.
32. In the treatment of a patient, where proven prophylactic, diagnostic and therapeutic methods do not exist or have been ineffective, the physician, with informed consent from the patient, must be free to use3 unproven or new prophylactic, diagnostic and therapeutic measures, if in the physician's judgement it offers hope of saving life, re-establishing health or alleviating suffering. Where possible, these measures should be made the object of research, designed to evaluate their safety and efficacy. In all cases, new information should be recorded and, where appropriate, published. The other relevant guidelines of this Declaration should be followed.

Note of clarification on par. 29 of the WMA Declaration of Helsinki

The WMA is concerned that paragraph 29 of the revised Declaration of Helsinki (October 2000) has led to diverse interpretations and possible confusion. It hereby reaffirms its position that extreme care must be taken in making use of a placebo-controlled trial and that in general this methodology should only be used in the absence of existing proven therapy.

However, a placebo-controlled trial may be ethically acceptable, even if proven therapy is available, under the following circumstances:

- Where for compelling and scientifically sound methodological reasons its use is necessary to determine the efficacy or safety of a prophylactic, diagnostic or therapeutic method; or
- Where a prophylactic, diagnostic or therapeutic method is being investigated for a minor condition and the patients who receive placebo will not be subject to any additional risk of serious or irreversible harm.

All other provisions of the Declaration of Helsinki must be adhered to, especially the need for appropriate ethical and scientific review.

SCHEDA CLINICA PROTOCOLLO **PRINCIPALE**

(EudraCT number 2011-004518-40 - Codice protocollo PCV13-HIV2011)

SCHEDA DI ARRUOLAMENTO

CENTRO SPERIMENTALE____

ID paziente____ Data di Nascita:____________________

RESIDENZA:____________________________________ Tel:______________________________

DATA di ARRUOLAMENTO __________________________

Sesso: □ M □ F Etnia: ____

CRITERI DI INCLUSIONE

- Età > 18 anni	□ SI	□ NO
- Consenso informato	□ SI	□ NO
- Paziente ambulatoriale o DH	□ SI	□ NO
- CD4 ≥ 200 cell/µl in almeno 2 determinazioni antecedenti al To	□ SI	□ NO

CRITERI DI ESCLUSIONE

- Età >65 anni	□ SI	□ NO
- Patologia infettiva acuta in atto	□ SI	□ NO
- Antibiosi in atto o pregressa <= 7 giorni	□ SI	□ NO
- Pregressa vaccinazione con PPV23 o con PCV7	□ SI	□ NO
- Gravidanza documentata	□ SI	□ NO
- Terapia immunomodulante in atto	□ SI	□ NO
- Immunodepressione non HIV relata	□ SI	□ NO

N.B. Controindicazione all'esecuzione delle manovre diagnostiche (tampone faringeo e nasale, prelievo ematico) ed all'effettuazione di vaccinazione con PCV13, a giudizio del medico curante: □ SI □ NO

Firma Medico: ________________________________

SCHEDA CLINICO-ANAMNESTICA e FOLLOW UP

Peso _________<br>Altezza ________<br>Nadir CD4+________________<br>Stadio CDC ________________	**Fumo*** □ NO □ SI<br>**Alcool** ** □ NO □ SI<br><br>**IDUs** □ NO<br>□ SI, in atto ______________ (tipo)<br>□ SI, pregressa____________ (tipo)	**Convivenza con bambini**<br>< 3 anni □ SI □ NO<br>4-16 anni □ SI □ NO
*Se sì, specificare:<br>□ FUMATORE = almeno 100 sigarette nell'ultimo anno<br>□ EX Fumatore = almeno 100 sigarette nell'arco della vita fino a ___________<br>□ FUMO PASSIVO = > 1h/die	** Se sì, specificare:<br>□ basso ≤ 9 UA/settimana<br>□ medio 10-21UA/settimana<br>□ elevato > 21 UA/settimana	*UA = Unità Alcolica ~ 12 gr etanolo:*<br>*vino 12% 1 bicchiere = 125 ml = 12 gr*<br>*birra 4% 1 lattina = 330 ml = 11 gr*<br>*superalcolico 40% 1 bicchierino = 40 ml = 13 gr*
**FATTORI DI RISCHIO**<br>Splenectomia □ SI □ NO<br>________________________<br>Immunodepressione nonHIV<br>□ SI □ NO<br>_______________________<br>Terapia steroidea<br>□ SI □ NO<br>BPCO □ SI □ NO<br>Asma □ SI □ NO<br>Diabete □ SI □ NO<br>Cirrosi □ SI □ NO<br>Epatite cronica □ SI □ NO<br>HBV<br>HCV<br>HDV<br>Altra causa________________<br>IRC □ SI □ NO<br>Neoplasia □ SI □ NO<br>__________________________<br>Patologie cardiovascolari<br>□ SI □ NO<br>__________________________<br>Altro<br>__________________________	**Pregresse infezioni pneumococciche invasive:**<br>□ SI □ NO<br>Descrizione e data:	**Ospedalizzazione nei 12 mesi precedenti:**<br>□ SI □ NO<br>Descrizione e data:<br><br>**Antibiosi nei 6 mesi precedenti:**<br>□ SI □ NO<br>Descrizione e data:
Note		
**Allergie/controindicazioni alla vaccinazione con PCV13:**		
**T0**<br>**DATA:___________________**	**Tampone nasofaringeo** (codice identificativo) ______________________<br>**Siero** (codice identificativo)_______________________________________<br>**Dati clinici rilevanti:**<br>Test di gravidanza (donne in età fertile)	

	**CD4+:________________**<br>**Viremia:_______________**<br>**Regime HAART: ____**<br>**____**<br>**Cotrimossazolo** □ NO □ SI________________________________<br>**Vaccinazione Prevenar-13 (0,5 ml):**<br>**lotto**<br>**scadenza**
**T4**<br>**DATA:__________________**	**Tampone nasofaringeo** (codice identificativo) _________________________<br>**Siero** (codice identificativo) ______________________________________<br>**Dati clinici rilevanti:**<br><br>**CD4+:________________**<br>**Viremia:_______________**<br>**Regime HAART: ____**<br>**____**<br>**Cotrimossazolo** □ NO □ SI________________________________
**T8**<br>**DATA:_________________**	**Tampone nasofaringeo** (codice identificativo) _______________________<br>**Siero** (codice identificativo) ____________________________________<br>**Dati clinici rilevanti**:<br>Test di gravidanza (donne in età fertile)

	**CD4+:________________**<br>**Viremia:_______________**<br>**Regime HAART: ____**<br>____<br>**Cotrimossazolo** □ NO □ SI________________________________<br><br>**Vaccinazione Prevenar-13 (0,5 ml):**<br>**lotto**<br>**scadenza**
**T24**<br>**DATA:__________________**	**Tampone nasofaringeo** (codice identificativo) ______________________<br>**Siero** (codice identificativo) _______________________________________<br>**Dati clinici rilevanti:**<br><br>**CD4+:________________**<br>**Viremia:_______________**<br>**Regime HAART: ____**<br>____<br>**Cotrimossazolo** □ NO □ SI________________________________
**T48**<br>**DATA: __________________**	**Tampone nasofaringeo** (codice identificativo) _______________________<br>**Siero** (codice identificativo) _____________________________________<br>**Dati clinici rilevanti:**<br><br>**CD4+:________________**

	**Viremia:_______________**<br>**Regime HAART: ____**<br>____<br>**Cotrimossazolo** □ NO □ SI_______________________________

**Eventi e reazioni avverse (fare riferimento alla SAE)**<br><br>**T0**<br>**DATA:__________________**	A breve termine (30 minuti dalla vaccinazione)<br>□ SI □ NO<br>Note:
	A medio termine (intervista telefonica al 5° giorno postvaccinale)<br>□ SI □ NO<br>Note:
	A lungo termine<br>□ SI □ NO<br>Note:
**Eventi e reazioni avverse (fare riferimento alla SAE)**<br><br>**T8**<br>**DATA:_________________**	A breve termine (30 minuti dalla vaccinazione)<br>□ SI □ NO<br>Note:
	A medio termine (intervista telefonica al 5° giorno postvaccinale)<br>□ SI □ NO<br>Note:
	A lungo termine<br>□ SI □ NO<br>Note:

Titolo dello studio

"Studio microbiologico di isolati di *Streptococcus pneumoniae* e valutazione sierologica dei livelli anticorpali antipneumococcici in soggetti HIV positivi ed HIV negativi, con o senza pregressa immunizzazione con vaccino polisaccaridico 23-valente": codice protocollo: PNEUMO-HIV 2011

Versione del 14/11/11

Proponente

UOC Malattie Infettive Universitarie – Policlinico Le Scotte, Siena

Responsabile della struttura: Prof. Andrea De Luca

Responsabile del progetto di ricerca: Dott. Francesca Montagnani

Introduzione / Razionale

S. pneumoniae è responsabile di polmoniti, otiti medie acute, sinusiti e riacutizzazioni di bronchite cronica; un'eziologia pneumococcica è inoltre riscontrata nel 30% delle meningiti batteriche. I pazienti HIV+ risultano 10-100 volte più suscettibili alle malattie pneumococciche e soggetti ad episodi ricorrenti: *S. pneumoniae* è uno dei principali agenti di infezioni respiratorie in questa popolazione, dove rappresenta una significativa causa di morbidità e letalità.

Come intervento profilattico nei soggetti HIV+, è prevista la vaccinazione antipneumococcica con vaccino polisaccaridico 23 valente (PPV23), la cui efficacia è ben documentata nei soggetti immunocompetenti. Sebbene alcuni studi ne abbiano dimostrato l'efficacia nei pazienti HIV positivi, alcune caratteristiche della formulazione vaccinale ne mettono in discussione la validità. È da tempo dimostrato che soggetti HIV positivi con conta di CD4<500/µl sviluppano una minore risposta anticorpale post-PPV23 rispetto a soggetti sieronegativi o a pazienti HIV con minor compromissione immunologica. Inoltre nella popolazione HIV positiva, PPV23 non è in grado di indurre una risposta duratura, che non essendo legata ad una stimolazione antigenica T-dipendente, non risente neppure di effetto booster dopo rivaccinazione.

Poiché i dati sull'efficacia clinica del PPV23 non sono ancora chiari, particolarmente nella popolazione HIV positiva, appaiono necessarie ulteriori valutazioni sulle strategie vaccinali. Rispetto ai vaccini polisaccaridici, i vaccini coniugati (PCV) con carrier proteico incrementano la risposta anticorpale per induzione di risposta immune T-dipendente e la formazione di cellule B e T di memoria.

Un vaccino antipneumococcico eptavalente coniugato con il tossoide difterico CRM197 (PCV-7) è approvato in Europa dal 2001 e si è dimostrato efficace nel ridurre l'incidenza di malattie invasive da sierotipi vaccinali (4, 6B, 9V, 14, 18C, 19F, 23F), sia nei bambini che negli adulti, per effetto dell'immunità di gregge.

L'emergenza di sierotipi non vaccinali come colonizzanti e come causa di patologie invasive ha portato allo sviluppo di un'ulteriore formulazione 13-valente (PCV-13), che utilizza la stessa proteina carrier CRM197 e che copre i sette sierotipi del PCV-7 più i sierotipi 1, 3, 5, 6A, 7F e 19A. L'efficacia e la sicurezza del PCV-13 sono state dimostrate per la popolazione pediatrica, per cui la formulazione ha specifica indicazione. La commercializzazione del PCV-13, sulla base del parere favorevole dell'EMEA del settembre 2009, è stata autorizzata in tutti i Paesi Europei. Tale formulazione è stata approvata dall'AIFA per impiego in età pediatrica, in sostituzione di PCV7. Nella nuova proposta di calendario vaccinale italiano è inserito il vaccino PCV-13 e, in attesa di poter disporre di vaccini pneumococcici coniugati registrati anche per l'utilizzo nella popolazione adulta, la Società Italiana di Igiene, Medicina Preventiva e Sanità Pubblica raccomanda, previo ottenimento di consenso informato, l'utilizzo di una dose iniziale di vaccino pneumococcico coniugato 13-valente seguito a distanza di almeno 2 mesi dalla somministrazione di vaccino polisaccaridico 23-valente.

La capacità dei vaccini coniugati di indurre una risposta immunitaria di memoria crea i presupposti per una possibile maggiore efficacia nei soggetti immunodepressi. Studi sulla popolazione adulta HIV positiva hanno riportato risultati contrastanti sulla superiorità in termini di efficacia del PCV-7 rispetto al PPV23. In alcuni casi è stata evidenziata una buona protezione nei confronti di malattie invasive in adulti HIV positivi dopo somministrazione PCV-7 ed una migliore risposta anticorpale quantitativa e qualitativa nell'impiego di PCV-7 da solo o in combinazione con PPV23. Risultati contrari sono emersi da differenti analisi ed in particolare la possibile induzione di tolleranza immunitaria da parte di PPV23, potrebbe inficiare ulteriormente l'efficacia profilattica nella popolazione HIV positiva già poco responsiva alla stimolazione polisaccaridica.

L'utilizzo del PCV-13 in soggetti adulti nei primi trial in fase 1 ha dimostrato un profilo di efficacia uguale o superiore in termini di risposta anticorpale rispetto a PPV23, con un profilo di tollerabilità sovrapponibile a PCV-7. Con tali presupposti, il maggior numero di sierotipi contenuti nel PCV-13 rispetto a PCV-7 rendono molto promettente l'impiego della formulazione 13-valente nella popolazione adulta HIV positiva, in termini di efficacia nella prevenzione delle malattie pneumococciche invasive. Nella popolazione HIV, inoltre, un successivo booster di PCV-13 potrebbe aumentare la risposta anticorpale e quindi l'efficacia profilattica.

Lo studio della risposta sierologica a tale schema vaccinale, associato all'analisi della colonizzazione nasofaringea e del seguente sviluppo di patologia, può fornire un basilare supporto per la valutazione di tale strategia profilattica.

Il progetto si propone di valutare, in modo prospettico, la prevalenza della colonizzazione nasofaringea da *Streptococcus pneumoniae* e la produzione di anticorpi di classe IgG verso i differenti antigeni polisaccaridici vaccinali, con eventuale determinazione della loro capacità opsonizzante in una popolazione composta da soggetti HIV positivi in precedenza vaccinati con PPV23 negli ultimi 5 anni e da soggetti HIV negativi (indipendentemente dallo stato vaccinale **e in assenza di fattori che potrebbero influenzare la decisione del soggetto di partecipare allo studio**), correlando i dati anamnestici, clinici, sierologici e microbiologici.
Uno screening microbiologico (tampone nasofaringeo per colonizzazione da *S. pneumoniae*), sierologico (prelievo ematico per studio anticorpale) e clinico (anamnesi ed esame obiettivo) sarà effettuato al T0 in entrambi i gruppi e ripetuto a 4, 8, 24 e 48 settimane nella popolazione HIV positiva.
Tale studio di sorveglianza microbiologica e sierologica in pazienti HIV positivi con pregressa vaccinazione con PPV23 ed in pazienti HIV negativi, si configura come sottostudio a completamento dello "Studio di efficacia del vaccino antipneumococcico coniugato 13-valente in pazienti HIV positivi" (EudraCT number 2011-004518-40 ; codice protocollo PCV13-HIV2011)
La correlazione fra i dati clinico-anamnestici, sierologici e microbiologici ed il confronto fra la popolazione dello studio principale (PCV13-HIV2011) e quella del sottostudio (PNEUMO-HIV 2011) fornirà un essenziale supporto clinico-epidemiologico per la terapia e profilassi delle infezioni pneumococciche nel paziente HIV.

Obiettivi dello studio

End-point primario:

a) valutare l'entità della colonizzazione da differenti sierotipi di *S. pneumoniae* in soggetti HIV positivi ed HIV negativi in relazione al livello anticorpale al T0;
b) determinare la risposta anticorpale quantitativa e qualitativa basale in soggetti HIV positivi e HIV negativi in relazione allo stato vaccinale pregresso
c) valutare, in termini di durata, la quantità e la qualità della risposta anticorpale indotta dal PPV23 in soggetti già precedentemente vaccinati
d) definire l'effetto di pregressa vaccinazione con PPV23 sulla colonizzazione nasofaringea e sull'insorgenza di infezioni pneumococciche invasive in adulti HIV+

End-point secondario:

a) valutare la chemiosensibilità degli isolati ai differenti antibiotici e stabilire la percentuale di isolati multiresistenti.
b) valutare l'epidemiologia molecolare degli pneumococchi isolati.

Disegno dello studio

Studio multicentrico, nazionale, non commerciale, non farmacologico di tipo interventistico, in quanto e' prevista l'effettuazione di procedure diagnostiche extraroutinarie (tampone nasofaringeo, prelievo ematico)

DURATA DELLO STUDIO: 24 mesi

PRIMA FASE: 12 mesi (1 Novembre 2011 – 1 Novembre 2012) arruolamento + screening microbiologico e sierologico;
SECONDA FASE: 12 mesi (1 Novembre 2012 – 31 Ottobre 2013) completamento studio sierologico, microbiologico ed analisi dei dati.

Centri e pazienti / soggetti:

Elenco dei centri partecipanti e del numero dei pazienti/soggetti che si prevede di arruolare

Numero totale pazienti da arruolare: 50 pazienti HIV positivi precedentemente vaccinati con PPV23 da <=5 anni + 100 pazienti HIV negativi (età > 18 anni, **e in assenza di fattori che potrebbero influenzare la decisione del soggetto di partecipare allo studio**).

Numero Centri Sperimentali: 2
UOC Malattie Infettive Universitarie, AOUS, Siena
Clinica di Malattie Infettive, UCSC, Policlinico Gemelli, Roma

Numero pazienti da arruolare per ogni centro:

In base alla casistica ed ai consensi alla partecipazione allo studio ottenibili, i due Centri distribuiranno il relativo numero di arruolamenti come segue:

SIENA: fino ad un massimo di 100 pazienti, di cui minimo 0 e massimo 10 HIV positivi precedentemente vaccinati con PPV23 da <=5 anni

ROMA: fino ad un massimo di 60 pazienti, di cui minimo 40 e massimo 50 HIV positivi precedentemente vaccinati con PPV23 da <=5 anni

Criteri di inclusione

I criteri di inclusione saranno i seguenti:

- età > 18 anni
- disponibilità dal parte del paziente o del tutore legale a fornire il proprio consenso libero ed informato
- accesso alle strutture in regime ambulatoriale o di Day Hospital
  - HIV sieropositività, con pregressa vaccinazione PPV23 (≤5aa)
  - HIV sieropositività e CD4 ≥ 200 cell/µl in due determinazioni consecutive precedenti al T0

  OPPURE
  - HIV sieronegatività indipendentemente dallo stato vaccinale

Criteri di esclusione

I criteri di esclusione saranno i seguenti

- età > 65 anni
- patologia infettiva acuta in atto
- antibiosi in atto o pregressa <= 7 giorni
- vaccinazione con PPV23 pregressa da oltre 5 anni
- pregressa vaccinazione con PCV7
- gravidanza
- terapia immunomodulante in atto
- Immunodepressione non HIV relata

Procedure

Per ogni paziente saranno valutate, al momento dell'inclusione, le seguenti variabili: età, sesso; infezioni in atto o recenti, l'eventuale data della vaccinazione antipneumococcica, ospedalizzazioni nei 12 mesi precedenti, anamnesi positiva per alcolismo, abuso di sostanze stupefacenti, diabete, cirrosi, epatite cronica, bronchite cronica, neoplasie, insufficienza renale cronica o altra patologia cronica, fattori di immunodepressione non HIV relati, contatto con bambini in età prescolare o scolare, pregressa storia di infezioni pneumococciche invasive (sepsi, meningiti, polmoniti batteriemiche). Di tutti i pazienti sieropositivi per HIV verrà, inoltre, riportata la data della diagnosi di HIV, lo stadio di malattia secondo le definizioni del CDC, lo stato viro-immunologico (numero di linfociti T CD4+ circolanti e valore della viremia di HIV), le eventuali terapie e profilassi antibiotiche in corso o effettuate nei 6 mesi precedenti, variabili demografiche ed epidemiologiche, le patologie opportunistiche HIV-relate e la storia di trattamento antiretrovirale.

Screening all'arruolamento (T0):
sarà richiesto consenso informato alla partecipazione allo studio e sarà somministrato questionario clinico-anamnestico.
Verranno quindi effettuati:
- valutazione clinico-anamnestica
- tampone nasofaringeo per la ricerca di *S. pneumoniae*
- prelievo di campione sierico, crioconservazione a - 20°C per successive analisi sierologiche (dosaggio delle IgG verso i singoli antigeni polisaccaridici vaccinali ed eventuale valutazione della loro capacità opsonizzante).

I pazienti HIV negativi non saranno sottoposti ad ulteriore follow up.

I pazienti HIV+ anni proseguiranno il monitoraggio anticorpale e microbiologico a 4, 8, 24 e 48 settimane, come di seguito indicato.

T4, T8, T24 e T48 (4, 8, 24, e 48 settimane):
- valutazione clinico-anamnestica
- tampone nasofaringeo per la ricerca di *S. pneumoniae*
- prelievo di campione sierico, crioconservazione a - 20°C per successive analisi sierologiche (dosaggio delle IgG verso i singoli antigeni polisaccaridici vaccinali ed eventuale valutazione della capacità opsonizzante)

I tamponi saranno seminati su piastre di agar Columbia addizionato con il 5% di sangue di montone + colistina ed acido nalidixico, incubate a 37°C per 16-18 ore in atmosfera arricchita di CO2 al 5%.
Gli isolati saranno identificati e processati secondo metodiche classiche (caratteristiche morfologiche macro- e microscopiche, test di solubilità alla bile, sensibilità all'optochina ed agglutinazione al latice, con anticorpi diretti contro il polisaccaride capsulare), con successiva conservazione del ceppo a -80° C in brodo Wilkins-Chalgren , con il 20% di glicerolo, fino alle successive analisi.
Per evidenziare il possibile stato di portatore in contemporanea di due o più sierotipi, più colonie con morfologia suggestiva per *S. pneumoniae* saranno prelevate e sottoposte ad analisi.

Follow up clinico.
Un soggetto verrà considerato colonizzato da *S. pneumoniae* in caso di positività di almeno un tampone nasofaringeo. I pazienti che risulteranno colonizzati saranno seguiti per i 12 mesi successivi per rilevare l'insorgenza di eventuali infezioni invasive. Tutti i pazienti che svilupperanno un'infezione pneumococcica invasiva verranno seguiti fino alla risoluzione dell'episodio infettivo.

SECONDA FASE (studio sierologico, microbiologico ed analisi statistiche: 12 mesi)
Studio sierologico:
I campione sierici, raccolti e crioconservati a - 20°C, saranno analizzati presso l'Istituto di Malattie Infettive dell'UCSC del Policlinico Gemelli di Roma, secondo le indicazione dello WHO working group:
(http://www.who.int/biologicals/areas/vaccines/pneumo/Pneumo_final_23APRIL_2010.pdf).
Come cut-off di efficacia della risposta anticorpale si farà riferimento al valore clinicamente validato di 0.35 microgrammi/mL per ciascun antigene polisaccaridico vaccinale.

Analisi degli isolati da tampone nasofaringeo.

Tutti gli isolati di pneumococco ed il loro DNA estratto saranno raccolti presso la Clinica e Laboratorio di Malattie Infettive dell'Università di Siena per l'effettuazione delle ulteriori analisi microbiologiche:

-saggio di sensibilità in vitro dei ceppi crioconservati con metodica Kirby Bauer ed E-test per la determinazione delle CMI a penicillina, ceftriaxone
- saggio della sensibilità in vitro agli antibiotici di recente introduzione (quali quinopristina-dalfopristina, linezolid, tigeciclina)
- sierotipizzazione utilizzando antisieri dello Staten Serum Institute
- tipizzazione sierotipica tramite multiplex PCR, secondo le indicazioni riportate dal CDC (http://www.cdc.gov/ncidod/biotech/strep/pcr.htm)
- valutazione della clonalità tramite Multilocus Locus Sequenze Typing (MLST) amplificando e sequenziando sette geni “housekeeping” (*aroE, gdh, gki, recP, spi, xpt* e *ddl*), secondo protocolli già precedentemente descritti. Le sequenze ottenute saranno confrontate con quelle presenti nel database internazionale MLST (http://spneumoniae.mlst.net/), identificando i differenti sequence type (ST) ed effettuando inoltre una analisi filogenetica degli STs con identificazione dei complessi clonali (CCs) con l'ausilio del programma eBURST (http://eburst.mlst.net).
--analisi delle similitudini fenotipiche e valutazione della correlazione molecolare fra isolati da portatori e da successiva malattia invasiva
- selezione dei ceppi PNSSP di un campione significativo di isolati PSSP come comparazione ed esecuzione di:
-- amplificazione tramite PCR dei geni *pbp2b*, *pbp2x* (e *pbp1*a per i ceppi PRSP)

-- analisi dei polimorfismi di restrizione (“Restriction Fragment Lenght Polymorphism”, RFLP) con l'ausilio del software” Diversity DatabaseTM, Bio-Rad, version
2.2.0
Isolati da infezione invasiva.
Gli isolati da malattia saranno analizzati dal punto di vista microbiologico secondo quanto precedentemente riportato per gli isolati da colonizzazione.

4.3 Piano sperimentale

Procedura	Arruolamento T0[1,2]	T4[2]	T8[2]	T24[2]	T48[2]
Consenso informato	X				
Questionario clinico-anamnestico	X	X	X	X	X
Tampone nasofaringeo	X	X	X	X	X
Prelievo di campione sierico	X	X	X	X	X

[1] Soggetti HIV negativi
[2] Soggetti HIV positivi, precedentemente vaccinati con PPV23

Dimensioni del campione ed analisi statistica

Il campione di pazienti HIV positivi da arruolare nello studio principale (EudraCT number 2011-004518-40 Codice protocollo: PCV13-HIV2011) è previsto in numero di 50; dal presente sottostudio (codice protocollo PNEUMO-HIV2011) saranno arruolati i soggetti di controllo (50 pazienti HIV positivi e 100 pazienti HIV negativi).

I 50 soggetti HIV positivi, arruolati nello studio principale e mai vaccinati per pneumococco, saranno sottoposti a vaccinazione con PCV13 ed a follow up clinico, sierologico e microbiologico.
Un gruppo di 50 pazienti HIV positivi, in precedenza vaccinati con PPV23 da ≤ 5 anni, sarà sottoposto al medesimo follow up clinico, sierologico e microbiologico, senza interventi vaccinali e fungerà da gruppo di controllo.
In base ai dati di letteratura, si può stimare che - nei soggetti adulti HIV positivi - l’immunogenicità del PPV23 sia del 40% (Hung CC et al.2010); la risposta al vaccino coniugato eptavalente in bambini HIV positivi è viceversa riportata fra l’85 ed il 98% (Thanee C. 2011). La risposta attesa al vaccino coniugato 13-valente nella popolazione in studio è stimata pari al 70%.
Su tali basi, la numerosità del campione in studio e del gruppo di controllo (50+50) permette di ottenere una potenza statistica nel rilevare differenze pari all’81,5% con $p < 0,05$. Un gruppo di soggetti HIV negativi di pari numerosità (100 pazienti) servirà come controllo al T0 per valutare la percentuale di colonizzazione nasofaringea in relazione ai livelli anticorpali in una popolazione non HIV.

I dati clinico-anamnestici raccolti all'arruolamento ed ai successivi follow up, saranno integrati con i risultati microbiologici e sierologici e verranno applicate le successive analisi statistiche per l'elaborazione delle correlazioni, elaborando i dati dei pazienti del presente studio con i dati dello studio principale (EudraCT number 2011-004518-40 Codice protocollo: PCV13-HIV2011).

Per la valutazione della risposta immunologica, saranno calcolate le medie geometriche dei titoli anticorpali (GMT) al basale e ai tempi 4,8,24 e 48 settimane; i GMT saranno comparati mediante il test t di Student per campioni appaiati.
Per l'analisi statistica dei risultati, le variabili quantitative saranno testate per la distribuzione e comparate con il test di Mann-Whitney o Kruskal-Wallis. Le differenze tra proporzioni saranno determinate utilizzando il test del chi-quadro od il test di Fisher. I fattori associati a colonizzazione, infezione, antibioticoresistenza, risposta anticorpale efficiente (es. raggiungimento della soglia di IgG di >= 0.35 microgrammi/ml) saranno valutati mediante analisi di regressione logistica univariata e multivariata. L'analisi statistica sarà condotta utilizzando il programma

SPSS ver. 17.0 (SPSS,Chicago, IL).

Aspetti etici

Lo studio sarà condotto in accordo con i principi etici sanciti dalla Dichiarazione di Helsinki nella sua ultima revisione. La partecipazione allo studio sarà subordinata all'ottenimento del consenso libero e informato (allegato) e saranno salvaguardati i diritti sanciti dalla legge in materia di protezione dei dati personali (Decreto Legislativo 30/6/2003 n. 196 e successive modificazioni) delle persone sottoposte a valutazione. Ciascun gruppo collaborante dovrà sottoporre lo studio al parere del proprio Comitato Etico Locale.

Monitoraggio e valutazione della qualità dello studio

1) Raggiungimento di un numero appropriato di soggetti reclutati: un adeguato numero di campioni analizzati è il requisito necessario per l'esecuzione dello studio
2) Le analisi statistiche forniranno i livelli di significatività dei dati ottenuti
3) Successivamente all'analisi statistica, sarà possibile creare uno "score" di rischio di colonizzazione e di infezione da pneumococco in relazione ai fattori di rischio, allo stato vaccinale ed alla risposta anticorpale.

Finanziamento dello studio

Studio spontaneo no profit che non prevede pagamento degli oneri fissi al Comitato Etico
Le indagini extaroutinarie previste dalla sperimentazione saranno finanziate da fondo ministeriale ad hoc (PRIN Programmi di Ricerca Scientifica di Rilevante Interesse Nazionale, Cofinanziamento D.M. 19 marzo 2010 n°51)
Sottostudio microbiologico e sierologico PNEUMO-HIV 2011 per il centro coordinatore (AOUS), lo sperimentatore proponente, responsabile per il centro di Siena, ha richiesto la possibilità di avvalersi del meccanismo della responsabilità diretta in vigore all'interno della struttura di appartenenza.

Bibliografia

1. Musher D. M. 2005. Streptococcus pneumoniae. P. 2342-2411. In Mandell G.L., Bennet J.E., Dolin R. Principles and practice of infectious diseases (6th ed.). Churchill Livingston. N.Y

2. Cartwight K. 2002. Pneumococcal disease in western Europe: burden of disease, antibiotic resistance and management. Europ. J. Pediatr. 161:188-195.

3. Redd SC et al. 1990. The role of human immunodeficiency virus infection in pneumococcal bacteremia in San Francisco residents. J Infect Dis 162: 1012-1017.

4. Klugman KP et al. 2007. HIV and pneumococcal disease. Curr Opin Infect Dis 20:11-15.

5. Frankel et al. 1996. Invasive pneumococcal disease: clinical features, serotypes and antimicrobial resistance patterns in cases involving patient with and without human immunodeficiency virus infection. Cin Infect Dis 23:577-584

6. Rodriguez-Barradas et al. 1997. Colonization by Streptococcus pneumoniae among human immunodeficiency virus-infected adults: prevalence of antibiotic resistance, impact of immunization, and characterization by polymerase chain reaction with BOX primers of isolates from persistent S. pneumoniae carriers. J Infect Dis 175:590-597.

7. Bogaert D et al. 2004. Streptococcus pneumoniae colonisation: the key to pneumococcal disease. Lancet Infect Dis 4:144-154.

8. Penaranda M et al. 2007. Effectiveness of polysaccharide pneumococcal vaccine in HIV-infected patients: a case control study. Clin Infect Dis 45: 82-87.

9. Teshale EH, et al. Effectiveness of 23-valent polysaccharide pneumococcal vaccine on pneumonia in HIV-infected adults in the United States, 1998--2003. Vaccine. 2008;26(46):5830-5834.

10. Rodriguez-Barradas MC, et al. Antibody to capsular polysaccharides of Streptococcus pneumoniae after vaccination of human immunodeficiency virus-infected subjects with 23-valent pneumococcal vaccine. J Infect Dis. 1992. 165:553-6.

11. Kroon FP, et al. Antibodies against pneumococcal polysaccharides after vaccination in HIV-infected individuals: 5-year follow-up of antibody concentrations. Vaccine. 1999;18:524-30.
12. Ahmed F, et al. Effect of human immunodeficiency virus type 1 infection on the antibody response to a glycoprotein conjugate pneumococcal vaccine: results from a randomized trial. J Infect Dis. 1996;173:83-90.
13. Nielsen H, et al.. Rapid loss of specific antibodies after pneumococcal vaccination in patients with human immunodeficiency virus-1 infection. Scand J Infect Dis. 1998;30:597-601.
14. Tasker SA, et al.. Reimmunization with 23-valent pneumococcal vaccine for patients infected with human immunodeficiency virus type 1: clinical, immunologic, and virologic responses. Clin Infect Dis. 2002;34:813-21.
15. Rodriguez-Barradas MC, et al. IgG antibody to pneumococcal capsular polysaccharide in human immunodeficiency virus-infected subjects: persistence of antibody in responders, revaccination in nonresponders, and relationship of immunoglobulin allotype to response. J Infect Dis. 1996;173:1347-53.
16. Mond JJ, et al.. T cell-independent antigens type 2. Annu Rev Immunol. 1995;13:655-92. Review.
17. O'Brien KL, et al. Combined schedules of pneumococcal conjugate and polysaccharide vaccines: is hyporesponsiveness an issue? Lancet Infect Dis. 2007;7:597-606.
18. Onwubiko C. et al 2008. Cross-sectional study of nasopharingeal carriage of Streptococcus pneumoniae in human immunodeficiency virus-infected adults in the conjugate vaccine era. J Clin Microbiol 46:3621-3625.
19. Munoz-Almagro C. et al. Emergence of invasive pneumococcal disease caused by nonvaccine serotypes in the era of 7-valent conjugate vaccine. Clin Infect Dis 2008. 46:174-182.
20. Huang SS, et al. Post-PCV7 changes in colonizing pneumococcal serotypes in 16 Massachusetts communities, 2001 and 2004. Pediatrics. 2005;116:e408-13. Erratum in: Pediatrics. 2006;117:593-4
21. CDC. Invasive pneumococcal disease in children 5 years after conjugate vaccine introduction--eight states, 1998-2005. MMWR Morb Mortal Wkly Rep. 2008;57:144-8.
22. CDC. Direct and indirect effects of routine vaccination of children with 7-valent pneumococcal conjugate vaccine on incidence of invasive pneumococcal disease--United States, 1998-2003. MMWR Morb Mortal Wkly Rep. 2005;54:893-7.
23. Whitney CG, et al. Active Bacterial Core Surveillance of the Emerging Infections Program Network. Decline in invasive pneumococcal disease after the introduction of protein-polysaccharide conjugate vaccine. N Engl J Med. 2003;348:1737-46.
24. Pilishvili T, et al. Active Bacterial Core Surveillance/Emerging Infections Program Network. Sustained reductions in invasive pneumococcal disease in the era of conjugate vaccine. J Infect Dis. 2010;201:32-41.
25. Millar EV, et al. Effect of community-wide conjugate pneumococcal vaccine use in infancy on nasopharyngeal carriage through 3 years of age: a cross-sectional study in a high-risk population. Clin Infect Dis. 2006;43:8-15.
26. O'Brien KL, et al. Effect of pneumococcal conjugate vaccine on nasopharyngeal colonization among immunized and unimmunized children in a community-randomized trial. J Infect Dis. 2007;196:1211-20.
27. CDC. Licensure of a 13-valent pneumococcal conjugate vaccine (PCV13) and recommendations for use among children - Advisory Committee on Immunization Practices (ACIP), 2010. MMWR Morb Mortal Wkly Rep. 2010;59:258-61.
28. Esposito S, et al. Safety and immunogenicity of 13-valent pneumococcal conjugate vaccine compared to 7-valent pneumococcal conjugate vaccine given as a 3-dose series with routine vaccines in healthy infants and toddlers. Clin Vaccine Immunol. 2010 Apr 28.
29. French N, et al. A trial of a 7-valent pneumococcal conjugate vaccine in HIV-infected adults. N Engl J Med. 2010;362:812-22.
30. Kroon FP, et al. Enhanced antibody response to pneumococcal polysaccharide vaccine after prior immunization with conjugate pneumococcal vaccine in HIV-infected adults. Vaccine. 2000;19:886-94.

31. Lesprit P, et al. ANRS 114-Pneumovac Study Group. Immunological efficacy of a prime-boost pneumococcal vaccination in HIV-infected adults. AIDS. 2007;21:2425-34.

32. Peñaranda M, et al. Majorcan Pneumococcal Study Group. Conjugate and polysaccharide pneumococcal vaccines do not improve initial response of the polysaccharide vaccine in HIV-infected adults. AIDS. 2010;24:1226-8.

33. Scott DA, et al. Phase 1 trial of a 13-valent pneumococcal conjugate vaccine in healthy adults. Vaccine. 2007;25:6164-6.

34. Hung CC et al. ,A 5-year longitudinal follow-up study of serological responses to 23-valent pneumococcal polysaccharide vaccination among patients with HIV infection who received highly active antiretroviral therapy. HIV Medicine 2010; 11: 54-63.

35. **Thanee C. et al. The immunogenicity and safety of pneumococcal conjugate vaccine in human immunodeficiency virus-infected Thai children. Vaccine. 2011; 29:5886-91**

SCHEDA CLINICA **SOTTOSTUDIO**

(Codice protocollo PNEUMO-HIV2011)

SCHEDA DI ARRUOLAMENTO

CENTRO SPERIMENTALE____

ID paziente____________________________ Data di Nascita:____________________

RESIDENZA:____________________________________ Tel:______________________________

DATA di ARRUOLAMENTO __________________________

Sesso: □ M □ F Etnia: _____________________________________

CRITERI DI INCLUSIONE

7	Età > 18 anni	□ SI	□ NO
8	Consenso informato	□ SI	□ NO
9	Paziente ambulatoriale o DH	□ SI	□ NO
10	Paziente HIV positivo, con vaccinazione PPV23 ≤5aa	□ SI	□ NO
11	HIV sieropositività e CD4 ≥ 200 cell/µl	□ SI	□ NO
	OPPURE		
12	Paziente HIV negativo (gruppo di controllo)	□ SI	□ NO

CRITERI DI ESCLUSIONE

-

-	Età >65 anni	□ SI	□ NO
-	Patologia infettiva acuta in atto	□ SI	□ NO
-	Antibiosi in atto o pregressa <= 7 giorni	□ SI	□ NO
-	Pregressa vaccinazione con PPV23	□ SI	□ NO
-	Pregressa vaccinazione con PCV7	□ SI	□ NO
-	Gravidanza documentata o sospetta	□ SI	□ NO
-	Terapia immunomodulante in atto	□ SI	□ NO
-	Immunodepressione non HIV relata	□ SI	□ NO

N.B. Controindicazione all'esecuzione delle manovre diagnostiche (tampone faringeo e nasale, prelievo ematico), a giudizio del medico curante:

□ SI □ NO

Firma Medico: _________________________________

SCHEDA CLINICO-ANAMNESTICA e FOLLOW UP

Peso _________<br>Altezza _______<br>*Nadir CD4+*___________<br>*Stadio CDC* ___________<br>***oppure***<br>*Data test HIV NEGATIVO*<br>_____________________	**Fumo*** □ NO □ SI<br>**Alcool** ** □ NO □ SI<br><br>**IDUs** □ NO<br>□ SI, in atto _____________ (tipo)<br>□ SI, pregressa___________ (tipo)	**Convivenza con bambini**<br>< 3 anni □ SI □ NO<br>4-16 anni □ SI □ NO
*Se sì, specificare:<br>□ FUMATORE = almeno 100 sigarette nell'ultimo anno<br>□ EX Fumatore = almeno 100 sigarette nell'arco della vita fino a __________<br>□ FUMO PASSIVO = > 1h/die	** Se sì, specificare:<br>□ basso ≤ 9 UA/settimana<br>□ medio 10-21UA/settimana<br>□ elevato > 21 UA/settimana	*UA = Unità Alcolica ~ 12 gr etanolo:*<br>*vino 12% 1 bicchiere = 125 ml = 12 gr*<br>*birra 4% 1 lattina = 330 ml = 11 gr*<br>*superalcolico 40% 1 bicchierino = 40 ml = 13 gr*
**FATTORI DI RISCHIO**<br>Splenectomia □ SI □ NO<br>_____________________<br>Immunodepressione nonHIV<br>□ SI □ NO<br>_____________________<br>Terapia steroidea<br>□ SI □ NO<br>BPCO □ SI □ NO<br>Asma □ SI □ NO<br>Diabete □ SI □ NO<br>Cirrosi □ SI □ NO<br>Epatite cronica □ SI □ NO<br>HBV<br>HCV<br>HDV<br>Altra causa<br>IRC □ SI □ NO<br>Neoplasia □ SI □ NO<br>____________________<br>Patologie cardiovascolari<br>□ SI □ NO<br>____________________<br>Altro:<br>_____________________	**Data vaccinazione e richiami PPV23:**<br>______________________<br>______________________<br>______________________<br>______________________<br>______________________<br><br>**Pregresse infezioni pneumococciche invasive:**<br>□ SI □ NO<br>Descrizione e data:	**Ospedalizzazione nei 12 mesi precedenti:**<br>□ SI □ NO<br>Descrizione e data:<br><br>**Antibiosi nei 6 mesi precedenti:**<br>□ SI □ NO<br>Descrizione e data:
**Note**	**Allergie**	

**T0** ***(HIV+ e HIV-)***<br><br>**DATA:__________________**	**Tampone nasofaringeo** (codice identificativo)<br>**Siero** (codice identificativo)<br>**Dati clinici rilevanti:**<br><br><br><br>***Per HIV+:***<br>**CD4+:________________**<br>**Viremia:_______________**<br>**Regime HAART: ____**<br>**____**<br>**Cotrimossazolo** □ NO □ SI________________________________

**T4** ***(solo HIV+)***<br><br>**DATA:__________________**	**Tampone nasofaringeo** (codice identificativo)<br>**Siero** (codice identificativo)<br>**Dati clinici rilevanti:**<br><br>**CD4+:________________**<br>**Viremia:_______________**<br>**Regime HAART: ____**<br>____<br>**Cotrimossazolo** □ NO □ SI________________________________
**T8** ***(solo HIV+)***<br><br>**DATA:_________________**	**Tampone nasofaringeo** (codice identificativo)<br>**Siero** (codice identificativo)<br>**Dati clinici rilevanti**:<br><br>**CD4+:________________**<br>**Viremia:_______________**<br>**Regime HAART: ____**<br>____<br>**Cotrimossazolo** □ NO □ SI________________________________

**T24***(solo HIV+)*<br><br>**DATA:___________________**	**Tampone nasofaringeo** (codice identificativo)<br>**Siero** (codice identificativo)<br>**Dati clinici rilevanti:**<br><br><br><br>**CD4+:_________________**<br>**Viremia:________________**<br>**Regime HAART: ____**<br>____<br>**Cotrimossazolo** □ NO □ SI__________________________________
**T48** ***(solo HIV+)***<br><br>**DATA: ___________________**	**Tampone nasofaringeo** (codice identificativo)<br>**Siero** (codice identificativo)<br>**Dati clinici rilevanti:**<br><br><br><br>**CD4+:_________________**<br>**Viremia:________________**<br>**Regime HAART: ____**<br>____<br>**Cotrimossazolo** □ NO □ SI__________________________________
