# Supplementary material for: Immunogenicity and Safety of the 13-Valent Pneumococcal Conjugate Vaccine versus the 23-Valent Polysaccharide Vaccine in Unvaccinated HIV-Infected Adults: A Pilot, Prospective Controlled Study
Source: PLoS One. 2016 Jun 3;11(6):e0156523. doi: 10.1371/journal.pone.0156523 (PMC4892598; doi:10.1371/journal.pone.0156523)
Supplement: S2 Protocol — (DOC) [file pone.0156523.s005.doc]

# TITLE

# Serological Response to Antipneumococcal Vaccination and Impact on *Streptococcus Pneumoniae* Nasal Carriage in HIV Adults

**Eudract: 2011-004518-40**

### Protocol code : PCV13-HIV2011

**PRINCIPAL INVESTIGATOR:** Francesca Montagnani, MD, PhD

**SITE:** University Division of Infectious Diseases, University of Siena,

Siena, Italy

**COLLABORATOR:** Simona Di Giambenedetto , MD

**SITE:** Institute of Clinical Infectious Diseases, Catholic University of the Sacred Heart , Rome, Italy

**RATIONALE**

*S. pneumoniae* is frequently isolated from nasal swabs of healthy subjects, but it can also cause severe diseases (pneumonia, bacteraemia, meningitis and sepsis).HIV-infected subjects are more sensitive to invasive diseases and recurrent infection than the general population. Nasal carriage is the main pathogenetic feature for invasive disease: bacteraemia is more frequent in carriers, HIV+ patients are constantly colonized by the same pneumococcal strain and their nasopharyngeal isolates have features similar to subsequent invasive strains. A 23-valent polysaccharide vaccine (PPV23) has long been available and recommended in the HIV+ population as prophylaxis for invasive disease. Studies regarding efficacy of PPV23 in HIV+ are controversial and highlight that immune response induced by PPV23 in HIV+ is poor and an hyporesponsiveness to repeated polysaccaridic antigens stimulation can occur. Moreover, PPV23 seems not to affect pneumococcal carriage status and could lead to emergence of non-vaccine serotypes. The conjugation of pneumococcal capsular polysaccharides to carrier proteins results in an improved T-cell dependent immune response, characterized by increased antibody concentrations and induction of T and B memory cells, with a demonstrated higher efficacy in children. A heptavalent vaccine conjugated with diphtheria toxoid (PCV7) is approved in Europe since 2001 and is effective in reducing incidence of invasive disease by vaccine serotypes (4, 6B, 9V, 14, 18C, 19F, 23F), in both children and adults, due to effect of herd immunity. A PCV13 formulation has recently been developed, covering PCV7 serotypes plus 1, 3, 5, 6A, 7F and 19A. PCV13 revealed the same safety profile as PCV7 in pediatric patients, that are the main target of conjugate vaccines licensure. Some trials showed a better antibody response in terms of quantity and quality in HIV + adults by using PCV7 as compared to PPV23. However these data were not unequivocally confirmed in further studies on the use of PCV7 alone or in combination with PPV23. The first trials of PCV13 use in adults showed the same or even better response compared to PPV23, with a safety and tolerability similar to PCV7. PCV13 in HIV+ adults is a promising candidate prophylactic measure for pneumococcal infections.

The purpose of this study is to evaluate serological response and prevalence of nasopharyngeal colonization by *S. pneumoniae* in HIV+ non-hospitalized adults, following vaccination with 2 doses of PCV13.

**Biological: pneumococcal conjugate 13 valent vaccine**

Pneumococcal polysaccharide conjugate vaccine(13-valent adsorbed) conjugated to CRM197 carrier protein and adsorbed on aluminum phosphate (0.125 mg of aluminum). Prevenar 13 is administered in two doses, each of 0.5 ml, with an interval of 2 months, injected intramuscularly in the deltoid muscle of the arm.

**Pharmaceutical form**: suspension for injection. Dosage: 0.5 ml, containing 2.2 g of polysaccharide for serotypes 1, 3, 4, 5, 6A, 7F, 9V, 14, 18C, 19A, 19F, 23F, and 4.4 micrograms for serotype 6B.

**Primary Outcome Measures:**

- Define the serological response after 2 doses of PCV13 vaccine. Measure of serological response after 2 doses of PCV13 vaccine (booster dose after 8 weeks) in HIV+ adults.
- Determine the rate of nasopharyngeal colonization by different pneumococcal serotypes in HIV-positive adults, in relation to baseline antibody titers at T0

**Secondary Outcome Measures:**

- Pneumococcal chemosusceptibility: Number and percentages of antibiotic resistant (including multiresistant) strains
- Molecular epidemiology : Molecular typing combining PFGE (Pulsed Field Gel Electrophoresis), MLST (MultiLocus Sequence Typing) and PCR analysis of bacterial isolates

**STUDY DESIGN**

This is a phase III, prospective, multicenter, pilot controlled study enrolling HIV-infected outpatients, aged 18–65 years with CD4 counts ≥200 cells/µL, pneumococcal vaccine-naïve.

**Study duration: 24 months**

**Phase I:** 12 months (November, 1st 2011 – November, 1st 2012) enrolling + microbiological and serological screening.

**Phase I:** 12 months (November, 1st 2012 – October , 31st 2013) serological and microbliological analyses and data elaboration.

**Inclusion Criteria:**

- > 18 years old
- obtained informed consent
- outpatient
- CD4 ≥200 cells/µl in the last two evaluations before T0

**Exclusion Criteria:**

- > 65 years old
- presence of acute infectious disease
- antibiotic therapy (ongoing or in the previous <= 7 days)
- previous PPV23 or PCV7 vaccination
- Pregnancy
- Current immunomodulatory therapy
- Immunosuppression not HIV related

**Sample size and study group selection**

Applying these criteria, 50 HIV-infected adult outpatients who had never been vaccinated with any pneumococcal vaccine will be prospectively enrolled at two Infectious Diseases Clinical Centers in Central Italy (University Division of Infectious Diseases in Siena and Institute of Clinical Infectious Diseases, Catholic University of the Sacred Heart in Rome). All subjects will be required to sign a written informed consent before enrolling in the study.

At baseline (day 0, time of first vaccine dose administration), demographic, clinical and laboratory characteristics will be collected by patients interview.

At baseline, the enrolled patients will received two doses of the 13-valent vaccine (PCV13 Prevenar13®, Wyeth Vaccines) eight weeks apart.

Follow-up visits will be scheduled at 8 (time of second PCV13 dose), 24 and 48 weeks. At baseline and at each visit clinical and anamnestic data from patients will be recorded; peripheral venous blood samples will be collected to assess humoral immune response against PCV13 serotype; nasopharyngeal swabs will be collected, in order to investigate the *S. pneumonia* presence. CD4+ T cell count and plasma HIV-RNA will be also monitored.

After each vaccine administration, short- (30 minutes), medium- (≤5 days) and long-term adverse reactions will be monitored.

**STUDY METHODOLOGY**

**Immunological evaluations**

Sera collected from all patients will be stored at -20°C until use and they will be tested at Institute of Clinical Infectious Diseases, Catholic University of the Sacred Heart, Rome. Quantitation of specific IgG levels for 13 PCV13 serotypes (1, 3, 4, 5, 6A, 6B, 7F, 9V, 14, 18C, 19F, 19A and 23F) will be performed on sera, by an ELISA method, according to the World Health Organization (WHO) procedure; IgG concentrations will be expressed as geometric mean concentration (GMC).

The generally used threshold of 0.35µg/mL (accepted by the WHO for assessment of vaccine efficacy against IPD) it will be chosen as cut-offs for defining seroprotection levels.

**Microbiological evaluations**

All the isolated strains of *S. pneumoniae* and their genomic DNA will be collected at University Division of Infectious Diseases, Siena.

Swabs will plated on Columbia CNA + 5% blood agar then incubated at 37°C for 16-18 hours in CO2 enriched atmosphere. Bacterial isolates will be identified and processed through standard procedures: small, α-hemolythic, “draughtsman” colonies or small, mucous, α-hemolythic colonies will be confirmed through Optochin-disk testing, bile susceptibility testing and latex agglutination test. Isolates will be further analyzed through molecular typing as described below. Isolates that will not be immediately tested will be freezed at -80°C in Wilkins-Chalgren broth + 20% glycerol for further analyses.

**Clinical follow-up**

Subjects will be considered colonized by S. pneumoniae with at least one positive nasopharingeal swab. Colonized patients will be followed for the next 12 months so to observe potential invasive infections. Potential simultaneous colonization by two or more serotypes will be highlighted through the analysis of isolated colonies.

**Analysis of isolates from nasopharingeal swab**

All the isolated strains of S. pneumoniae will be collected and subdued to antibiotic susceptibility testing following CLSI guidelines (Kirby-bauer disk diffusion tests, MIC susceptibility testing), and results will be interpreted following CLSI breakpoints. Susceptibility to recently introduced antibiotics (tigecycline, linezolid, quinupristin-dalfopristin) will be assessed. Strains will be also serotyped using sera obtained from SSI (Statens Serum Institut).

S. pneumoniae isolates will also be subdued to genotypic analysis. Genomic DNA from a fresh colture will be extracted with High Pure PCR Template Preparation Kit (Roche), then a multiplex PCR reaction will be performed so to deduce capsular serotype, as indicated by CDC (<http://www.cdc.gov/streplab/downloads/pcr-oligonucleotide-primers.pdf>). Clonalty of isolates will also be assessed through multi locus sequence typing (MLST), amplifying and sequencing seven housekeeping genes (aroE, gdh, gki, recP, spi, xpt and ddl) as indicated by MLST website ([http://www.mlst.net](http://www.mlst.net/)). PCR products will be purified, sequenced and results compared to international databases (<http://spneumoniae.mlst.net/>), allowing identification of different Sequence Types (STs) or performing phylogenic analysis of different STs through eBURST open-source software ([http://eburst.mlst.net](http://eburst.mlst.net/)).

We will also focus on other features of S. pneumoniae, such as the evaluation of molecular and phenotypic features amid isolates retrieved from invasive diseases. Moreover, a selection of Penicillin resistant S. pneumoniae (PNSSP) will be made and comparison against PSSP will be performed trough:

- PCR amplification of

-- PCR amplification of pbp2b, pbp2x genes (and pbp1a for PRSP strains)

-- restriction fragments length polymorphisms (RFLP) through Diversity DatabaseTM, Bio-Rad, version 2.2.0 software.



**STATISTIC ANALYSIS**

Clinical and anamnestic data collected at enrolment and at each follow-up visit, will be integrated with microbiological and serological results and correlation analyses will be performed.

Further statistical analyses will be carried out considering the results from this study and from the sub-study (protocol code PNEUMO-HIV 2011).

To evaluate the immunological response, the geometric mean concentrations of IgG (GMCs) at baseline, 4,8,24 e 48 week will be calculated. The GMCs will be compared by Student's' t Test (For Paired Samples). The quantitative variable will be tested for normal distribution and compared by

Mann-Whitney or Kruskal-Wallis test.

Differences in proportion will be determined by chi squared test or fischer exact test.

Regression or logistic analyses (univariate and multivariate) will asses the association with colonization, infection, antibiotic resistance, and an efficient immune response (e.g. ≥0.35μg/mL).

Analyses were performed using the SPSS software package (version 17.0 Chicago, IL).

# TITLE

**Microbiological Study of *Streptococcus Pneumoniae* Isolates and Evaluation of Serological Response in Terms of Antipneumococcal Antibodies in HIV-positive patients and HIV-negative Subjects Regardless Previous PPV23 Pneumococcal Vaccination**

**Protocol code:** **PNEUMO-HIV 2011**

**PRINCIPAL INVESTIGATOR:** Francesca Montagnani, MD, PhD

**SITE:** University Division of Infectious Diseases, University of Siena,

Siena, Italy

**COLLABORATOR:** Simona Di Giambenedetto , MD

**SITE:** Institute of Clinical Infectious Diseases, Catholic University of the Sacred Heart , Rome, Italy

**RATIONALE**

*S. pneumoniae* is frequently isolated from nasal swabs of healthy subjects, but it can also cause severe diseases (pneumonia, bacteraemia, meningitis and sepsis).HIV-infected subjects are more sensitive to invasive diseases and recurrent infection than the general population. Nasal carriage is the main pathogenetic feature for invasive disease: bacteraemia is more frequent in carriers, HIV+ patients are constantly colonized by the same pneumococcal strain and their nasopharyngeal isolates have features similar to subsequent invasive strains. A 23-valent polysaccharide vaccine (PPV23) has long been available and recommended in the HIV+ population as prophylaxis for invasive disease. Studies regarding efficacy of PPV23 in HIV+ are controversial and highlight that immune response induced by PPV23 in HIV+ is poor and an hyporesponsiveness to repeated polysaccaridic antigens stimulation can occur. Moreover, PPV23 seems not to affect pneumococcal carriage status and could lead to emergence of non-vaccine serotypes. The conjugation of pneumococcal capsular polysaccharides to carrier proteins results in an improved T-cell dependent immune response, characterized by increased antibody concentrations and induction of T and B memory cells, with a demonstrated higher efficacy in children. A heptavalent vaccine conjugated with diphtheria toxoid (PCV7) is approved in Europe since 2001 and is effective in reducing incidence of invasive disease by vaccine serotypes (4, 6B, 9V, 14, 18C, 19F, 23F), in both children and adults, due to effect of herd immunity. A PCV13 formulation has recently been developed, covering PCV7 serotypes plus 1, 3, 5, 6A, 7F and 19A. PCV13 revealed the same safety profile as PCV7 in pediatric patients, that are the main target of conjugate vaccines licensure. Some trials showed a better antibody response in terms of quantity and quality in HIV + adults by using PCV7 as compared to PPV23. However these data were not unequivocally confirmed in further studies on the use of PCV7 alone or in combination with PPV23. The first trials of PCV13 use in adults showed the same or even better response compared to PPV23, with a safety and tolerability similar to PCV7. PCV13 in HIV+ adults is a promising candidate prophylactic measure for pneumococcal infections.

The purpose of this study is to evaluate serological response and prevalence of nasopharyngeal colonization by *S. pneumoniae* in HIV-positive non-hospitalized adults, following vaccination with one dose of PPV23, and in unvaccinated HIV-negative subjects.

To completion of the study “Serological Response to Antipneumococcal Vaccination and Impact on *Streptococcus Pneumoniae* Nasal Carriage in HIV Adults” (protocol code PCV13-HIV2011 EudraCT number 2011-004518-40) the present study will be conducted simultaneously, for a microbiological and serological monitoring in HIV-positive adults with previous PPV23 vaccination and in HIV-negative subjects, regardless their vaccination status. Correlation analyses between clinical-anamestic data, immune response and microbiological results and the comparison between the populations from the main study (EudraCT number 2011-004518-40 ; protocol code PCV13-HIV2011) and from this sub-study (protocol code PNEUMO-HIV 2011) will provide essential clinical and epidemiologic findings useful to choice the optimal therapy and prophylaxis to prevent the pneumococcal diseases in HIV-positive adults.

**Primary Outcome Measures:**

- Determine the rate of nasal colonization by different pneumococcal serotypes in HIV-positive and HIV-negative adults, in relation to baseline antibody titers at T0;
- Determine the basal serological response (qualitative and quantitative) in HIV-positive and HIV-negative adults;
- Define serological response after one dose of PPV23 vaccine in HIV+ adults;
- Evaluate the effect of one dose of PPV23 vaccine in terms of nasal pneumococcal carriage and occurrence of invasive pneumococcal diseases;

**Secondary Outcome Measures:**

- Pneumococcal chemosusceptibility: Number and percentages of antibiotic resistant (including multiresistant) strains
- Molecular epidemiology : Molecular typing combining PFGE (Pulsed Field Gel Electrophoresis), MLST (MultiLocus Sequence Typing) and PCR analysis of bacterial isolates

**STUDY DESIGN**

This is a prospective, multicenter, pilot controlled study enrolling HIV-infected outpatients, aged 18–65 years with CD4 counts ≥200 cells/µL, previously vaccinated , and unvaccinated HIV-negative subjects as a control,

**Study duration: 24 months**

**Phase I:** 12 months (November, 1st 2011 – November, 1st 2012) enrolling + microbiological and serological screening.

**Phase I:** 12 months (November, 1st 2012 – October , 31st 2013) serological and microbliological analyses and data elaboration.

**Inclusion Criteria:**

- > 18 years old
- obtained informed consent
- outpatient
- HIV with previous PPV23 vaccinatio (≤5years)
- HIV with CD4 ≥200 cells/µl in the last two evaluations before T0
- HIV negative regardless vaccination status

**Exclusion Criteria:**

- > 65 years old
- presence of acute infectious disease
- antibiotic therapy (ongoing or in the previous <= 7 days)
- previous PPV23 or PCV7 vaccination
- Pregnancy
- Current immunomodulatory therapy
- Immunosuppression not HIV related

**Sample size and study group selection**

Applying these criteria, 50 HIV-infected adult outpatients and 100 subjects HIV negative will be prospectively enrolled at two Infectious Diseases Clinical Centers in Central Italy (University Division of Infectious Diseases in Siena and Institute of Clinical Infectious Diseases, Catholic University of the Sacred Heart in Rome). All subjects will be required to sign a written informed consent before enrolling in the study.

At baseline, demographic, clinical and laboratory characteristics will be collected by patients interview.

At baseline, the enrolled HIV-positive patients will received one dose of the 23-valent vaccine (PPV 23); follow-up visits will be scheduled at 8 (time of second PCV13 dose), 24 and 48 weeks.

At baseline and at each visit clinical and anamnestic data from patients will be recorded; peripheral venous blood samples will be collected to assess humoral immune response against PPV23 serotype; nasopharyngeal swabs will be collected, in order to investigate the *S. pneumonia* presence. CD4+ T cell count and plasma HIV-RNA will be also monitored.

After each vaccine administration, short- (30 minutes), medium- (≤5 days) and long-term adverse reactions will be monitored.

Oppositely, HIV negative subjects will not receive any vaccination; only at baseline clinical and anamnestic data will be recorded; peripheral venous blood samples will be collected to assess humoral immune response against PPV23 serotype; nasopharyngeal swabs will be collected, in order to investigate the *S. pneumonia* presence.

**STUDY METHODOLOGY**

**Immunological evaluations**

Sera collected from all patients will be stored at -20°C until use and they will be tested at Institute of Clinical Infectious Diseases, Catholic University of the Sacred Heart, Rome. Quantitation of specific IgG levels for 12 serotypes shared by PCV13 and PPV23 (1, 3, 4, 5, 6B, 6B, 7F, 9V, 14, 18C, 19F, 19A and 23F) and for the additional serotype contained only in PCV13 (6A) will be performed on sera, by an ELISA method, according to the World Health Organization (WHO) procedure; IgG concentrations will be expressed as geometric mean concentration (GMC).

The generally used threshold of 0.35µg/mL (accepted by the WHO for assessment of vaccine efficacy against IPD) it will be chosen as cut-offs for defining seroprotection levels.

**Microbiological evaluations**

All the isolated strains of *S. pneumoniae* and their genomic DNA will be collected at University Division of Infectious Diseases, Siena.

Swabs will plated on Columbia CNA + 5% blood agar then incubated at 37°C for 16-18 hours in CO2 enriched atmosphere. Bacterial isolates will be identified and processed through standard procedures: small, α-hemolythic, “draughtsman” colonies or small, mucous, α-hemolythic colonies will be confirmed through Optochin-disk testing, bile susceptibility testing and latex agglutination test. Isolates will be further analyzed through molecular typing as described below. Isolates that will not be immediately tested will be freezed at -80°C in Wilkins-Chalgren broth + 20% glycerol for further analyses.

**Clinical follow-up**

Subjects will be considered colonized by S. pneumoniae with at least one positive nasopharingeal swab. Colonized patients will be followed for the next 12 months so to observe potential invasive infections. Potential simultaneous colonization by two or more serotypes will be highlighted through the analysis of isolated colonies.

**Analysis of isolates from nasopharingeal swab**

All the isolated strains of S. pneumoniae will be collected and subdued to antibiotic susceptibility testing following CLSI guidelines (Kirby-bauer disk diffusion tests, MIC susceptibility testing), and results will be interpreted following CLSI breakpoints. Susceptibility to recently introduced antibiotics (tigecycline, linezolid, quinupristin-dalfopristin) will be assessed. Strains will be also serotyped using sera obtained from SSI (Statens Serum Institut).

S. pneumoniae isolates will also be subdued to genotypic analysis. Genomic DNA from a fresh colture will be extracted with High Pure PCR Template Preparation Kit (Roche), then a multiplex PCR reaction will be performed so to deduce capsular serotype, as indicated by CDC (<http://www.cdc.gov/streplab/downloads/pcr-oligonucleotide-primers.pdf>). Clonalty of isolates will also be assessed through multi locus sequence typing (MLST), amplifying and sequencing seven housekeeping genes (aroE, gdh, gki, recP, spi, xpt and ddl) as indicated by MLST website ([http://www.mlst.net](http://www.mlst.net/)). PCR products will be purified, sequenced and results compared to international databases (<http://spneumoniae.mlst.net/>), allowing identification of different Sequence Types (STs) or performing phylogenic analysis of different STs through eBURST open-source software ([http://eburst.mlst.net](http://eburst.mlst.net/)).

We will also focus on other features of S. pneumoniae, such as the evaluation of molecular and phenotypic features amid isolates retrieved from invasive diseases. Moreover, a selection of Penicillin resistant S. pneumoniae (PNSSP) will be made and comparison against PSSP will be performed trough:

- PCR amplification of

-- PCR amplification of pbp2b, pbp2x genes (and pbp1a for PRSP strains)

-- restriction fragments length polymorphisms (RFLP) through Diversity DatabaseTM, Bio-Rad, version 2.2.0 software.



**STATISTIC ANALYSIS**

Clinical and anamnestic data collected at enrolment and at each follow-up visit, will be integrated with microbiological and serological results and correlation analyses will be performed. Further statistical analyses will be carried out considering the results from this study and from the main study (protocol code PCV13-HIV2011).

To evaluate the immunological response, the geometric mean concentrations of IgG (GMCs) at baseline, 4,8,24 e 48 week will be calculated. The GMCs will be compared by Student's' t Test (For Paired Samples). The quantitative variable will be tested for normal distribution and compared by

Mann-Whitney or Kruskal-Wallis test.

Differences in proportion will be determined by chi squared test or fischer exact test.

Regression or logistic analyses (univariate and multivariate) will asses the association with colonization, infection, antibiotic resistance, and an efficient immune response (e.g. ≥0.35μg/mL).

Analyses were performed using the SPSS software package (version 17.0 Chicago, IL).

**BIBLIOGRAFY**

1. Musher D. M. 2005. *Streptococcus pneumoniae*. P. 2342-2411. In Mandell G.L., Bennet J.E., Dolin R. Principles and practice of infectious diseases (6th ed.). Churchill Livingston. N.Y

2. Cartwight K. 2002. Pneumococcal disease in western Europe: burden of disease, antibiotic resistance and management. Europ. J. Pediatr. 161:188-195.

3. Redd SC et al. 1990. The role of human immunodeficiency virus infection in pneumococcal bacteremia in San Francisco residents. J Infect Dis 162: 1012-1017.

4. Klugman KP et al. 2007. HIV and pneumococcal disease. Curr Opin Infect Dis 20:11-15.

5. Frankel et al. 1996. Invasive pneumococcal disease: clinical features, serotypes and antimicrobial resistance patterns in cases involving patient with and without human immunodeficiency virus infection. Cin Infect Dis 23:577-584

6. Rodriguez-Barradas et al. 1997. Colonization by *Streptococcus pneumoniae* among human immunodeficiency virus-infected adults: prevalence of antibiotic resistance, impact of immunization, and characterization by polymerase chain reaction with BOX primers of isolates from persistent *S. pneumoniae* carriers. J Infect Dis 175:590-597.

7. Bogaert D et al. 2004. *Streptococcus pneumoniae* colonisation: the key to pneumococcal disease. Lancet Infect Dis 4:144-154.

8. Penaranda M et al. 2007. Effectiveness of polysaccharide pneumococcal vaccine in HIV-infected patients: a case control study. Clin Infect Dis 45: 82-87.

9. Teshale EH, et al. Effectiveness of 23-valent polysaccharide pneumococcal vaccine on pneumonia in HIV-infected adults in the United States, 1998--2003. Vaccine. 2008;26(46):5830-5834.

10. Rodriguez-Barradas MC, et al. Antibody to capsular polysaccharides of *Streptococcus pneumoniae* after vaccination of human immunodeficiency virus-infected subjects with 23-valent pneumococcal vaccine. J Infect Dis. 1992. 165:553-6.

11. Kroon FP, et al. Antibodies against pneumococcal polysaccharides after vaccination in HIV-infected individuals: 5-year follow-up of antibody concentrations. Vaccine. 1999;18:524-30.

12. Ahmed F, et al. Effect of human immunodeficiency virus type 1 infection on the antibody response to a glycoprotein conjugate pneumococcal vaccine: results from a randomized trial. J Infect Dis. 1996;173:83-90.

13. Nielsen H, et al.. Rapid loss of specific antibodies after pneumococcal vaccination in patients with human immunodeficiency virus-1 infection. Scand J Infect Dis. 1998;30:597-601.

14. Tasker SA, et al.. Reimmunization with 23-valent pneumococcal vaccine for patients infected with human immunodeficiency virus type 1: clinical, immunologic, and virologic responses. Clin Infect Dis. 2002;34:813-21.

15. Rodriguez-Barradas MC, et al. IgG antibody to pneumococcal capsular polysaccharide in human immunodeficiency virus-infected subjects: persistence of antibody in responders, revaccination in nonresponders, and relationship of immunoglobulin allotype to response. J Infect Dis. 1996;173:1347-53.

16. Mond JJ, et al.. T cell-independent antigens type 2. Annu Rev Immunol. 1995;13:655-92. Review.

17. O'Brien KL, et al. Combined schedules of pneumococcal conjugate and polysaccharide vaccines: is hyporesponsiveness an issue? Lancet Infect Dis. 2007;7:597-606.

18. Onwubiko C. et al 2008. Cross-sectional study of nasopharingeal carriage of *Streptococcus pneumoniae* in human immunodeficiency virus-infected adults in the conjugate vaccine era. J Clin Microbiol 46:3621-3625.

19. Munoz-Almagro C. et al. Emergence of invasive pneumococcal disease caused by nonvaccine serotypes in the era of 7-valent conjugate vaccine. Clin Infect Dis 2008. 46:174-182.

20. Huang SS, et al. Post-PCV7 changes in colonizing pneumococcal serotypes in 16 Massachusetts communities, 2001 and 2004. Pediatrics. 2005;116:e408-13. Erratum in: Pediatrics. 2006;117:593-4

21. CDC. Invasive pneumococcal disease in children 5 years after conjugate vaccine introduction--eight states, 1998-2005. MMWR Morb Mortal Wkly Rep. 2008;57:144-8.

22. CDC. Direct and indirect effects of routine vaccination of children with 7-valent pneumococcal conjugate vaccine on incidence of invasive pneumococcal disease--United States, 1998-2003. MMWR Morb Mortal Wkly Rep. 2005;54:893-7.

23. Whitney CG, et al. Active Bacterial Core Surveillance of the Emerging Infections Program Network. Decline in invasive pneumococcal disease after the introduction of protein-polysaccharide conjugate vaccine. N Engl J Med. 2003;348:1737-46.

24. Pilishvili T, et al. Active Bacterial Core Surveillance/Emerging Infections Program Network. Sustained reductions in invasive pneumococcal disease in the era of conjugate vaccine. J Infect Dis. 2010;201:32-41.

25. Millar EV, et al. Effect of community-wide conjugate pneumococcal vaccine use in infancy on nasopharyngeal carriage through 3 years of age: a cross-sectional study in a high-risk population. Clin Infect Dis. 2006;43:8-15.

26. O'Brien KL, et al. Effect of pneumococcal conjugate vaccine on nasopharyngeal colonization among immunized and unimmunized children in a community-randomized trial. J Infect Dis. 2007;196:1211-20.

27. CDC. Licensure of a 13-valent pneumococcal conjugate vaccine (PCV13) and recommendations for use among children - Advisory Committee on Immunization Practices (ACIP), 2010. MMWR Morb Mortal Wkly Rep. 2010;59:258-61.

28. Esposito S, et al. Safety and immunogenicity of 13-valent pneumococcal conjugate vaccine compared to 7-valent pneumococcal conjugate vaccine given as a 3-dose series with routine vaccines in healthy infants and toddlers. Clin Vaccine Immunol. 2010 Apr 28.

29. French N, et al. A trial of a 7-valent pneumococcal conjugate vaccine in HIV-infected adults. N Engl J Med. 2010;362:812-22.

30. Kroon FP, et al. Enhanced antibody response to pneumococcal polysaccharide vaccine after prior immunization with conjugate pneumococcal vaccine in HIV-infected adults. Vaccine. 2000;19:886-94.

31. Lesprit P, et al. ANRS 114-Pneumovac Study Group. Immunological efficacy of a prime-boost pneumococcal vaccination in HIV-infected adults. AIDS. 2007;21:2425-34.

32. Peñaranda M, et al. Majorcan Pneumococcal Study Group. Conjugate and polysaccharide pneumococcal vaccines do not improve initial response of the polysaccharide vaccine in HIV-infected adults. AIDS. 2010;24:1226-8.

33. Scott DA, et al. Phase 1 trial of a 13-valent pneumococcal conjugate vaccine in healthy adults. Vaccine. 2007;25:6164-6.

34. Hung CC et al. ,A 5-year longitudinal follow-up study of serological responses to 23-valent pneumococcal polysaccharide vaccination among patients with HIV infection who received highly active antiretroviral therapy. HIV Medicine 2010; 11: 54-63.

# 35. Thanee C. et al. The immunogenicity and safety of pneumococcal conjugate vaccine in human immunodeficiency virus-infected Thai children. Vaccine. 2011; 29:5886-91

36. World Health Organization. Recommendation for the production and control of pneumococcal conjugate vaccines. WHO Technical Report Series. No. 927, 2005.
